# Non-target molecular network and putative genes of flavonoid biosynthesis in *Erythrina velutina* Willd., a Brazilian semiarid native woody plant

**DOI:** 10.3389/fpls.2022.947558

**Published:** 2022-09-08

**Authors:** Daisy Sotero Chacon, Marlon Dias Mariano Santos, Bernardo Bonilauri, Johnatan Vilasboa, Cibele Tesser da Costa, Ivanice Bezerra da Silva, Taffarel de Melo Torres, Thiago Ferreira de Araújo, Alan de Araújo Roque, Alan Cesar Pilon, Denise Medeiros Selegatto, Rafael Teixeira Freire, Fernanda Priscila Santos Reginaldo, Eduardo Luiz Voigt, José Angelo Silveira Zuanazzi, Kátia Castanho Scortecci, Alberto José Cavalheiro, Norberto Peporine Lopes, Leandro De Santis Ferreira, Leandro Vieira dos Santos, Wagner Fontes, Marcelo Valle de Sousa, Paulo Costa Carvalho, Arthur Germano Fett-Neto, Raquel Brandt Giordani

**Affiliations:** ^1^Department of Pharmacy, Federal University of Rio Grande do Norte (UFRN), Natal, RN, Brazil; ^2^Computational and Structural Proteomics Laboratory, Carlos Chagas Institute, Fiocruz, PR, Brazil; ^3^Stanford Cardiovascular Institute, Stanford University School of Medicine, Stanford, CA, United States; ^4^Plant Physiology Laboratory, Center for Biotechnology and Department of Botany, Federal University of Rio Grande do Sul, Porto Alegre, RS, Brazil; ^5^Bioinformatics, Biostatistics and Computer Biology Nucleus, Rural Federal University of the Semiarid, Mossoró, RN, Brazil; ^6^Institute for Sustainable Development and Environment, Dunas Park Herbarium, Natal, RN, Brazil; ^7^NPPNS, Department of Biomolecular Sciences, Faculty of Pharmaceutical Sciences of Ribeirão Preto, University of São Paulo (FCFRP-USP), Ribeirão Preto, SP, Brazil; ^8^Zimmermann Group, European Molecular Biology Laboratory (EMBL), Structural and Computational Biology Unit, Heidelberg, Germany; ^9^Signal and Information Processing for Sensing Systems, Institute for Bioengineering of Catalonia (IBEC), Barcelona Institute of Science and Technology, Barcelona, Spain; ^10^Institute of Biology, Leiden University, Leiden, Netherlands; ^11^Department of Cell Biology and Genetics, Center for Biosciences, Federal University of Rio Grande do Norte, Natal, RN, Brazil; ^12^Laboratory of Pharmacognosy, Federal University of Rio Grande do Sul, Porto Alegre, RS, Brazil; ^13^Chemistry Institute, São Paulo State University (UNESP), Araraquara, SP, Brazil; ^14^Genetics and Molecular Biology Graduate Program, Institute of Biology, University of Campinas, Campinas, Brazil; ^15^Laboratory of Protein Chemistry and Biochemistry, Department of Cell Biology, University of Brasilia, Brasilia, DF, Brazil

**Keywords:** *Erythrina velutina*, flavonoids, Caatinga, molecular network, transcriptome

## Abstract

*Erythrina velutina* is a Brazilian native tree of the Caatinga (a unique semiarid biome). It is widely used in traditional medicine showing anti-inflammatory and central nervous system modulating activities. The species is a rich source of specialized metabolites, mostly alkaloids and flavonoids. To date, genomic information, biosynthesis, and regulation of flavonoids remain unknown in this woody plant. As part of a larger ongoing research goal to better understand specialized metabolism in plants inhabiting the harsh conditions of the Caatinga, the present study focused on this important class of bioactive phenolics. Leaves and seeds of plants growing in their natural habitat had their metabolic and proteomic profiles analyzed and integrated with transcriptome data. As a result, 96 metabolites (including 43 flavonoids) were annotated. Transcripts of the flavonoid pathway totaled 27, of which *EvCHI, EvCHR, EvCHS, EvCYP75A* and *EvCYP75B1* were identified as putative main targets for modulating the accumulation of these metabolites. The highest correspondence of mRNA vs. protein was observed in the differentially expressed transcripts. In addition, 394 candidate transcripts encoding for transcription factors distributed among the bHLH, ERF, and MYB families were annotated. Based on interaction network analyses, several putative genes of the flavonoid pathway and transcription factors were related, particularly TFs of the MYB family. Expression patterns of transcripts involved in flavonoid biosynthesis and those involved in responses to biotic and abiotic stresses were discussed in detail. Overall, these findings provide a base for the understanding of molecular and metabolic responses in this medicinally important species. Moreover, the identification of key regulatory targets for future studies aiming at bioactive metabolite production will be facilitated.

## Introduction

*Erythrina velutina* (Fabaceae) is a pioneer tree from the Caatinga, a unique tropical dry forest located in the semiarid region of North-eastern Brazil (Rodrigues et al., 2018). *Erythrina* spp. is rich in metabolites of pharmaceutical interest, having sedative (Ozawa et al., 2008), anticonvulsant (Vasconcelos et al., 2007), and anxiolytic (Raupp et al., 2008) activities, which merit the launching of initial pre-clinical trials (Guaratini et al., 2014). Hitherto, ~91 alkaloids and 370 flavonoids were reported in various species of *Erythrina* (Fahmy et al., 2018, 2020), highlighting the remarkable chemical diversity of the main bioactive constituents of the genus.

Although *Erythrina* metabolites show diversity in chemical structures and biological potential, most of these bioactive natural products (NPs) are present in small amounts, which hampers extraction and detection by analytical techniques, and makes the process a major challenge for development of new products (Feitosa et al., 2012; Chacon et al., 2021b; Phukhatmuen et al., 2021). Alternatives to tackle low yields and excess of contaminants include metabolic engineering in microorganisms, semi-synthesis, and combinatorial biosynthesis, which can facilitate large-scale production of NPs (Atanasov et al., 2015). However, all these approaches require an extensive knowledge platform supported by basic research.

The study of biosynthetic regulation is paramount for improving target metabolite yields. Biosynthetic regulation may be initially examined by investigating the metabolic networks specific to different plant organs (Patra et al., 2013), particularly those involving production and/or accumulation of flavonoids and alkaloids, which besides being major compounds, are often responsible for the most useful bioactivities (Fahmy et al., 2018). In fact, multi-omics strategies, and their application directly impact the physiological control and regulation of bioactive compounds production by genetic and metabolic engineering (Guo et al., 2020; Helmy et al., 2020; Chen et al., 2021).

The first transcriptomic study involving *Erythrina velutina* was recently published by our group. This experimental approach enabled a first look at the genes associated with the chemical diversification process of isoquinoline alkaloids, as well as an overview of their putative biosynthesis pathways in the *Erythrina* genus, in particular of a medicinal native species of the Caatinga (Chacon et al., 2021b). Herein, our analysis is focused on flavonoid biosynthesis, another major class of NPs with important *in planta* roles and significant pharmacological interest.

Among their several functions, flavonoids are necessary for the interaction between N-fixing bacteria and leguminous plants during nodulation (Bosse et al., 2021), regulation of polar auxin transport (Yin et al., 2014), non-enzymatic antioxidant protection (Agati et al., 2012), and modulation of some hormonal signaling pathways (Brunetti et al., 2018). Flavonoids also perform several beneficial functions in human health, including antioxidant activity, improved memory acquisition and fear recovery (De Oliveira et al., 2014), as well as GABA modulation, playing an important role in cognitive processes (Wang et al., 2007). Although flavonoids are the largest class of recorded polyphenols (more than 8,000 metabolites identified), the current knowledge of their metabolism is essentially restricted to non-medicinal and model legume plants, particularly those that also produce isoflavonoids (Tohge et al., 2017; Wen et al., 2020). Therefore, little is known about the metabolism of flavonoids in *Erythrina* and studies at the molecular biosynthetic level are currently not available.

As part of our research efforts to dissect specialized metabolism in plants adapted to the harsh and particular conditions of the Caatinga, herein we applied integrated tools and carried out a comparative global analysis of the metabolites set present in *E. velutina* leaves and seeds, with a particular focus on the putative biosynthetic pathways of identified flavonoids. To that end, the vegetative and reproductive plant parts obtained from different natural populations were analyzed by non-directed metabolic profile with LC-HRMS/MS, next-generation RNA sequencing (RNA-seq), and gel-free proteomics. This study provides new insights into the regulation and bioprospecting of specialized metabolites of *Erythrina*, serving as a reference platform for understanding and modulating the unique biochemistry of plants from this little-known peculiar and biodiverse semiarid biome.

## Materials and methods

### Plant material

Harvesting and processing of plant material, metabolite extraction, and HR-LC-DAD-ESI-MS/AutoMS data acquisition were as described in Chacon et al. (2021b). Samples of seeds and leaves were harvested at daytime during the dry season from natural populations (Supplementary Table 1), composed of 5–10 trees of *Erythrina velutina* Willd in different locations and subjected to a multi-omics analyses approach (transcriptomics, proteomics, and metabolomics). A voucher specimen was deposited at the Herbarium of the Federal University of Rio Grande do Norte, Brazil, under the reference number UFRN16079. Authorization to harvest plant material was granted by SISBIO (327493) and access to the Brazilian genetic heritage by SISGEN (A8E4663). All samples were analyzed in four biological replicates.

### Non-target metabolite profile

#### LC-MS/MS analysis supported by molecular networking

Prior to the classical molecular networking workflow, the raw files with MS/MS data were converted to the mzXML format using the MSConvert (ProteoWizard) and processed using MzMine2 (http://mzmine.github.io/). Preprocessing was carried out for all samples in the following steps: mass calibration, mass detection (in both MS1 and MS2 levels), extraction of retention time (RT) information by chromatogram builder, peak deconvolution, mass grouping of isotopic patterns, and spectral alignment (Pluskal et al., 2010).

Following preprocessing, the resulting data was inserted into the Global Natural Products Social Molecular Networking Platform (GNPS) (Wang et al., 2016) to calculate spectral similarity, using a cosine score threshold of 0.7. The steps for this calculation were performed using the online workflow (https://ccms-ucsd.github.io/GNPSDocumentation/) on the GNPS website (http://gnps.ucsd.edu). Moreover, to carry out spectral correspondence against libraries in the GNPS, the mass tolerance of precursor ions was defined as 2.0 Da, whereas ion tolerance of fragments of MS/MS was set at 0.5 Da. The library spectra were filtered in the same way as the input data. All matches held between network spectra and library spectra required a score above 0.7 and at least 6 shared peaks.

### Transcriptome

The methodology procedures for total RNA extraction, cDNA library preparation and RNA sequencing using the Illumina technology were performed as described in Chacon et al. (2021b).

#### Bioinformatics analyses

Raw sequences were assembled using the software Trinity v2.11.0 (Haas et al., 2013). The resulting FASTA files were then built hierarchically to form pool, tissue, and plant assemblies, according to the scheme in Supplementary Table 2 (assembly-sizes). The transcripts were annotated using the EnTAP pipeline (Hart et al., 2020), with default parameters, against the NCBI's Plant RefSeq (O'Leary et al., 2016). The EnTAP includes similarity search across five repositories, protein domain assignment, ortholog gene family evaluation, frame selection, and translated (Transdecoder) with standard parameters of each tool. The sequences were subjected to a second run of BLASTx annotation against the UniProtKB/SWISS-PROT bank (https://www.uniprot.org/). A minimum of 30% identity, 50% coverage and 10^−10^ E-value were used as parameters.

To assess the genetic completeness of the RNA-seq dataset, we used the BuscoV3 tool v4.1.4|2020-10-01 (https://busco.ezlab.org/). Initially, the database was downloaded, filtered for classification viridiplantae_odb10.2020.-09-10.tar.gz, confronted with the set of identified transcripts, followed by the execution of tBLASTN to predict the orthologous proteins.

Transcription factors were predicted using the Plant Transcriptional Regulatory Map (Tian et al., 2019) - PlantTFDB v5.0 tool to search for the best hit in *Arabidopsis thaliana*. We performed the enrichment analysis of GO terms of the transcription factors using a *p*-value threshold ≤ 0.001 for the most significant nodes. In addition, transcription factors were singled out from this annotation and their presence was verified in both tissues with a local BLAST search.

Differential expression analysis was performed with the R limma package v3.48.3 (Ritchie et al., 2015). After analysis, the differentially expressed transcripts (DETs) were considered those with adjusted *p* < 0.01 and logFC > 2 or < −2. For DETs enrichment analysis, we initially mapped all *Erythrina velutina* transcript IDs to IDs with known *Glycine soja* Siebold and Zucc. ontologies (species with the best hits). After mapping, the GO terms and their respective *p-*values were inserted in the ReviGO tool v1.2 to detect the overrepresented terms, removing the obsolete and redundant GO terms using default criteria.

KofamKOALA was used to assign KEGG IDs to the translated transcripts (Aramaki et al., 2020); the presence of enzymes in metabolic pathways was assessed *via* the KEGG Search and Color Pathways online tool. Heatmaps were built in the TBtools software v1.09854 (Chen et al., 2020). The MapMan Version 3.6.0RC1 software (Usadel et al., 2009) was used to classify proteins in metabolic pathways, filtering the Gmax_189 dataset available in Phytozome v9.0 (https://phytozome-next.jgi.doe.gov/), followed by BLASTp. Other statistics and functional analyses were performed using R v.3.5.2, including graphical representations.

### Proteome

#### Extraction of proteins and sample preparation

Leaves and seeds of *E. velutina* from the four natural populations were powdered with mortar and pestle in liquid nitrogen (Supplementary Table 1), weighed (150 mg), and used for protein extraction with the Plant Total Protein Extraction Kit (Sigma Aldrich) as per the manufacturer's protocol. Protein concentration was estimated using the Bradford assay (Bradford, 1976). Each sample in technical triplicate was submitted to the reduction process (DTT 0.1 mol/L in Tris-HCl buffer 0.25 mol/L pH8.6), alkylation (iodoacetamide 0.05 mol/L) and digestion (trypsin 1:100 in 0.01 mol/L ammonium bicarbonate for 20 h). The obtained peptides were desalted in C-18 micro-columns and quantified using the Qubit system. Before analysis by LC-MS-MS, the efficiency of digestion was evaluated by MALDI-TOF.

#### Chromatography and mass spectrometry analysis

Peptides were analyzed in a chromatographic system (Dionex Ultimate 3000 RSLCnano UPLC, Thermo, USA), configured with a 3 cm x 100 μm trap column containing 5 μm, 120 Å C18 particles (ReprosilPur, Dr. Maich GmbH) connected in series to the 24 cm x 75 μm analytical column containing 3 μm, 120 Å C18 particles (ReprosilPur, Dr. Maich GmbH). The samples were injected to obtain 1 μg in the column, submitted to a linear elution gradient between solvents A (0.1% formic acid in water) and B (0.1% formic acid in acetonitrile) from 2% B to 35% B within 155 min.

The fractions separated in the chromatographic system were eluted directly into the ionization source of an Orbitrap Elite mass spectrometer (Thermo, USA), configured to operate in DDA (data-dependent acquisition) mode, and the MS1 spectra were acquired in the orbitrap analyzer, with a resolution of 120,000 and a range of *m/z* between 300 and 1,650. The 20 most intense ions, above the intensity limit of 3,000 were fragmented, generating MS2 spectra, in the CID ion trap analyzer (Kalli et al., 2013). The reanalysis of already fragmented ions was inhibited by dynamic exclusion (Andrews et al., 2011), favoring the identification of less abundant peptides.

#### Bioinformatics analyses

##### Peptide spectrum matching (PMS)

Data analysis was performed using the PatternLab for proteomics V software (PLV) (Santos et al., 2022), available at http://www.patternlabforproteomics.org/. The predicted protein sequences from the *Erythrina velutina* transcriptome dataset were used as the database and matched against raw files from the experimental proteome, including the 123 common contaminants from mass spectrometry. The Comet 2021.01 rev. 0 search engine was used for identifying the mass spectra (Eng et al., 2015). The search parameters considered: fully and semi-tryptic peptide candidates with masses between 500 and 6,000 Da, up to two missed cleavages, 40 ppm for precursor mass, and bins of 1.0005 m*/z* for MS/MS with an offset of 0.4. The modifications selected in the search were carbamidomethylation of cysteine and oxidation of methionine as fixed and variable, respectively.

##### Validation of PSMs

The validity of the PSMs was checked using the Search Engine Processor (SEPro) (Carvalho et al., 2012a). The identifications were grouped by charge state (2 + and ≥ 3 +) and then by tryptic status, resulting in four distinct subgroups. For each group, the XCorr, DeltaCN, DeltaPPM, and Peak Matches values were used to generate a Bayesian discriminator. The identifications were sorted in non-decreasing order according to the discriminator score. A cutoff score accepted a false-discovery rate (FDR) of 2% at the peptide level based on the number of decoys (Barboza et al., 2011). This procedure was independently performed on each data subset, resulting in an FDR independent of charge state or tryptic status. Additionally, a minimum sequence length of five amino-acid residues and a protein score >2 were imposed. Finally, identifications deviating by more than 10 ppm from the theoretical mass were discarded. This last filter led to protein level FDRs lower than 1% for all search results (Yates et al., 2012).

##### Proteomic data analyses

Quantification was performed according to Normalized Ion Abundance Factors (NIAF) in PatternLab. Four biological replicates (two from the city of Acari and two from the city of Jardim do Seridó) were independently quantified in both leaves and seeds, with two technical replicates each. As stringency parameters, proteins with at least 2 unique peptides were selected, common contaminants and decoys were included in the search. We also performed the differential analysis of the abundance using PatternLab's TFold module to compare leaves and seeds. The statistical filters were Benjamin Hochberg q-value (FDR) 0.05 and F-stringency 0.13 (Carvalho et al., 2012b).

##### Interaction network methodology

Initially, mapping of the ID codes of the differentially expressed transcription factors and transcripts of the flavonoid pathway in *Erythrina velutina* was performed in relation to *Arabidopsis thaliana* (L.) (3702). After retrieval, the IDs were entered into the String v11.5 platform (https://string-db.org/), selecting the organism *Arabidopsis thaliana*. The network type was defined as a full STRING network and minimum required interaction score of 0.500 confidence.

## Results

### MS-based metabolomics of *Erythrina velutina*

**Figure 1:**
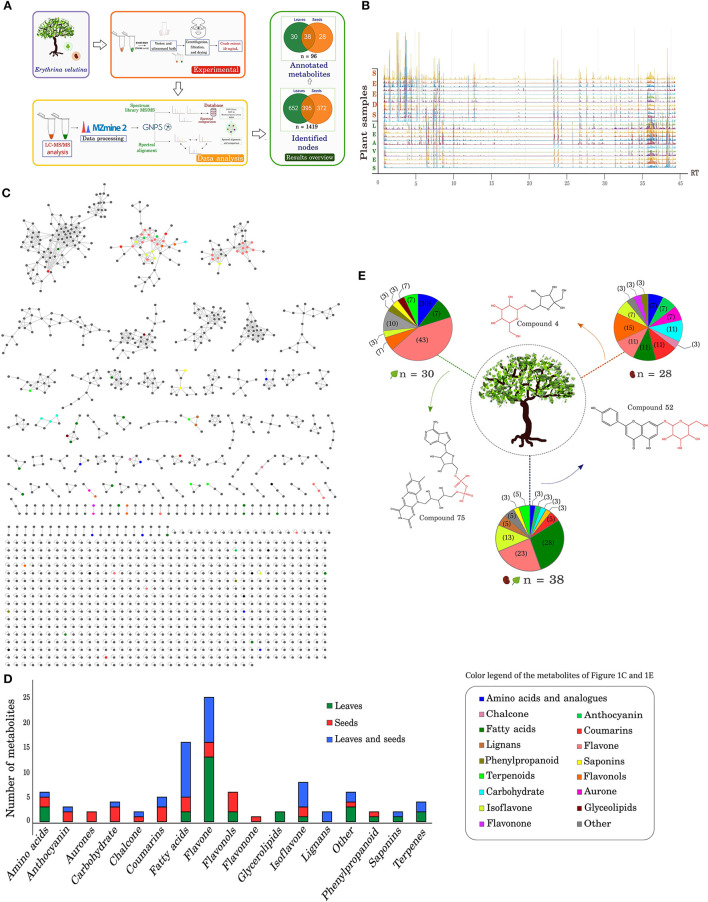
Overview of the metabolic profile in leaves and seeds of *Erythrina velutina*. **(A)** Experimental procedure and data analysis by LC/MS. The metabolites number identified in the leaves and seeds are indicated in the Venn diagram, in green and orange colors, respectively. **(B)** Chromatogram overlay and four biological replicas and three technical replicas of *E. velutina* leaves and seeds. The X axis represents the retention time of the compounds; The Y axis represents the different samples. **(C)** Total molecular network generated after analysis *via* GNPS. The following parameters were set: mass tolerance of precursor ions defined as 2.0 Da, ion tolerance of fragments of MS/MS as 0.5 Da, and a cosine score above 0.7. Different colors represent different identified metabolic classes, as indicated in the caption frame. **(D)** Number of annotated metabolites and distributed in the different classes identified. Metabolites exclusively identified in leaves and seeds are represented in green and red bars, respectively; metabolites observed in both structures are represented in blue. **(E)** Distribution of metabolite classes in leaves and seeds. Thirty compounds were exclusive to leaves, 28 were exclusive to seeds, and 38 compounds were common to both structures. The depicted structures represent identified compounds unique and common to leaves and seeds, highlighting glycosylated and phosphorylated molecules identified in the metabolome.

In our previous study, different accumulation profiles of alkaloids were recorded between leaves and seeds of *E. velutina*, allowing the outline of their putative biosynthesis routes (Chacon et al., 2021b). The heterogeneity of alkaloids, their molecular diversity, and the extreme environmental conditions in the Caatinga biome where the species grows motivated us to systematically identify and compare the set of metabolites present in leaves and seeds, an approach not yet reported for this class of NPs in the genus *Erythrina*. Eight crude extracts from *E. velutina* (four from leaves and four from seeds), were analyzed by LC-HRMS, in positive ionization mode. The MS/MS data, once converted in mzXML and processed in Mzmine2, was uploaded to the GNPS platform for spectral similarity calculation and molecular network (Figure 1A).

### Chromatogram analysis

Variability of the chemical profile in the different plant structures and samples was investigated to check for replicate consistency and part specific signatures. Chromatogram analyses revealed homogeneous profiles among the replicates and highly similar ones in apolar compounds presence (RT > 15 min, Figure 1B). In seeds, the major compounds eluted in the beginning of the chromatographic run (0 < RT <5 min) were mostly consistent with coumarin and its derivatives (e.g., rutarin*)*, carbohydrates (maltotriose, isomaltulose), chalcone, and flavonols (compound 11). In leaves, the major metabolites eluted between 6 and 10 min of analysis and were characterized as flavones (luteolin-6-*C*-glucoside, apigenin-8-*C*-glucoside, and compound 33), isoflavone (daidzein-8-*C*-glucoside), phenylpropanoid (compound 27), and amino acid (compound 38). Details of annotated metabolites can be found in Supplementary Table 3.

Of all identified metabolites, 70% (67 annotated compounds) eluted in the beginning of the chromatographic run (RT <20 min). In this range, the numbers of metabolites distributed in leaves and seeds were similar, but with different proportions of metabolic classes between the plant structures. For example, the number of unique metabolites of seeds and leaves corresponded to 25 and 22 annotated compounds, respectively, and 20 were common to both plant parts. However, 64% of the leaf-exclusive metabolites were flavones and amino acids. In seeds, the exclusive presence of flavones, isoflavone, flavonols, carbohydrates, amino acids, and chalcone represented 68%. Among the metabolites present in both leaves and seeds about 70% were flavones and isoflavones.

The 29 compounds that eluted at the final stage of the chromatographic run (RT > 20 min) were distributed in different proportions. In total, 18 metabolites were present in the two plant structures, whereas 8 and 3 exclusive metabolites were observed in leaves and seeds, respectively. The main metabolic class found after elution in this final stage corresponded to fatty acids, representing 100% of the seed exclusive metabolites (compounds 82, 83, and 88), 50% of the leaf exclusive metabolites, along with glycolipids (compounds 78, 79, 95, and 96), and 61% of the metabolites common to both plant structures (compounds 68, 71, 72, 80, 81, 85, 86, 87, 90, 92, and 94). Lignans, a terpene, coumarin, and triterpene saponin were also annotated.

### Network of molecules generated in GNPS

The annotation of metabolites, their interrelationship, and similarity analysis from large MS/MS data sets generates a vast amount of information from LC-MS/MS. Molecular network may be used as a tool to organize voluminous data sets and spot differences between groups (Pilon et al., 2019; Demarque et al., 2020).

In total 1,419 nodes were detected and about 7% of the nodes (96 metabolites) were annotated using the GNPS spectral libraries (Figures 1C,D). A total of 28 compounds were exclusively annotated in seeds, 30 in leaves, and 38 entities for both structures (Figure 1E). In general, 90% of the detected *m/z* corresponded to the value range *m/z* 192–595, 38% of these present in both leaves and seeds, whereas 33 and 29% were exclusive to leaves and seeds, respectively. Alkaloids from the alkenoid and dienoid subclasses had also been identified at the same RT ranges in our previous work. To better visualize the molecular network, alkaloid clusters were removed (see details and discussion in Chacon et al., 2021b). A 2-factor analysis of multiple pairwise comparisons of leaves and seeds showed that significant differences in retention times were observed between metabolites exclusive to leaves with higher and lower *m/z* ratios. Relevant differences were also recorded for metabolites with a lower mass between the group of exclusive compounds and that common to both leaves and seeds (Supplementary Figure 1).

The 96 annotated metabolites were distributed in different classes, including primary and secondary metabolism consisting of 25 flavones, 16 fatty acids, 8 isoflavones, 6 flavonols, 6 amino acids and analogs, 5 coumarins and derivatives, 4 terpenes and derivatives, 4 carbohydrates, 3 anthocyanins, 2 saponins, 2 phenylpropanoid derivatives, 2 lignans, 2 aurones, 2 chalcones, 2 glycerolipids, 1 flavonone, and 6 other compounds (Figure 1C and Supplementary Table 3).

### Chemical profile differences between leaves and seeds

The global comparative analysis of metabolite profile in leaves and seeds of *E. velutina* showed some differences (Supplementary Figures 4–7). Among the 30 leaf-specific annotated metabolites, flavones (e.g., 7,4'-dihydroxyflavone, flavonoid 8-*C*-glycosides) constituted the greatest share of compounds, representing 43%, followed by amino acids (compounds 14 and 16), terpenes (compounds 24 and 84), and fatty acids (compounds 79 and 95), with smaller proportions (~10%) (Figure 1E). In this group, a triterpene saponin (soyasapogenol B), two glycerolipids (compounds 78 and 96), a phenylpropanoid (compound 27), and an isoflavone (compound 41) were also identified.

In contrast, flavonols (compounds 11, 21, 23, and 26) were the predominant seed-specific compounds, representing about 15% of the 28 metabolites annotated. Similar numbers of metabolites were found in the flavone pathway (compounds 34, 55, and 66), coumarin and derivatives (compounds 12, 54, and 56), carbohydrates (compounds 2, 3, and 4), and fatty acids (compounds 82, 83, and 88) (11% each). A flavonone that was found exclusively in seeds was annotated as dihydrotricetin. The compounds common to both plant structures seem to comprise mainly flavones (apigenin-8-*C*-glucoside, diosmetin), isoflavones (daidzein-8-*C*-glucoside, compound 50, 61, and 62), and fatty acids (e.g., compounds 68, 71, and 72).

### Metabolites identified in the molecular networking linked to glycosyl and phosphate groups

Considering the importance of glycosyl groups in structures of multiple specialized metabolites and the participation of phosphate in signaling cascades, a closer look was taken at metabolites containing such groups. Several of the annotated compounds had glycosyl portions, especially secondary metabolites, including 100% of the anthocyanidins (forming anthocyanins), 80% of the flavones, 75% of the isoflavones, and 67% of the flavanols. This total corresponds to 40 glycosylated secondary metabolites, 27% present in both structures. Our previous work reported the great diversity and high number of transcripts that encode glycosyltransferases, many of which act in secondary metabolism, including flavonoid biosynthesis (Chacon et al., 2021b). Moreover, it is important to mention that we have also identified fatty acids linked to a phosphate group (80, 81, 82, 83, and 90).

## Transcriptome and proteome

### Strategy used and functional annotation

**Figure 2:**
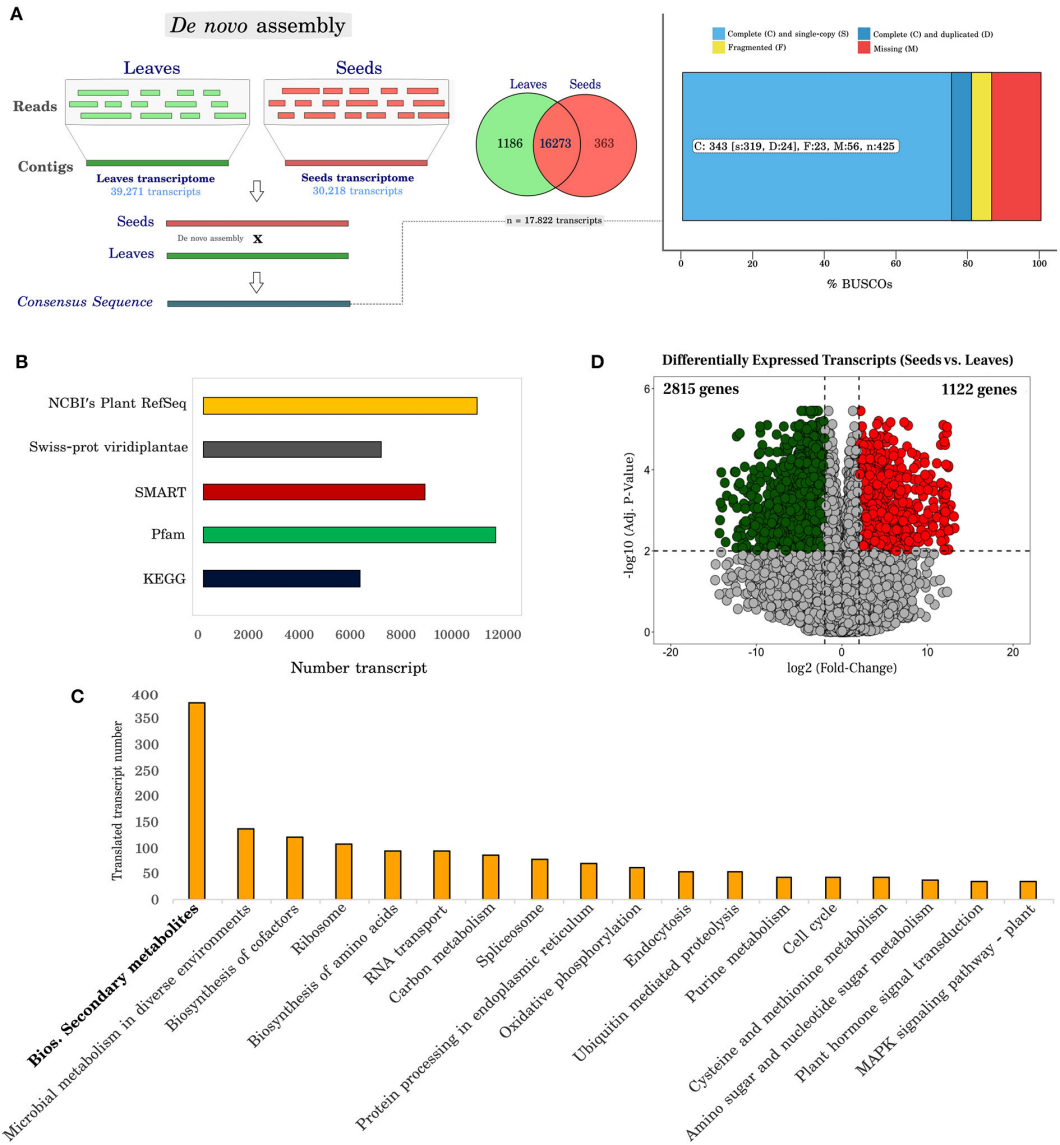
Functional annotation and differentially expressed transcripts in *Erythrina velutina*. **(A)** Strategy to assemble cDNA libraries from leaves and seeds and analyses the genetic content of orthologs using the BuscoV3 tool. The raw files of each individual lane were used for the sequence assembly step, using Trinity software. The FASTA files resulting from this process were then constructed hierarchically to form pool sets (collection of different populations), and later tissues (leaves and seeds), according to the scheme in Supplementary Table 2. The genetic completeness of the transcriptome using BUSCO is given as a percentage after analysis against the viridiplantae_odb10 database. In this analysis the X axis represents the cDNA library (leaves and seeds, combined; or separate leaves and seeds, [see Supplementary Figures 2, 3]), while the Y axis represents the percentage of complete, fragmented, and lost sequences in our analyses. After gathering the sequencing data from *E. velutina* leaves and seeds, 847 orthologs distributed in 425 groups were found in the dataset. Furthermore, with regard to the total representation of the analyzed transcripts, 343 (~80%) of the sequences were complete, 23 (~10%) fragmented and 56 (~5%) missing; **(B)** Number of transcripts annotated in the different databases used in this analysis; **(C)** Number of transcripts identified and distributed in the main identified metabolic pathways using The Kyoto Encyclopedia of Genes and Genomes (KEGG) tool; **(D)** Volcano plot representative of differentially expressed transcripts. The Y axis represents the adjusted *p*-value, and the X axis, fold change (log_2_). Up-regulated and down-regulated genes are red and green, respectively.

After the individual assembly of leaf and seed sequences, to obtain longer sequences and to better identify the genes and their functional products, a combined assembly to contemplate reads of both plant structures and search for similarities with information available in databases was carried out (Figure 2A). The individual assembly resulted in 39,271 transcripts in leaves with an average size of 1,221 bp (N_50_ = 1,652 bp) and 30,218 transcripts in seeds with an average size of 1,203 bp (N_50_ = 1,606 bp); in contrast, we obtained 17,822 transcripts with an average length of 1,504 bp contig in the joint assembly. The mean N_50_ value was 1,882 bp (Supplementary Table 4).

The overall data provide information on metabolic processes, making it possible to suggest molecular functions. For non-model species, such as *E. velutina*, it is rather important to submit sequences for annotation in different biological systems databases, with both automated and manual curation. In this work, transcripts resulting from the combined assembly of leaves and seeds were selected for different annotation steps (Figure 2B). The highest percentage of annotation (60.14%) was obtained in the NCBI Plant RefSeq database (10,719 transcripts). For comparison, a parallel annotation of the transcripts was performed against the Swiss-Prot database, classifying for the Viridiplantae taxonomy, resulting in 6,975 (39.13%) annotated transcripts, of which 1.64% (293 transcripts) were identified as unique to this database. In addition, 38.21% of the data did not show significant similarity to other species sequences in these databases.

#### Functional identification of KEGG paths

The Kyoto Encyclopedia of Genes and Genomes (KEGG) tool was used to reconstruct a network of metabolic pathways. In total, 6,316 translated transcripts were annotated and assigned to at least 400 pathways. The category with the highest number of transcripts was biosynthesis of secondary metabolites (381 transcripts) (Figure 2C and Supplementary Table 5), having as main representative's flavonoids (including flavone, flavonols and isoflavonoid), terpenoids, phenylpropanoids, isoquinoline alkaloids, sesquiterpenoids, triterpenoids, and monoterpenoids.

#### Identification and classification of differentially expressed transcripts

We detected 6,448 expressed transcripts with an adjusted *p* < 0.01, being 3,937 recorded with fold-change variation (log_2_FC) > 2 and < −2 (2,815 down-regulated and 1,122 up-regulated in seeds) (Figure 2D). Next, we used the differentially expressed transcripts (DETs) to perform Gene Ontology (GO) enrichment analysis, especially on the identification of transcripts involved in flavonoid biosynthesis and transcription factors of *E. velutina*.

#### GO enrichment analysis of differentially expressed transcripts

After investigating representative subsets of major biological processes and molecular functions of regulated genes, we mapped 144 enriched GO terms to up-regulated genes (Supplementary Table 6), and 438 enriched GO terms to down-regulated genes (Supplementary Table 7), both in seeds. The GO terms and their respective *p*-values were incorporated into the reviGO web platform. We obtained 108 and 19 non-redundant GO terms of metabolic processes and molecular functions in the up-regulated category, respectively. For the downregulated category, 277 and 79 non-redundant GO terms of metabolic process and molecular functions were recorded, respectively.

**Figure 3:**
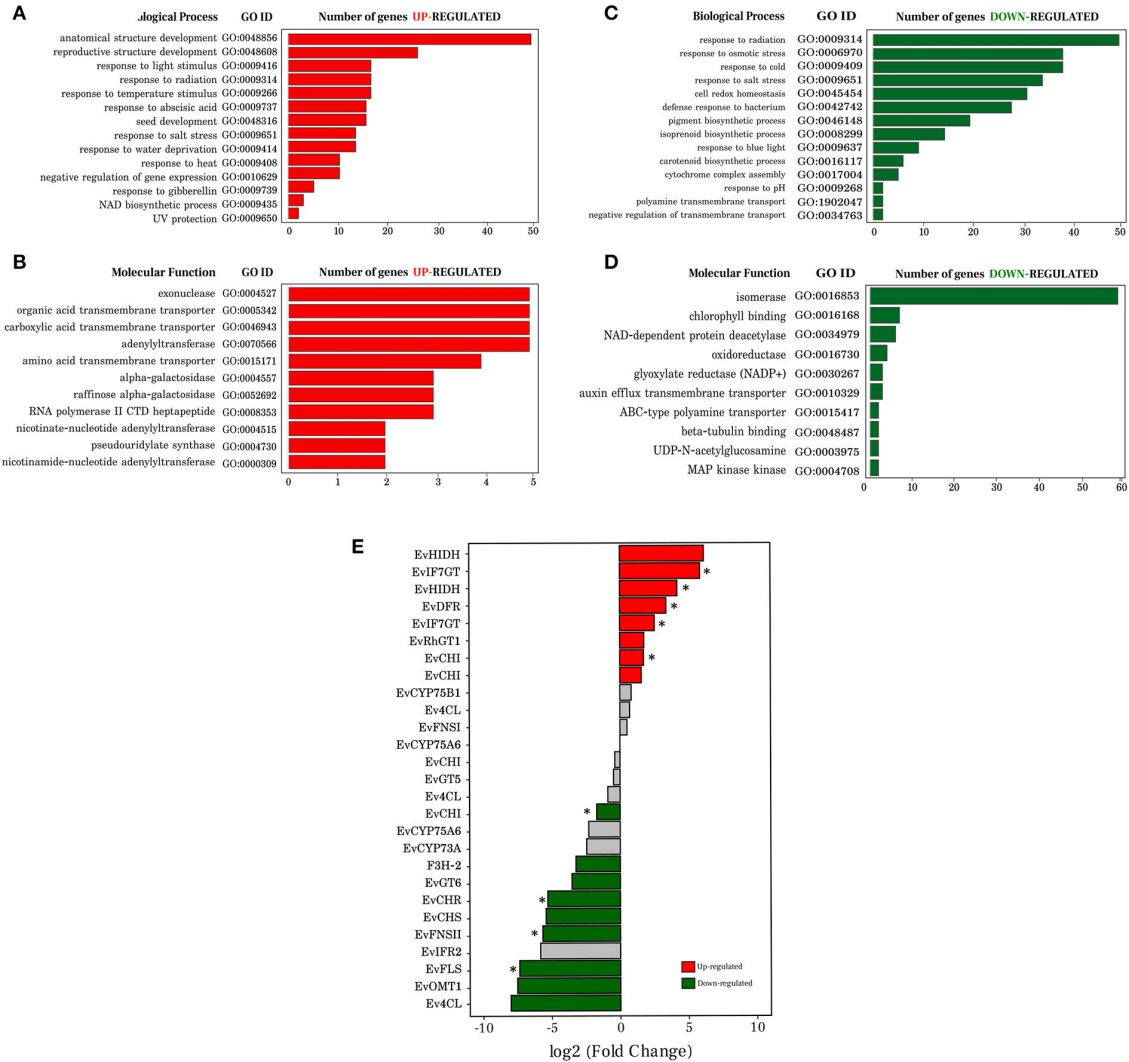
Gene ontology analysis of differentially expressed transcripts and expression profile of transcripts involved in the flavonoid biosynthesis pathway in *Erythrina velutina*. **(A,B)** Representation of the biological processes and molecular function, respectively, of the up-regulated transcripts. The ontology name, gene ID and amount of transcript identified in each GO term are also shown; **(C,D)** Representation of the biological processes and molecular function, respectively, of the down-regulated transcripts. The ontology name, gene ID and amount of transcript identified in each GO term is also depicted; **(E)** Bar graph representing the transcripts identified and involved in the flavonoid biosynthesis pathway. Transcripts marked in red and green bars, indicate that they were up- and down-regulated, respectively; transcripts represented by gray bars had no changes. Bars marked with an asterisk indicate that they have adjusted *p* < 0.01, unmarked bars have adjusted *p* < 0.05.

In the biological process category (Figure 3A) and molecular function (Figure 3B) of the up-regulated transcripts in seeds, identified GO terms were related to abiotic responses (UV, temperature, heat) and phytohormones (gibberellin, abscisic acid) (Figure 3A). These stimuli and regulatory molecules are known to trigger or associate with defense against stresses, as well as seed development and maturation. Although up regulation of some genes in the stress-related categories in seeds was observed, it occurred to a lesser extent than in leaves. Biosynthetic processes were also identified: the general term NAD biosynthetic process, which includes pyridine nucleotide and nicotinamide nucleotide, nucleobase-containing compound biosynthetic process, organic cyclic compound biosynthetic process, and aromatic compound biosynthetic process (Supplementary Table 6). As expected, up-regulated transcripts in seeds were related to several processes of development (seed, anatomical structure, and reproductive structure). The main molecular functions associated with this group were transporter, transferase, and synthase activities (Figure 3B).

In line with the leaf physiological role in photosynthetic metabolism, enriched terms related to pigment biosynthetic process, carotenoid, isoprenoid, and chlorophyll were up-regulated in this organ, and down-regulated in seeds (Figure 3C). A greater number of negatively down-regulated genes in seed pathways related to photosynthesis (photosystem II assembly, photosynthetic electron transport chain, regulation of photosynthesis, light reaction, photosystem I stabilization), amino acid metabolism (tryptophan biosynthetic process, methionine biosynthetic process, lysine biosynthetic process), and cell redox homeostasis (Supplementary Table 7). The molecular function associated with isomerase activity was highly down-regulated in seeds (Figure 3D). In addition, among the down-regulated genes in seeds there were some corresponding to four GO terms of molecular function involved in the biosynthesis, storage, and transport of metabolites: oxidoreductase activity, auxin efflux transmembrane transporter activity, ABC-type polyamine transporter activity, and MAP kinase activity (Figure 3D).

#### Identification of transcripts involved in flavonoid biosynthesis

In the large class of specialized metabolism process, flavonoids were one of the most enriched pathways, well-represented both at the level of metabolites and transcripts. In the *E. velutina* subfamily Papilionoideae (Fabaceae), most studies are associated with the ability of this clade to synthesize isoflavonoids (Azani et al., 2017); however, no gene that contributes to the accumulation of flavonoids in *E. velutina* is known. Based on the bioinformatic analysis of the transcriptome, a total of 27 transcripts encoding enzymes that act in the biosynthesis of flavonoids were identified as being similar in different species, included in the top 3: *Glycine soja, Glycine max* and *Arabidopsis thaliana*. This is not surprising since the genus *Glycine* is also part of the legume family like *Erythrina*, and all of the three hit species have high flavonoid content.

To explore gene expression profiles, these 27 identified transcripts involved in the flavonoid biosynthesis pathway were submitted to comparative analysis between seeds and leaves. Seventeen transcripts were identified as differentially expressed, of which eight were up-regulated and nine down-regulated in seeds. Genes that encode *EvIF7GT* transcripts (log2FC 5.85 and 2.53), *EvHIDH* (log2FC 4.19 and 6.13), *EvDFR* (log2FC 3.38), *EvRhGT1* (log2FC 1.73) and *EvCHI* (log2FC 1.57 and 1.73) were up-regulated in seeds, whereas the transcripts *EvCHI* (log2FC−1.71), *EvF3H-2* (log2FC−3.24*), EvGT6* (log2FC−3.56), *EvCHR* (log2FC−5.30), EvCHS (log2FC−5.43), *EvFNSII* (log2FC−5.67), *EvFLS* (log2FC−7.37), *EvOMT1* (log2FC−7.32) and a transcript *Ev4CL* (log2FC−8.00) were up-regulated in leaves (Figure 3E). Gene definition, KO number, identified functional domain, genetic ontology, identity, and e-value, are listed in Supplementary Table 8.

#### Transcription factors analysis

**Figure 4:**
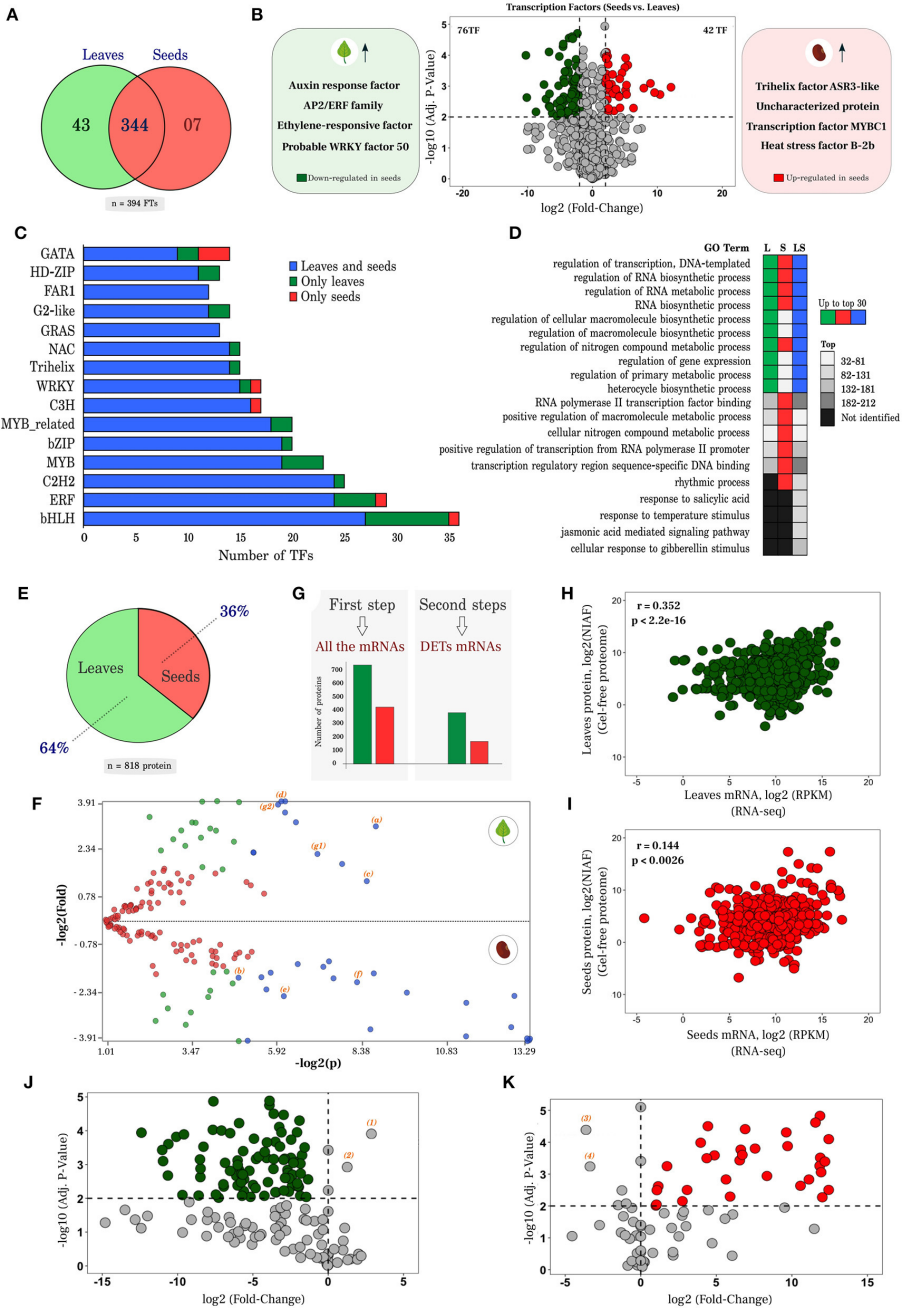
Analysis of transcription factors (TFs), proteome, and correlation of mRNA vs. proteins. **(A)** Transcription factors identified in leaves and seeds, in a unique and common way through the PlantRegMap platform; **(B)** Volcano plot of the up- and down- regulated TFs. The Y axis represents the adjusted *p*-value, and the X axis, fold change (log2). **(C)** Top15 of the main transcription factor families according to the number of transcripts identified. Blue bars represent probable transcripts encoding TF proteins in both leaves and seeds, while green and red bars indicate TFs uniquely found in leaves and seeds, respectively. **(D)** GO terms of transcription factors associated with different plant parts. L and S indicate the terms identified in the exclusive transcription factors of leaves and seeds, while LS, marks those present in both structures. Colors as in C; **(E)** Proportion of 818 proteins that fit the strongest parameters arising from proteomic analysis, through PatternLab (maximum parsimony, at least 2 unique peptides, search with inclusion of contaminants and baits); **(F)** Differentially abundant proteins identified from the proteome of *E. velutina* (leaves vs. seeds). Each point represents a protein identified according to its *p*-value (log_2_) on the X axis vs. fold change (log_2_) on the Y axis. Red dots are proteins that do not satisfy the fold change cut and *q*-value cut; green dots are proteins that satisfy the fold change but not the q value cut. Finally, blue dots are proteins that satisfy all statistical filters and those we consider statistically differentially abundant; **(G)** Total amount of proteins that were correlated with all mRNAs and with differentially expressed transcripts; **(H,I)** Correlation analysis of transcriptome vs. proteome, in leaves and seeds, respectively. The X axis represents the expression values of the transcripts in log_2_ (RPKM), while the Y axis indicates the normalized Ion abundance factors in log_2_ (NIAF) originating in the proteome; **(J,K)** Dot plot of unique mRNAs in leaves and seeds, respectively, that had proteins found with significant NIAF. The Y axis represents the adjusted *p*-value and the X-axis warp change (log2).

For familiar transcription factors with a high degree of manual and experimental curation, transcript sequences were submitted to the PlantTFDB v5.0 database. A group of 394 putative transcription factors (Figure 4A) distributed in 47 families was identified. Analysis of differential expression of the 394 TFs resulted in 76 and 42 down- and up-regulated in seeds, respectively (Figure 4B). Among the most dominant families were TFs regulating genes involved in responses to abiotic stress and in biosynthesis of specialized metabolites, including flavonoids. Auxin response factor (AT5G37020.1), APETALA2/ETHYLENE RESPONSIVE FACTOR (AP2/ERF) family transcription factors (AT5G07580.1) and ethylene-responsive transcription factor (AT1G19210.1) were expressed 10.26, 8.74, and 8.50 times higher in leaves than in seeds, respectively. On the other hand, Trihelix transcription factor ASR3-like (AT2G42280.1), heat stress transcription factor B-2b (XP_014521482.1) and an uncharacterized protein (NP_001267497.1) of the AP2/ERF family similar to TFs responsive to ethylene and dehydration in *Arabidopsis thaliana* were 12, 10 and 5 times more expressed in seeds than in leaves, respectively (Figure 4B and Supplementary Table 9). In Figure 4C, the top 15 members of different families of 394 putative transcription factors detected in this study are listed as a function of the number of identified genes. The main families were bHLH, ERF, C2H2, and MYB (Supplementary Table 10).

In the bHLH family, 36 TFs with basic helix-loop-helix domain were recorded, including MYC2 (AT1G32640.1), FBH4 (AT2G42280.1) and six differentially expressed, all down-regulated in seeds: two transcripts UNE12 (AT4G02590.2), one transcript PIF4 (AT2G43010.2), one transcription factor ILR3-like (AT5G54680.1), one transcription factor NAI1-like (XP_027907442.1) and a basic helix-loop-helix encoding gene (BIGPETAL, BPE). The AP2/ERF superfamily of transcription factors is the ERF family, which in turn can be divided into two essential subfamilies: ERF and CBF/DREB (Nakano et al., 2006). It was possible to identify 29 members of the ERF family, including: ERF9 (AT5G44210.1) and ERF12 (AT1G28360.1), in addition to 3 putative DETs, two from the ERF subfamily [ERF4 (AT3G15210.1) and ERF6 (AT4G17490. 1)] and one member of the DREB subfamily (AT1G01250.1). Four transcripts encoding 2 transcription factor proteins were also identified as differentially expressed within the C2H2 family, the third most enriched family registered, i.e., STOP1 (AT1G34370. 2) and ZFP4 (AT1G66140.1).

Twenty-three putative transcripts were identified as MYB TFs in leaves and seeds, including MYB14 (AT2G31180.1), MYB111 (AT5G49330.1) and MYB73 (AT4G37260.1). Only MYB111 was regulated showing a log2FC−3.52. However, it had an adjusted *p-*value of 0.18, so none of these transcripts was detected as differentially expressed. To investigate representative subsets of the main biological processes, 316 GO terms enriched in the PlantRegMap were mapped. The GO terms were listed according to their respective *p-*value; most biosynthetic and metabolic processes ranked within the top 30. Furthermore, processes related to environmental stimuli were also identified, and listed after the 30 initial positions in the ranking, including rhythmic processes, response to salicylic acid, response to temperature, jasmonic acid mediated signaling pathway, and cellular response to gibberellin stimulus (Figure 4D).

In addition, candidate transcripts encoding for transcription factors distributed among the bHLH, ERF, and MYB families were examined for potential interactions with genes of the flavonoid pathway. Based on interaction network analyses, several putative genes of the flavonoid pathway and transcription factors were related, particularly TFs of the MYB family (Supplementary Figures 13, 14).

#### Proteome analysis and differentially abundant proteins

In total, 1,152 and 749 proteins were identified in leaves and seeds, respectively (Supplementary Table 11). After concatenating, 1,762 proteins were obtained, of which 818 (64% in leaves and 36% in seeds) fit the stringency parameters (maximum parsimony, at least 2 unique peptides, searching with the inclusion of the contaminants and decoys) (Figure 4E). A search for differential abundance of proteins identified in leaves vs. seeds revealed that 33 proteins satisfied the statistical filters (statistically differentially abundant) when comparing the two plant parts (dots in blue, Figure 4F).

Two proteins involved in redox homeostasis peroxiredoxin-2E (a) and superoxide dismutase [Fe] (b) (Figure 4F and Supplementary Table 12) were up- and down-regulated, respectively, in leaves. Proteins related to carbon and nitrogen primary metabolism that impact the production of specialized metabolites were also identified, including aconitate hydratase (c), phosphoglycerate kinase (d), aspartate aminotransferase (e) and fructose-bisphosphate aldolase **(**f). Two isoforms of 14-3-3-like protein were up-regulated in leaves (g1 and g2). The 14-3-3 proteins are known to function as phosphosensors in signal transduction of hormonal, environmental stimuli, and stress responses (Camoni et al., 2018).

#### Correlation analysis of identified transcripts and proteins

To investigate the correlation of the identified transcripts in the RNA-seq dataset with proteins identified in the gel-free technique proteome, only those proteins that had significant NIAF (Normalized Ion Abundance Factors) were selected. Correlation analysis was performed in two steps (Figure 4G). In the first, all the mRNAs that were correlated with 431 proteins present in seeds and 748 proteins present in leaves that had significant NIAF (NIAF/RPKM) were considered. Pearson correlation analysis of proteome and transcriptome data yielded significant (p < 0.005), but weak correlation coefficient (*r* = 0.352 for leaves and *r* = 0.144 for seeds) (Figures 4H,I). In a second complementary analysis, only the differentially expressed transcripts were taken in account. Using this approach, 388 proteins in leaves and 170 proteins in seeds were correlated, showing a similar pattern to that of the first analysis (Supplementary Figures 8, 9). Overall, a significant and positive correlation was observed. Figures 4J,K represent the mRNAs that had their respective proteins with significant NIAF and present exclusively in plant structures. A subset of 86 and 32 unique RNAs had protein correspondence in leaves and seeds, respectively.

## Discussion

To establish a comprehensive view of *E. velutina* chemistry, the metabolic profile was obtained from plants growing in their natural habitats, deep into the Brazilian Caatinga biome. As an initial strategy to gain insight on this subject, different plant parts (leaves and seeds) were examined. The comparison between the different plant parts showed leaves having a higher number of annotated compounds, in line with their active metabolism and photosynthetic capacity. Like many woody species of Fabaceae, mature seeds of *E. velutina* are of the orthodox type and display dormancy due to the presence of impermeable teguments (Dos Santos et al., 2013). Despite their low metabolic activity in the dormant dry state, seeds appeared to be more metabolically diversified structures since more molecule classes were detected in their composition. This fact suggests the potential of greater activation of distinct metabolic processes during the development and maturation of this reproductive structure. This is somewhat expected since seeds bear complex organization and have the potential to give rise to a whole new plant, possessing a fully developed embryo with all of its fundamental tissues, besides metabolic reserves, defense molecules, and outer layers with developed cuticles, waxes and phenolics (Tan et al., 2013).

Investigating biosynthetic pathways is a challenge, as the balance of coordination of gene expression and metabolic state are not always associated. This is especially the case when the location of biosynthesis and accumulation of metabolites are different (Delli-Ponti et al., 2021). Nicotine, for example, is biosynthesized in roots, exported to shoots, and stored in leaf vacuoles (Chen et al., 2016). In addition, no species of the genus *Erythrina* has a complete genome or genome draft, so the present data has been organized by *de novo* assembly. After individual assembly of leaves and seeds, 17,822 sequences were generated. Efforts were made to carry out careful assembly of the data, but still more than 38% of the sequences could not be annotated. However, the data herein reported can be useful for genus functional transcriptomics/genomics, investigation of gene and/or protein candidates participating in specialized metabolism pathways, as well as database enrichment. Probably, greater precision in assembling from a well-annotated reference genome will be viable soon.

**Figure 5:**
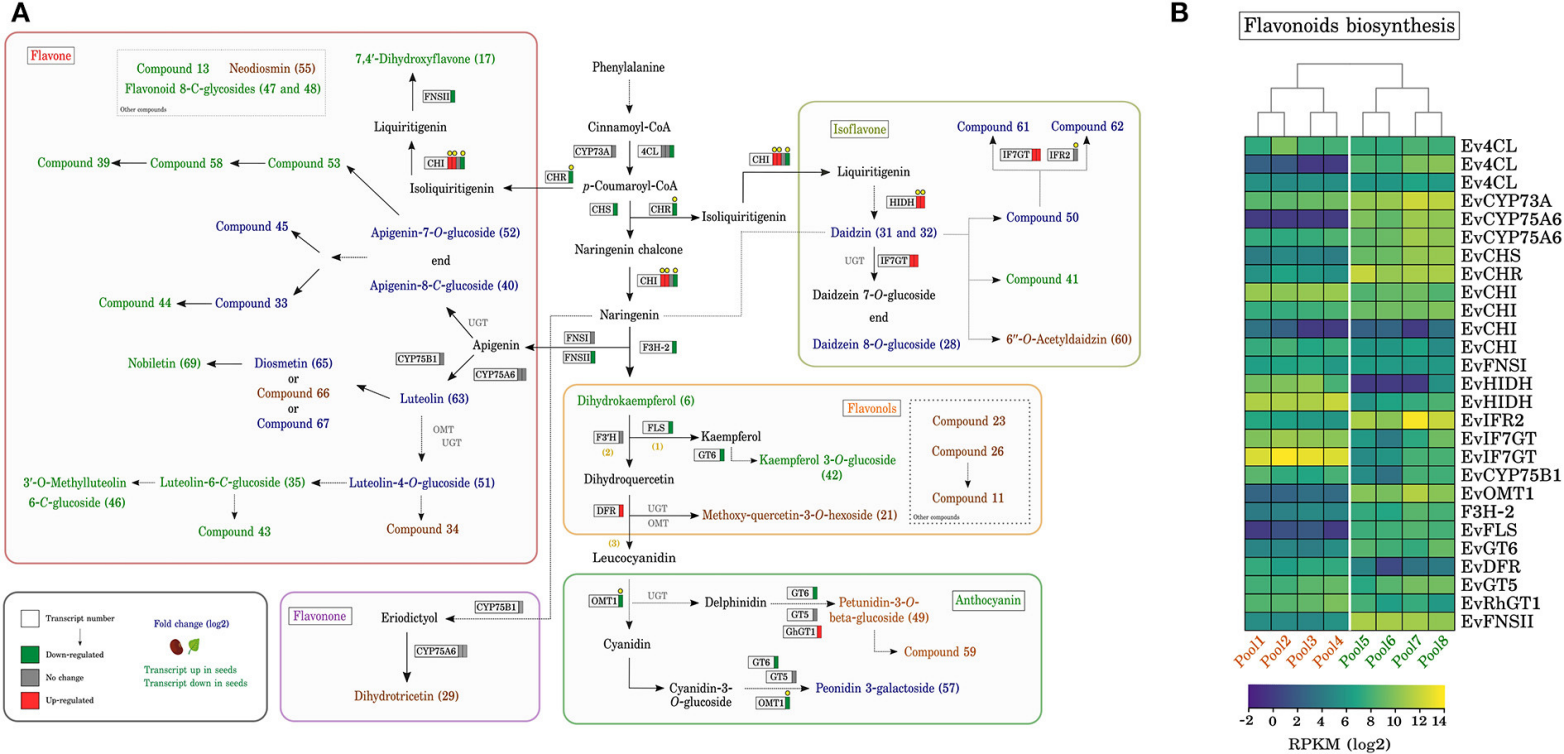
Flavonoids biosynthesis pathway in *Erythrina velutina*. **(A)** Proposed flavonoid pathway in *E. velutina*. Each frame corresponds to an identified transcript, with red and green bars indicating up- and down-regulated, respectively, while gray bars indicate no change. Flavonoids written in blue were annotated in both plant structures, while the shown in orange and green represent those found only in seeds or leaves, respectively. Transcripts marked with yellow circles are those with identified protein matches, with significant NIAF. **(B)** Expression pattern of candidates for the biosynthesis pathway of different classes of flavonoids. Pools from 1 to 4 represent biological replicates of seeds, while pools from 5 to 8 represent different replicates of leaves. The color scale shows the fold change value in RPKM. *Ev4CL*, 4-Coumarate–Coa ligase; *EvCYP73A*, Trans-cinnamate 4-monooxygenase; *EvCHS*, Chalcone synthase; *EvCHR*, Chalcone reductase; *EvFNSI*, Flavone synthase 1; *EvHIDH*, 2-Hydroxyisoflavanone dehydratase; *EvIF7GT*, Isoflavone 7-O-glucosyltransferase; *EvIFR2*, Isoflavone reductase; *EvCHI*, Chalcone isomerase; *EvF3H-2*, Flavanone 3-dioxygenase 2; *EvFNSII*, Flavone synthase 2; *EvCYP75B1*, Flavonoid 3'-monooxygenase; *EvFLS*, Flavonol synthase; *EvGT6*, UDP-glucose flavonoid 3-O-glucosyltransferase 6; *EvDFR*, Dihydroflavonol 4-reductase/Flavanone 4-reductase; *EvGT5*, Anthocyanidin 3-O-glucosyltransferase 5; *EvRHGT1*, Anthocyanidin 5,3-O-glucosyltransferase; *EvOMT1*, Flavonoid 3'-O-methyltransferase; *EvCYP75A6*, Flavonoid 3',5'-hydroxylase.

Interestingly, specialized metabolism was the most enriched group of compounds involved in different biological processes identified in this study both at chemical and molecular levels (Figures 1C, 2C). This observation is in line with the role of specialized metabolites in adaptation to harsh environmental conditions, providing protection against several stresses (Isah, 2019). The presence of flavonoids was particularly evident. Flavonoids derive from the phenylpropanoid pathway (Figure 5A). We identified three transcripts encoding *Ev4CL* (Figure 5B), one of which was eight times more expressed in leaves than in seeds. One transcript encoding *EvCYP73A* had |FC|−2, although it only reached a *p-*value outside the established statistical criteria.

Flavonoids in leaves play diverse roles such as antioxidants, herbivore, and pathogen defense molecules, as well as excess irradiance and UV protectants (Shen et al., 2022). In seeds, the presence of flavonoids may help maintain redox control in re-hydration, increased respiration, and reserve mobilization during germination. Flavonoids have even been implicated in possibly protecting DNA against ROS-induced damage, as their location in nuclei has been established (Saslowsky et al., 2005).

Specific flavonoid pathway starts with the activity of chalcone synthase *(EvCHS*), responsible for the generation of chalcone from *p-*coumaroyl-CoA and three malonyl-CoA molecules to form the two phenyl rings (rings A and B) of the flavonoid skeleton (C6-C3-C6). The assembly of the C ring is catalyzed by chalcone isomerase (*EvCHI*), generating naringenin, which eventually leads to flavonoid subclasses (Nabavi et al., 2020). A transcript of *EvCHS* was 5-fold more expressed in leaves (down-regulated in seeds). Of the 3 DET's corresponding to *EvCHI*, one was 2 times more expressed in leaves, whereas the other two were up-regulated in seeds (Figure 5A). *EvCHI* is one of the first enzymes involved in the biosynthesis of flavones and isoflavones, besides being a key step for the biosynthesis of naringenin. The genes *EvHIDH*, and *EvIF7GT*, whose products act one step downstream of *EvCHI* and are involved in the formation of isoflavones, were up-regulated in seeds, whereas *EvIFR2* showed no change. In contrast, the *EvFNSII* transcript, encoding an enzyme associated with the production of flavone 7,4'-dihydroxyflavone one biosynthetic step ahead of *EvCHI*, was down-regulated in seeds, suggesting the participation of *EvCHI* in regulating the biosynthesis of these compounds.

Overall these data are in agreement with previously published works, in which flavones are abundant in leaves of the genus *Erythrina* (Fahmy et al., 2018; Li et al., 2021a) and different genus of the legume family are known for their high production of isoflavones in seeds, such as *Glycine max* (Tepavčević et al., 2021) and *Medicago spp* (Barreira et al., 2015). To the best of our knowledge, this is the first record of the presence of isoflavones in seeds of species of the genus *Erythrina*. This result suggests the genus could represent a potential alternative source of isoflavones. On the other hand, whereas genes of the isoflavones and flavones pathways were more enriched in seeds and leaves, respectively, when analyzed in parallel with the metabolite profile, there were compounds of these exclusive subclasses in both plant structures. This observation highlights the complex regulation of biosynthesis and accumulation of flavonoids in the species, with possible involvement of inter-organ metabolite transport mechanisms. Indeed, flavonoid transport involves several transporter types, consistent with their multiple roles in stress responses and symbiotic interactions with microorganisms (Petrussa et al., 2013). Isoflavones in seeds may be useful for the establishment of associations with N-fixing bacteria early in seedling development (Hungria et al., 2020) and may contribute to plant nutrition in the Caatinga soil.

Other common pathway leading to the formation of flavones starts from naringenin and culminates in the biosynthesis of apigenin and luteolin, which constitute the main precursors of other flavones detected in the present study. As possible transcripts involved in these steps, we identified two corresponding to flavone synthases: *EvFNSI* (no change) and *EvFNSII* (down-regulated in seeds), two *EvCYP75A6* transcripts and one *EvCYP75B1* transcript (these three displaying no change). FNS is responsible for converting flavanones such as naringenin, eriodictyol and liquiritigenin into flavones, not only apigenin and luteolin, but also 7,4′-dihydroxyflavone (Jiang et al., 2019). In *Glycine max* L. (Fabaceae) *FNSII*, classified as *CYP93B16*, directly converted a flavonone to 7,4′-dihydroxyflavone (Fliegmann et al., 2010), making FSN a candidate for this step in *E. velutina*. The catalytic activity of these proteins has been widely reported in the last decade involving not only legume species (Wu et al., 2016; Wang et al., 2020).

Transcripts encoding 2 subfamilies cytochrome P450 enzymes, CYP75A (*EvCYP75A6*) and CYP75B (*EvCYP75B1*), shared high identity with two non-Fabaceae species, *Campanula medium* (65%) and *Arabidopsis thaliana* (99%), respectively. Based on in the Swiss-Prot database, their functional domain was confirmed *in silico* as being compatible with the P450 superfamily. The CYP75A (*F3'5'H*) and CYP75B (*F3'H*) subfamilies can catalyze the conversion of apigenin to luteolin (KEGG: map0094) and perform hydroxylation of the B ring of flavonoids, important for the biosynthesis of blue and red anthocyanins (Xiao et al., 2021).

Dihydrokaempferol, also produced from naringenin, was identified only in *E. velutina* leaves. This flavonol is an important intermediate in the biosynthesis of other flavonoids. As a candidate to generate dihydrokaempferol in *E. velutina*, we selected the *EvF3H-2* transcript. *In vitro*, the corresponding enzyme catalyzes beta-hydroxylation to convert naringenin to dihydrokaempferol (Shen et al., 2009). Dihydrokaempferol can give rise to other flavonols directly (1), or indirectly from dihydroquercetin (2), or be an important precursor to generate intermediates for anthocyanidin formation (3) (Figure 5). In the direct route, products of transcripts encoding FLS (*EvFLS*) and GT6 (*EvGT6*) (both down-regulated in seeds) would be involved. In this step, the flavonol kaempferol 3-*O*-glucoside was uniquely annotated in leaves of our metabolome. FLS is involved in the formation of kaempferol, which when overexpressed in *Brassica napus*, results in accumulation of this flavonol (Vu et al., 2015), whereas GT6 glucosyltransferase is capable of performing reactions of 3-*O*-glucosides (Griesser et al., 2008). Although kaempferol was not annotated in our dataset, we identified *EvFLS* with 53% identity and a verified functional domain, suggesting that it can be a substrate for the formation of kaempferol 3-*O*-glucoside from GT6. The latter showed 95% identity with the corresponding transcript of *Fragaria ananassa*.

In the indirect path, there is initially the formation of dihydroquercetin, in which enzymes of the CYP75B superfamily participate, mainly those encoded by *F3'H*. In *Petunia hybrida*, F3'H was cloned and characterized, converting dihydrokaempferol into dihydroquercetin (Brugliera et al., 1999). It is possible that the product of *EvF3'H* transcript can take part in regulating the biosynthesis of dihydroquercetin flavonol in *E. velutina*. Subsequently, the product of the seed up-regulated transcript *EvDFR* (DFR), together with OMTs and UGTs, would lead to the formation of the other flavonols identified exclusively in seeds or the formation of leucocyanidin (step 3).

During anthocyanidin production, after formation of leucocyanidin, the consecutive steps mainly involve glycosylation and methylation reactions. All anthocyanidin class metabolites identified were glycosylated, and genes encoding sugar transfer proteins were *EvRhGT1* (up-regulated in seeds), *EvGT5* (no change) and *EvGT6* (down-regulated), in addition to *EvOMT1*, down-regulated in seeds, and showing corresponding lower abundance of its protein product. In a previous publication, we observed that leaves had more proteins with a functional UDP-glycosyltransferase domain than seeds; moreover, genes encoding enzymes putatively involved in the glycosylation of different flavonoids, including flavonols and anthocyanidins, were recorded (Chacon et al., 2021b). The overt complexity of glycosides in higher plants is seen in its numerous glycoforms, different positions and number of sugar additions (Vaistij et al., 2009).

As previously reported, the flavonol dihydrokaempferol, identified only in leaves, is an important intermediate in the biosynthesis of other flavonoids. However, different flavonols, anthocyanidins and flavanones were identified in both leaves and seeds. Perhaps at this point in the pathway there is a regulatory branch, where the possibility of an alternative route for the biosynthesis of these compounds could be considered. For example, naringenin may lead to the formation of eriodictyol, through the action of proteins such as *EvF3H*, as shown in *Chrysanthemum indicum* (Jiang et al., 2019); after the formation of eriodictyol, dihydroquercetin required for the different organs would be produced, rather than using dihydrokaempferol for this purpose (Kanehisa et al., 2022).

Protein abundance reflects a dynamic balance between different processes, from transcription to translation, and protein modification (Vogel and Marcotte, 2012). Several proteins related to primary processes (including antioxidant defense, carbon energy, and nitrogen metabolism), which are also needed to support specialized metabolism, were recorded in the present study. This observation may reflect the capacity of Caatinga plants to successfully adapt and actively assimilate carbon even under adverse conditions. This result agrees with our recent work on *Erythroxylum pungens*, another species of this peculiar semiarid biome (Chacon et al., 2021a). The presence of proteins involved in phosphate metabolism and signaling (e.g., 14-3-3, dehydrogenases, sugar phosphate transferases) may also be related to effective responses to environmental stimuli. To date, significant research has been carried out toward understanding the complete picture of cell phosphate signaling pathways. However, the regulation at the level of tissues, organs, and whole organisms is still incomplete (Crombez et al., 2019).

Numerous post-transcriptional regulatory mechanisms may contribute to a low constitutive correlation of different transcripts and their corresponding proteins (Vélez-Bermúdez and Schmidt, 2014). Despite the overt importance of gene expression analyses tools, namely RT-qPCR, several of these post-transcriptional mechanisms are not fully accounted for using these methods. As an alternative initial approach to address this problem, we applied proteome analysis to assist in the identification of transcripts that encode functional expressed products.

Proteogenomic analysis was reasonably consistent, with a positive but low match when the comparison was performed with all mRNAs with or without differential expression. Most studies of comparative analysis mRNA vs. protein, involve model plants, such as maize and *Arabidopsis* (Baerenfaller et al., 2012; Nakaminami et al., 2014; Ponnala et al., 2014; Walley et al., 2016), and few discuss regulation of biosynthesis of specialized metabolism in plants, even less so in non-model plants. Of the 27 transcripts annotated for the flavonoid biosynthesis pathway, eight had their respective proteins with significant NIAF, of which seven transcripts were regulated (four up-regulated, three down-regulated, all-in seeds) and only one transcript remained unchanged (marked by yellow dots in Figure 5A and Supplementary Figures 11, 12). Furthermore, the NIAF method is a criterion for protein quantification equivalent to the normalized spectral abundance factor (NSAF), taking into account the length of a protein during the normalization process (Zybailov et al., 2006; Carvalho et al., 2016) and producing more reproducible counts with good linearity between technical and biological replicates (McIlwain et al., 2012). These proteomic data will contribute to new exploratory search studies for genes with direct implication in the production of relevant functional products. The combined data sets herein described are being used to select constitutive reference candidate genes, design, and conduct detailed quantitative gene expression experiments under controlled environmental conditions.

This result is similar to previously published studies, in which differential mRNA expression reflects better the level of agreement with its respective protein than when compared to unregulated genes (Lan et al., 2012). This observation supports the biological significance of differential expression (Koussounadis et al., 2015), directing the selection of potential targets for further studies, in addition to highlighting the importance of quantitative and dynamic measures in understanding the changing relationship of mRNA and protein (Lee et al., 2011). Although only approximately 30% of the transcripts of the flavonoid pathway had a protein match, this condition was seen across the common pathway of phenylpropanoids, as well as in pathways that lead to the formation of different classes of flavonoids, both in leaves and seeds. Observing the data, *EvCHS, EvCHI, EVCHR, EvFLS, EvCYP75A and EvCYP75B1* could be indicated as the first target candidates for future studies of metabolic flux, since they operate directly in key sites of flavonoid biosynthesis and showed different expression profiles.

The presence of transcripts and proteins involved in flavonoid metabolism in dormant seeds can be the result of genetic and metabolic programs that took place during seed development. In addition, pre-existing transcripts (long-lived mRNAs, generally associated with monosomes) may play relevant roles during early germination, often in redox regulation (Sano et al., 2020), which may benefit from flavonoid biosynthesis related transcripts found in this study. On the other hand, it is possible that some of the proteins present in dry seeds inactivated in an oxidized state may be reduced (e.g., in disulfide bridges) and activated in early germination (Catusse et al., 2008).

In total, 394 putative transcription factors (TFs), distributed in different families, were identified in *Erythrina velutina*. An auxin response factor (ARF8) was expressed 10 times more in leaves compared to seeds. Auxin is a key regulator of plant growth and development, controlling gene expression through the ARF family (DNA-binding auxin response factors) (Li et al., 2016). Members of this family have been shown to be responsive to water stress in *soybean* (Van Ha et al., 2013), and regulate microRNAs in Fabaceae (Wang et al., 2015). ARFs were also regulated in the functional categories identified by MapMan (Supplementary Figure 10); in addition, at least four enriched gene ontologies related to transport and auxin signaling were obtained, all of which were down-regulated in seeds (Supplementary Table 6), as expected from their dormant state.

AP2/ERF family transcription factors can activate genes related to plant hormonal signaling, such as those responsive to abscisic acid (ABA) and ethylene (ET), being also involved in growth and development processes mediated by gibberellins (GAs), cytokinins (CTK) and brassinosteroids (BRs) (Xie et al., 2019). In *Arabidopsis* leaves, DEWAX2 is involved in downregulating cuticular wax biosynthesis, an important barrier protecting plants from abiotic and biotic stresses (Kim et al., 2018). Here, an AP2/ERF family transcription factor (DEWAX2) member was expressed 8 times more in seeds. This would be consistent with the need to repress cuticular wax biosynthesis in fully formed dormant seeds (with hard water, impermeable tegument). In contrast, metabolically active and growing leaves demand continuous cuticle production and restoration, particularly in the arid conditions of *E. velutina* habitat. AP2/ERF family can be the target of studies to unveil the regulation of several biological processes in *E. velutina*.

Different transcription factors that play important roles in regulating special metabolism have been identified, especially those that influence flavonoid biosynthesis. Although this class constitutes one of the best-studied metabolic pathways in plants, its genetic and molecular regulation in the genus *Erythrina* remains elusive. Various studies aimed at investigating protein complexes - MBW (MYB – bHLH–WDR) - as one of the main regulators of the flavonoid pathway. These protein complexes can provide interesting models for analyzing environmentally controlled transcript regulation (Xu et al., 2015). Furthermore, their interactions are among the best-described examples in the literature as regulators of different post-translational modifications, including dimerization, phosphorylation, protein degradation and various protein-protein interactions (Xu et al., 2015). In tomato plants and *Arabidopsis* seedlings, MYB14 and MYB111, respectively, participate in the regulation of flavonoid biosynthesis, mainly flavonol accumulation (Pandey et al., 2015; Li et al., 2021b). Although none of the MYB transcripts identified were differentially expressed, their presence in both leaves and seeds suggests that genes involved in flavonols biosynthesis were transcriptionally activated either in physiologically active leaves or during seed development and maturation, which is supported by enrichment of these compounds in the metabolic profile datasets. Interaction network analyses relating putative genes of the flavonoid pathway and MYB transcription factors reinforce this scenario.

Expression analysis also indicated transcripts encoding TFs of the bHLH family in leaves and seeds, such as MYC2. In *Arabidopsis* MYC2 regulates jasmonate (JA)-responsive pathogen defense and wound response genes, in addition to its role in positively regulating flavonoid biosynthesis (Dombrecht et al., 2007). MYC2 is also responsible for regulating genes in metabolic pathways of terpenes (Shen et al., 2016) and alkaloids (Zhang et al., 2012). Among DETs in the bHLH family, PIF4, which can regulate anthocyanin accumulation and auxin biosynthesis, appeared up-regulated in leaves (Franklin et al., 2011; Liu et al., 2021). Two down-regulated transcripts encoding FT UNE12 were also identified in seeds. This type of transcription factor is able to regulate SA accumulation in response to temperature (Bruessow et al., 2019). Two genetic ontologies related to these processes were enriched in our dataset (response to temperature stimulus [GO:0009266: up-regulated in seeds] and response to salicylic acid [GO:0009751]) (Figures 3A, 4D, respectively). Identifying TFs that affect secondary pathways of metabolism can be valuable for future metabolic engineering in organs of *Erythrina*. Modulating these regulatory proteins is one of the most effective ways of achieving high metabolic fluxes leading to target compounds (Matsuura et al., 2018).

The analysis of RNA-seq expression data in relation to proteins exclusively identified in this study is shown in Figures 4J,K. We expected the correspondence of mRNA with proteins identified exclusively in seeds and leaves to be up and down-regulated, respectively. However, two transcripts in leaves (number 1 and 2) and two in seeds (number 3 and 4) did not follow this pattern. In leaves, the heterogeneous nuclear ribonucleoprotein 1-like isoform X1 (1 and 2), a protein that modulates transcript expression, involved in post-transcriptional regulation in plants was identified (Yeap et al., 2014). In seeds, aspartate-semialdehyde dehydrogenase (3), responsible for the biosynthesis of different amino acids from aspartate, and fumarylacetoacetase (4), involved in the catabolic process of L-phenylalanine and tyrosine, were identified. Besides playing a relevant role in N and protein metabolism, these molecules originate a large array of specialized metabolites. Taken together, these results indicate strong post-transcriptional regulation. Several genes encoding proteins related to modification and degradation processes have been enriched in the dataset (Supplementary Figure 10). Future studies should focus on these aspects to advance understanding of how transcript and protein regulation is balanced to ensure adequate coordination of physiology and metabolism in plants challenged by complex natural habitats.

**Figure 6:**
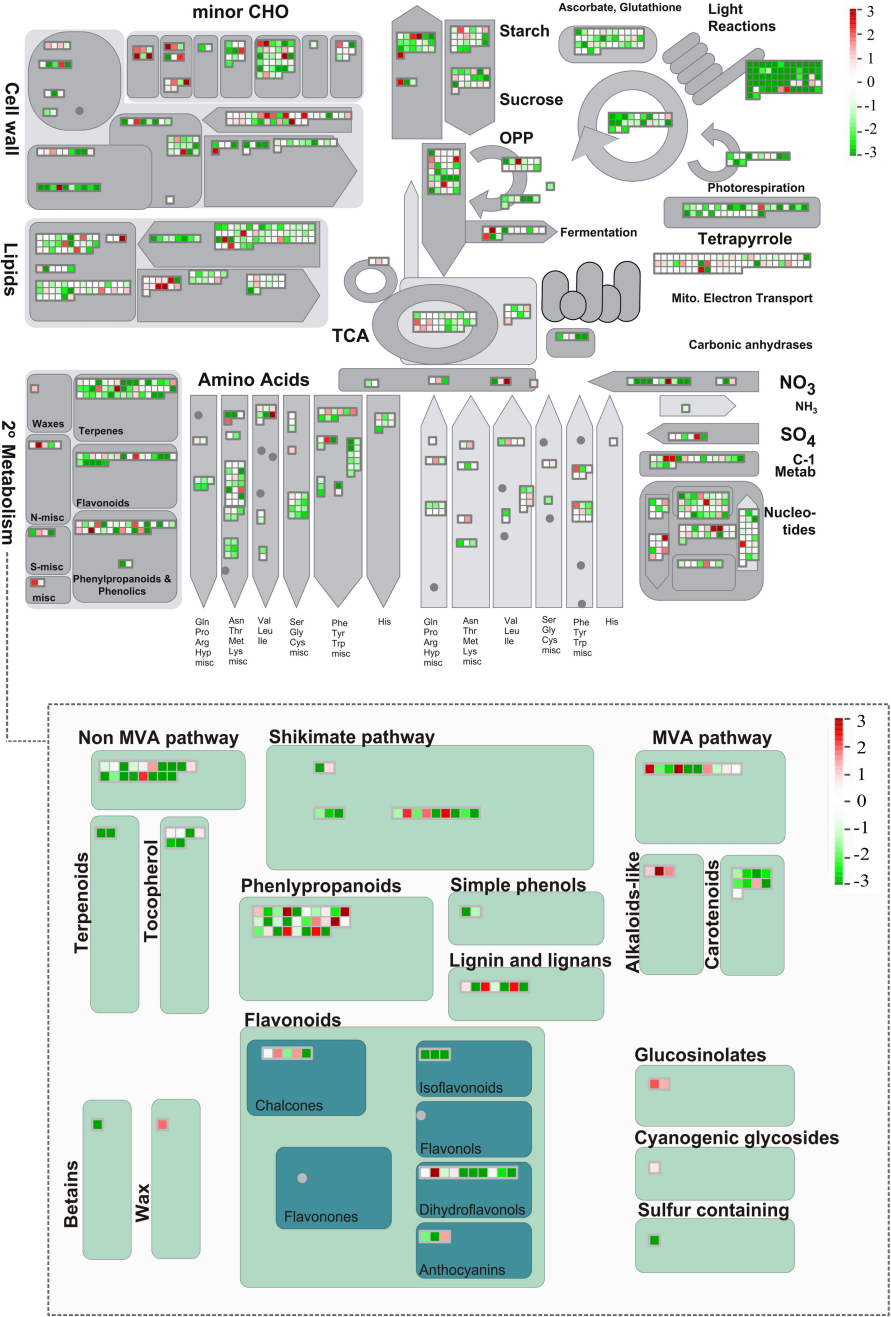
Overview of metabolic pathways of expressed transcripts performed in MapMan. Each small square represents an expressed transcript in leaves or seeds, exclusively or shared, with red color corresponding to up-regulated genes, green down-regulated transcripts, and blue unchanged transcripts. The color scale shows the fold change value in RPKM.

To sum up, exploratory and global analyses of the set of metabolites produced in leaves and seeds were carried out to better detail metabolic regulation in *Erythrina velutina*. Supported by transcriptomic data, the main genes involved in flavonoid biosynthesis, most prominent class of metabolites revealed by the data set, were identified (Figure 6). In parallel, the main biological processes, differentially expressed genes and transcription factors involved in the signaling of environmental responses and production of specialized metabolites were investigated, thereby indicating promising key targets for future studies. Data are consistent with the fact that both plant structures are effective producers and accumulators of complex and diverse specialized metabolites. Despite the observation that leaves and seeds of *E. velutina* are important potential sources of bioactive metabolites, there are still multiple standing questions regarding their regulation. Hopefully, the generated data sets herein described and analyzed will facilitate advances on current research efforts to expand the physiological, biochemical, and molecular knowledge of this environmentally resilient and medicinally important unique Brazilian semiarid tree.

## Data availability statement

Publicly available datasets were analyzed in this study. This data can be found at: NCBI PRJNA668524 and ProteomeXchange PXD031557.

## Author contributions

AR performed the botanical identification. IS performed the experimental transcriptome procedures. DC, BB, and LS analyzed and discussed the data from the transcriptome. DC and IS performed the experimental proteome procedures. DC, MS, PC, BB, WF, and MS participated in the proteome discussion and data analysis. DC and FR participated in the metabolome extraction. NL, AC, AP, DS, DC, LF, FR, and RF participated in the discussion and analysis of metabolome data. JV, CC, TA, EV, JZ, KS, and TT participated in the discussion of the manuscript. AF-N participated in the discussion of the data and helped finalize the manuscript. RG coordinated and analyzed the entire study, as well as finalized the manuscript. All authors discussed the main conclusions and contributed to writing the manuscript.

## Funding

This work was supported by the Serrapilheira Institute (grant number Serra-1709-19691), by the Ministry of Science, Technology, Innovation and Communications - MCTIC, by CNPq/National Council of Science and Technology - INCT BioNat, [grant number 465637/2014-0], Sáo Paulo Research Foundation (FAPESP grant INCTBioNat 2014/50926-0 and 2020/02207-5), and by *Coordenação de Aperfeiçoamento de Pessoal de N*í*vel Superior - Brazil* [(*CAPES*) *- Finance Code 001*).

## Conflict of interest

The authors declare that the research was conducted in the absence of any commercial or financial relationships that could be construed as a potential conflict of interest.

## Publisher's note

All claims expressed in this article are solely those of the authors and do not necessarily represent those of their affiliated organizations, or those of the publisher, the editors and the reviewers. Any product that may be evaluated in this article, or claim that may be made by its manufacturer, is not guaranteed or endorsed by the publisher.
