# Supplementary material for: Non-target molecular network and putative genes of flavonoid biosynthesis in Erythrina velutina Willd., a Brazilian semiarid native woody plant
Source: Front Plant Sci. 2022 Sep 8;13:947558. doi: 10.3389/fpls.2022.947558 (PMC9493460; doi:10.3389/fpls.2022.947558)
Supplement: Supplementary file 1 [file Data_Sheet_1.docx]

Supplementary Material

**Supplementary Table 1.** Identification and location of collection sites (Chacon et al., 2021a); The letter "p" represents pool, and its respective number indicates the collection site, with p1 to p4 representing seed samples, and p5 to p7 representing leaf samples.

| Pool set | Pools | | Harvest site | Geographical coordinates |
| --- | --- | --- | --- | --- |
|  | Seeds | Leaves |  |  |
| A | p1 | p5 | Jardim do Seridó I | 6°33'58.2"S 36°43'00.6"W |
| B | p2 | p6 | Acari I | 6°27'44.5"S 36°38'30.6"W |
| C | p3 | p7 | Acari II | 6°28'19.5"S 36°38'32.5"W |
| D | p4 | p8 | Jardim do Seridó II | 6°33'48.6"S 36°43'11.0"W |

**Supplementary Table 2.** Overview of assembly. Values indicate the number of assembled transcripts. The letter "p" represents pool, and its respective number indicates the collection site, with p1 to p4 representing seed samples, and p5 to p7 representing leaf samples.

| **Transcript number** | | | | |
| --- | --- | --- | --- | --- |
| *E. velutina*  17,822 | Seeds  30,218 | Pool 1  34,256 | P1-L1 | 69818 |
|  |  |  | P1-L2 | 68633 |
|  |  |  | P1-L3-4 | 96282 |
|  |  | Pool2  40,724 | P2-L1-2 | 114604 |
|  |  |  | P2-L3 | 84593 |
|  |  |  | P2-L4 | 84198 |
|  |  | Pool3  38,377 | P3-L1 | 64877 |
|  |  |  | P3-L2 | 63813 |
|  |  |  | P3-L3 | 64822 |
|  |  |  | P3-L4 | 64460 |
|  |  | Pool4  48,122 | P4-L1 | 74074 |
|  |  |  | P4-L2 | 73160 |
|  |  |  | P4-L3 | 74697 |
|  |  |  | P4-L4 | 73439 |
|  | Leaves  39,271 | Pool5  45,811 | P5-L1 | 84118 |
|  |  |  | P5-L2 | 83130 |
|  |  |  | P5-L3 | 84727 |
|  |  |  | P5-L4 | 83739 |
|  |  | Pool6  47,616 | P6-L1 | 86522 |
|  |  |  | P6-L2 | 85189 |
|  |  |  | P6-L3 | 86565 |
|  |  |  | P6-L4 | 86531 |
|  |  | Pool7  47,361 | P7-L1 | 84152 |
|  |  |  | P7-L2 | 82837 |
|  |  |  | P7-L3 | 84338 |
|  |  |  | P7-L4 | 84094 |
|  |  | Pool8  50,449 | P8-L1 | 90218 |
|  |  |  | P8-L2 | 90156 |
|  |  |  | P8-L3 | 91665 |
|  |  |  | P8-L4 | 91476 |

**Supplementary Table 3.** Identification of the 96 annotated metabolites. Metabolite numbers corresponds to the those cited in the main text of the article; Retention time, *m/z* value, plant organ and metabolic class are also reported.

| **Metabolite N°** | **Metabolite name** | **RT mean (min)** | ***m/z*** | **Adduct** | **Plant Structure** | **Pathway** |
| --- | --- | --- | --- | --- | --- | --- |
| 1 | [6-[3,4-dihydroxy-2,5-bis(hydroxymethyl)oxolan-2-yl]oxy-3,4,5-trihydroxyoxan-2-yl]methyl (*E*)-3-(4-hydroxy-3-methoxyphenyl)prop-2-enoate | 0.8 | 543.0557 | [M+Na]+ | Seeds | **Phenylpropanoid** |
| **** | | | | | | |
| 2 | Lactulose | 0.8 | 325.0583 | [M+H-H_2_O]^+^ | Seeds | **Carbohydrate** |
| **** | | | | | | |
| 3 | Maltotriose | 0.8 | 505.1021 | [M+H]^+^ | Seeds | **Carbohydrate** |
| **** | | | | | | |
| 4 | Isomaltulose | 1.0 | 360.0879 | [M+H-H_2_O]^+^ | Seeds | **Carbohydrate** |
| **** | | | | | | |
| 5 | Inosine | 1.0 | 268.0616 | [M+H]^+^ | Leaves,Seeds | **Other** |
| **** | | | | | | |
| 6 | Dihydrokaempferol | 1.7 | 289.0462 | [M+H]^+^ | Leaves | **Flavonols** |
| **** | | | | | | |
| 7 | (*S*)-2-Amino-3-(3-indolyl)propionic acid | 2.4 | 205.0690 | [M+H]^+^ | Leaves,Seeds | **Amino acids and analogues** |
| **** | | | | | | |
| 8 | Chalcone | 2.9 | 210.0483 | [M+H]^+^ | Seeds | **Chalcone** |
| **** | | | | | | |
| 9 | 3-Indolepropionic acid | 2.9 | 192.0364 | [M+H]^+^ | Seeds | **Amino acids and analogues** |
| **** | | | | | | |
| 10 | 2-Benzyl-1*H*-benzoimidazole | 2.9 | 210.0454 | [M+H]^+^ | Leaves,Seeds | **Other** |
| **** | | | | | | |
| 11 | (2*R*,3*R*,4*S*,5*S*,6*R*)-2-[[2-(3,4-dihydroxyphenyl)-5,7-dihydroxy-3,4-dihydro-2*H*-chromen-3-yl]oxy]-6-(hydroxymethyl)oxane-3,4,5-triol | 3.6 | 453.0698 | [M+H]^+^ | Seeds | **Flavonols*** |
| **** | | | | | | |
| 12 | Rutarin | 4.3 | 427.1265 | [M+H]^+^ | Seeds | **Coumarins and derivatives*** |
|  | | | | | | |
| 13 | 8-[(2*S*,3*R*,4*S*,5*S*,6*R*)-4,5-dihydroxy-6-(hydroxymethyl)-3-[(2*S*,3*R*,4*S*,5*R*)-3,4,5-trihydroxyoxan-2-yl]oxyoxan-2-yl]-2-(3,4-dihydroxyphenyl)-5,7-dihydroxychromen-4-one | 4.3 | 581.0766 | [M+H]^+^ | Leaves | **Flavone*** |
|  | | | | | | |
| 14 | (2*S*)-3-(4-hydroxyphenyl)-2-[[(*Z*)-3-(4-hydroxyphenyl)prop-2-enoyl]amino]propanoic acid | 4.4 | 344.0800 | [M+H-H_2_O]^+^ | Leaves | **Amino acids and analogues** |
|  | | | | | | |
| 15 | N6-Threonylcarbamoyladenosine | 4.6 | 413.0808 | [M+H]^+^ | Leaves | **Other** |
|  | | | | | | |
| 16 | Indole-3-acetyl-*L*-glutamic acid | 5.1 | 307.0421 | [M+H]^+^ | Leaves | **Amino acids and analogues** |
|  | | | | | | |
| 17 | 7,4'-Dihydroxyflavone | 5.4 | 252.0482 | [M-H]^+^ | Leaves | **Flavone*** |
|  | | | | | | |
| 18 | Scoulerine | 5.5 | 328.1030 | [M+H]^+^ | Leaves | **Other** |
|  | | | | | | |
| 19 | Flavonoid 8-*C*-glycosides | 5.7 | 611.0867 | [M+H]^+^ | Leaves | **Flavone*** |
|  | | | | | | |
| 20 | 2-Methyl-4-oxo-4*H*-pyran-3-yl 6-*O*-(4-carboxy-3-hydroxy-3-methylbutanoyl)-*β*-*D*-glucopyranoside | 5.9 | 433.0722 | [M+H]^+^ | Leaves,Seeds | **Carbohydrate** |
|  | | | | | | |
| 21 | Methoxy-quercetin-3-*O*-hexoside | 6.2 | 479.0820 | [M+H]^+^ | Seeds | **Flavonols** |
|  | | | | | | |
| 22 | Spegatrine | 6.3 | 342.1127 | [M+H-H_2_O]^+^ | Seeds | **Other** |
|  | | | | | | |
| 23 | 7-[(2*S*,3*R*,4*S*,5*S*,6*R*)-4,5-dihydroxy-6-(hydroxymethyl)-3-[(2*S*,3*R*,4*S*,5*S*,6*R*)-3,4,5-trihydroxy-6-(hydroxymethyl)oxan-2-yl]oxyoxan-2-yl]oxy-9-hydroxy-6-(4-hydroxyphenyl)-[1,3]dioxolo[4,5-g]chromen-8-one | 6.4 | 638.1627 | [M+H]^+^ | Seeds | **Flavonols*** |
|  | | | | | | |
| 24 | 2-Cyclohexen-1-one, 4-[(1*E*)-3-(beta-D-glucopyranosyloxy)-1-buten-1-yl]-4-hydroxy-3,5,5-trimethyl | 6.4 | 387.1429 | [M+H]^+^ | Leaves | **Terpenes and derivatives*** |
| **** | | | | | | |
| 25 | Indole-3-acetyl-*L*-Leucine | 6.9 | 291.0460 | [M+H]^+^ | Seeds | **Amino acids and analogues** |
|  | | | | | | |
| 26 | 2-(3,4-dihydroxyphenyl)-5,7-dihydroxy-3-[(2*S*,3*R*,4*S*,5*R*,6*R*)-3,4,5-trihydroxy-6-(hydroxymethyl)oxan-2-yl]oxychromen-4-one | 7.0 | 467.0501 | [M+H]^+^ | Seeds | **Flavonols*** |
| **** | | | | | | |
| 27 | 3-Hydroxy-1-(3-hydroxy-4-methoxyphenyl)-2-{4-[(1*E*)-3-hydroxy-1-propen-1-yl]-2-methoxyphenoxy}propyl-*β*-*D*-glucopyranoside | 7.0 | 521.1241 | [M+H-H_2_O]^+^ | Leaves | **Phenylpropanoid*** |
|  | | | | | | |
| 28 | Daidzein-8-*C*-glucoside | 7.2 | 417.0576 | [M+H]^+^ | Leaves,Seeds | **Isoflavone*** |
|  | | | | | | |
| 29 | 3,5,7,3',4'-Pentahydroxyflavanone (dihydrotricetin) | 7.6 | 305.0143 | [M+H]^+^ | Seeds | **Flavonone** |
| **** | | | | | | |
| 30 | 5,7-dihydroxy-2-(4-hydroxyphenyl)-6,8-bis[3,4,5-trihydroxy-6-(hydroxymethyl)oxan-2-yl]chromen-4-one | 8.1 | 595.0928 | [M+H]^+^ | Leaves,Seeds | **Flavone*** |
|  | | | | | | |
| 31 | Daidzin | 8.1 | 417.0611 | [M+H]^+^ | Leaves,Seeds | **Isoflavone*** |
|  | | | | | | |
| 32 | Daidzin | 8.2 | 417.0529 | [M+H]^+^ | Seeds | **Isoflavone*** |
|  | | | | | | |
| 33 | 5,7-dihydroxy-2-(4-hydroxyphenyl)-8-[3,4,5-trihydroxy-6-(hydroxymethyl)oxan-2-yl]-6-(3,4,5-trihydroxyoxan-2-yl)chromen-4-one | 8.4 | 565.0824 | [M+H]^+^ | Leaves,Seeds | **Flavone*** |
|  | | | | | | |
| 34 | 2-[4-[3-[3,4-dihydroxy-4-(hydroxymethyl)oxolan-2-yl]oxy-4,5-dihydroxy-6-(hydroxymethyl)oxan-2-yl]oxyphenyl]-7-hydroxy-2,3-dihydrochromen-4-one | 8.6 | 549.0849 | [M-H]^+^ | Seeds | **Flavone*** |
|  | | | | | | |
| 35 | Luteolin-6-*C*-glucoside | 8.6 | 449.0447 | [M+H]^+^ | Leaves | **Flavone*** |
|  | | | | | | |
| 36 | 2,4,6-trihydroxy-2-[(4-hydroxyphenyl)methyl]-1-benzofuran-3-one | 8.7 | 289.0239 | [M+H]^+^ | Seeds | **Aurones** |
|  | | | | | | |
| 37 | Maritimetin-6-*O*-glucoside | 8.7 | 451.0553 | [M+H]^+^ | Seeds | **Aurones** |
|  | | | | | | |
| 38 | Arginine conjugated cholic acid | 8.8 | 1129.1947 | [2M+H]^+^ | Leaves | **Amino acids and analogues** |
|  | | | | | | |
| 39 | Flavone base + 3*O*, *O*-HexA-HexA | 9.6 | 623.0500 | [M+H]^+^ | Leaves | **Flavone*** |
|  | | | | | | |
| 40 | Apigenin-8-*C*-glucoside | 9.8 | 433.0521 | [M+H]^+^ | Leaves,Seeds | **Flavone*** |
|  | | | | | | |
| 41 | 3-(4-hydroxyphenyl)-7-methoxy-5-[(3*R*,4*S*,5*S*,6*R*)-3,4,5-trihydroxy-6-(hydroxymethyl)oxan-2-yl]oxychromen-4-one | 10.5 | 449.0499 | [M+H]^+^ | Leaves | **Isoflavone*** |
|  | | | | | | |
| 42 | Astragalin | 10.6 | 449.0466 | [M+H]^+^ | Leaves | **Flavonols*** |
|  | | | | | | |
| 43 | (2*S*,3*S*,4*S*,5*R*,6*S*)-6-[2-(3,4-dihydroxyphenyl)-5-hydroxy-4-oxochromen-7-yl]oxy-3,4,5-trihydroxyoxane-2-carboxylic acid | 10.7 | 463.0235 | [M+H]^+^ | Leaves | **Flavone*** |
|  | | | | | | |
| 44 | 5,7-dihydroxy-2-(4-hydroxyphenyl)-6,8-bis(3,4,5-trihydroxyoxan-2-yl)chromen-4-one | 10.7 | 535.1528 | [M+H]^+^ | Leaves | **Flavone*** |
|  | | | | | | |
| 45 | 5,7-dihydroxy-2-(4-hydroxyphenyl)-6-[(2*S*,3*R*,4*R*,5*S*,6*R*)-3,4,5-trihydroxy-6-(hydroxymethyl)oxan-2-yl]-8-[(2*S*,3*R*,4*R*,5*R*,6*S*)-3,4,5-trihydroxy-6-methyloxan-2-yl]chromen-4-one | 10.8 | 579.0968 | [M+H]^+^ | Leaves,Seeds | **Flavone*** |
|  | | | | | | |
| 46 | 3'-*O*-Methylluteolin 6-*C*-glucoside (isoscoparine) | 10.9 | 463.0581 | [M+H]^+^ | Leaves | **Flavone*** |
|  | | | | | | |
| 47 | Flavonoid 8-*C*-glycosides | 11.0 | 564.2861 | [M+H]^+^ | Leaves | **Flavone*** |
|  | | | | | | |
| 48 | Flavonoid 8-*C*-glycosides | 11.0 | 564.2851 | [M+H]^+^ | Leaves | **Flavone*** |
|  | | | | | | |
| 49 | Petunidin-3-*O*-*β*-glucoside | 11.1 | 479.0471 | [M+H]^+^ | Seeds | **Anthocyanin*** |
|  | | | | | | |
| 50 | Isoflavone base + 2*O*, *O*-MalonylHex | 11.7 | 503.0565 | [M+H]^+^ | Leaves,Seeds | **Isoflavone*** |
|  | | | | | | |
| 51 | Luteolin-4'-*O*-glucoside | 12.2 | 449.0401 | [M+H]^+^ | Leaves,Seeds | **Flavone*** |
|  | | | | | | |
| 52 | Apigenin-7-*O*-glucoside | 12.2 | 433.0547 | [M+H]^+^ | Leaves,Seeds | **Flavone*** |
|  | | | | | | |
| 53 | (2*S*,3*S*,4*S*,5*R*,6*S*)-3,4,5-trihydroxy-6-[5-hydroxy-2-(4-hydroxyphenyl)-4-oxochromen-7-yl]oxyoxane-2-carboxylic acid | 12.5 | 447.0307 | [M+H]^+^ | Leaves | **Flavone*** |
|  | | | | | | |
| 54 | 5-[6-[[3,4-dihydroxy-4-(hydroxymethyl)oxolan-2-yl]oxymethyl]-3,4,5-trihydroxyoxan-2-yl]oxy-4-(3,4-dihydroxyphenyl)-7-methoxychromen-2-one | 12.8 | 595.1352 | [M-H]^+^ | Seeds | **Coumarins and derivatives*** |
|  | | | | | | |
| 55 | Neodiosmin | 12.8 | 609.0992 | [M+H]^+^ | Seeds | **Flavone*** |
|  | | | | | | |
| 56 | 4-(3,4-dihydroxyphenyl)-7-methoxy-5-[(2*S*,3*R*,4*S*,5*S*,6*R*)-3,4,5-trihydroxy-6-(hydroxymethyl)oxan-2-yl]oxychromen-2-one | 13.0 | 925.1375 | [2M+H]^+^ | Seeds | **Coumarins and derivatives*** |
|  | | | | | | |
| 57 | Peonidin 3-galactoside | 13.0 | 463.0567 | [M+H]^+^ | Leaves,Seeds | **Anthocyanin*** |
|  | | | | | | |
| 58 | (2*S*,3*S*,4*S*,5*R*,6*S*)-3,4,5-trihydroxy-6-[5-hydroxy-2-(4-hydroxyphenyl)-6-methoxy-4-oxochromen-7-yl]oxyoxane-2-carboxylic acid | 13.0 | 477.0360 | [M+H]^+^ | Leaves | **Flavone*** |
|  | | | | | | |
| 59 | Anthocyanidin base + 4*O*, 2MeO, *O*-Hex | 13.3 | 493.0591 | [M+H]^+^ | Seeds | **Anthocyanin*** |
|  | | | | | | |
| 60 | 6''-*O*-Acetyldaidzin | 13.3 | 459.0583 | [M+H]^+^ | Seeds | **Isoflavone*** |
|  | | | | | | |
| 61 | Isoflavone base + 3*O*, *O*-MalonylHex | 15.0 | 519.0493 | [M+H]^+^ | Leaves,Seeds | **Isoflavone** |
|  | | | | | | |
| 62 | Isoflavone base + 1*O*, 2MeO, *O*-MalonylHex | 15.5 | 549.0547 | [M+H]^+^ | Leaves,Seeds | **Isoflavone** |
|  | | | | | | |
| 63 | Luteolin | 15.75 | 287.0097 | [M+H]^+^ | Leaves,Seeds | **Flavone** |
|  | | | | | | |
| 64 | *N*-pyridin-3-yl-2-[(4,8,8-trimethyl-2-oxo-9,10-dihydropyrano[2,3-*H*]chromen-5-yl)oxy]acetamide | 18.5 | 394.1665 | [M+H]^+^ | Leaves,Seeds | **Coumarins and derivatives** |
|  | | | | | | |
| 65 | Diosmetin | 19.0 | 301.0185 | [M+H]^+^ | Leaves,Seeds | **Flavone** |
|  | | | | | | |
| 66 | 5,7-dihydroxy-2-(4-hydroxy-3-methoxyphenyl)chromen-4-one | 19.0 | 301.0202 | [M+H]^+^ | Seeds | **Flavone** |
|  | | | | | | |
| 67 | 5,7-dihydroxy-2-(4-hydroxy-3-methoxyphenyl)chromen-4-one | 19.1 | 301.0216 | [M+H]^+^ | Leaves,Seeds | **Flavone** |
|  | | | | | | |
| 68 | 5-HEPE | 24.3 | 318.2486 | [M-H]^+^ | Leaves,Seeds | **Fatty acids** |
|  | | | | | | |
| 69 | Nobiletin | 25.5 | 403.0775 | [M+H]^+^ | Leaves | **Flavone** |
|  | | | | | | |
| 70 | Soyasaponin I | 26.4 | 943.4270 | [M+H]^+^ | Leaves,Seeds | **Saponins*** |
|  | | | | | | |
| 71 | Phytosphingosine | 27.6 | 318.2509 | [M+H]^+^ | Leaves,Seeds | **Fatty acids** |
|  | | | | | | |
| 72 | Phytosphingosine | 27.6 | 318.2455 | [M+H]^+^ | Leaves,Seeds | **Fatty acids** |
|  | | | | | | |
| 73 | Soyasapogenol B | 28.0 | 1069.4543 | [M+H]^+^ | Leaves | **Saponins*** |
|  | | | | | | |
| 74 | 7b,9-Dihydroxy-3-(hydroxymethyl)-1,1,6,8-tetramethyl-5-oxo-1,1a,1b,4,4a,5,7a,7b,8,9-decahydro-9a*H*-cyclopropa[3,4]benzo[1,2-e]azulen-9a-yl acetate | 28.4 | 432.1763 | [M+ACN+H]^+^ | Leaves,Seeds | **Terpenes and derivatives** |
| **** | | | | | | |
| 75 | Flavin adenine dinucleotide | 28.5 | 783.3044 | [M+H]^+^ | Leaves | **Other** |
| **** | | | | | | |
| 76 | 6-[3-[(3,4-dimethoxyphenyl)methyl]-4-methoxy-2-(methoxymethyl)butyl]-4-methoxy-1,3-benzodioxole | 28.5 | 415.1527 | [M+H-H_2_O]^+^ | Leaves,Seeds | **Lignans** |
| **** | | | | | | |
| 77 | 6-[3-[(3,4-dimethoxyphenyl)methyl]-4-methoxy-2-(methoxymethyl)butyl]-4-methoxy-1,3-benzodioxole | 29.2 | 415.1541 | [M+H-H_2_O]^+^ | Leaves,Seeds | **Lignans** |
| **** | | | | | | |
| 78 | Monogalactosylmonoacylglycerol | 29.2 | 515.2530 | [M+H]^+^ | Leaves | **Glycerolipids** |
| **** | | | | | | |
| 79 | 9,12,15-Octadecatrienoic acid, 3-(hexopyranosyloxy)-2-hydroxypropyl ester, (9*Z*,12*Z*,15*Z*) | 30.2 | 532.2756 | [M+H-H_2_O]^+^ | Leaves | **Fatty acids** |
| **** | | | | | | |
| 80 | 1-palmitoyl-2-hydroxy-sn-glycero-3-phosphoethanolamine | 30.8 | 454.2317 | [M+H]^+^ | Leaves,Seeds | **Fatty acids** |
| **** | | | | | | |
| 81 | Palmitoyllysolectithin | 31.1 | 496.2691 | [M+H]^+^ | Leaves,Seeds | **Fatty acids** |
| **** | | | | | | |
| 82 | 1-Hexadecanoyl-sn-glycero-3-phosphocholine | 31.1 | 518.2458 | [M+Na]^+^ | Seeds | **Fatty acids** |
| **** | | | | | | |
| 83 | 1-(9*Z*-Octadecenoyl)-sn-glycero-3-phosphoethanolamine | 31.4 | 502.2171 | [M+Na]^+^ | Seeds | **Fatty acids** |
| **** | | | | | | |
| 84 | (3*R*,6*R*)-6-((3*R*,5*S*,7*R*,8*R*,9*S*,10*S*,12*S*,13*R*,14*S*,17*R*)-3,7-diacetoxy-12-hydroxy-10,13-dimethylhexadecahydro-1*H*-cyclopenta[a]phenanthren-17-yl)heptane-1,3-diyl diacetate | 33.5 | 628.2429 | [M+Na]^+^ | Leaves | **Terpenes and derivatives** |
| **** | | | | | | |
| 85 | 13-Keto-9*Z*,11*E*-octadecadienoic acid | 34.7 | 279.1871 | [M+H-H_2_O]^+^ | Leaves,Seeds | **Fatty acids** |
| **** | | | | | | |
| 86 | 1-Hexadecanoyl-sn-glycerol | 35.5 | 331.2307 | [M+H]^+^ | Leaves,Seeds | **Fatty acids** |
| **** | | | | | | |
| 87 | 1-Hexadecanoyl-sn-glycerol | 35.5 | 683.4620 | [2M+Na]^+^ | Leaves,Seeds | **Fatty acids** |
| **** | | | | | | |
| 88 | Monoolein | 36.01 | 357.2402 | [M+H]^+^ | Seeds | **Fatty acids** |
| **** | | | | | | |
| 89 | 4,9-dimethoxy-7-methylfuro[3,2-g]chromen-5-one | 36.7 | 802.4940 | [3M+Na]^+^ | Leaves,Seeds | **Coumarins and derivatives** |
| **** | | | | | | |
| 90 | 1,2-Dioleoyl-sn-glycero-3-phosphoethanolamine-*N*-methyl | 36.8 | 758.4755 | [M-H]^+^ | Leaves,Seeds | **Fatty acids** |
| **** | | | | | | |
| 91 | (*E*)-3-(3,4-dimethoxyphenyl)-1-(2,4,6-trimethoxyphenyl)prop-2-en-1-one | 37.3 | 740.4653 | [2M+Na]^+^ | Leaves,Seeds | **Chalcone** |
| **** | | | | | | |
| 92 | Glycerol 1-stearate | 37.7 | 359.2607 | [M+H]^+^ | Leaves,Seeds | **Fatty acids** |
| **** | | | | | | |
| 93 | 5-(1,2,4a,5-tetramethyl-7-oxo-3,4,8,8a-tetrahydro-2*H*-naphthalen-1-yl)-3-methylpentanoic acid | 38.1 | 336.2729 | [M+H-H_2_O]^+^ | Leaves,Seeds | **Terpenes and derivatives** |
| **** | | | | | | |
| 94 | 13-Docosenamide | 39.6 | 675.5957 | [2M+H]^+^ | Leaves,Seeds | **Fatty acids** |
| **** | | | | | | |
| 95 | (2*S*)-1-*O*-(9*Z*,12*Z*,15*Z*-octadecatrienoyl)-2-*O*-(6*Z*,9*Z*,12*Z*,15*Z*-octa-decatetranoyl)-3-*β*-*D*-galacto-pyranosyl-sn-glycerol | 42.1 | 796.5028 | [M+Na+H]^+^ | Leaves | **Fatty acids** |
| **** | | | | | | |
| 96 | Putative Monogalactosyl Diacylglycerol (MGDG) | 43.1 | 772.5058 | [M+H-H_2_O]^+^ | Leaves | **Glycerolipids** |
| **** | | | | | | |

**Supplementary Table 4.** Metrics of isolated and combined leaf and seed assembly

| \| *Assembly leaves and seeds 17822 transcripts* \|  \| \| --- \| --- \| \| sum = 26804694, n = 17822, ave = 1504.02, largest = 14409 \|  \| \| N50 = 1882, n = 4928 \|  \| \| N60 = 1653, n = 6446 \|  \| \| N70 = 1410, n = 8200 \|  \| \| N80 = 1172, n = 10278 \|  \| \| N90 = 880, n = 12892 \|  \| \| N100 = 202, n = 17822 \|  \| \| N_count = 0 \|  \| \| Gaps = 0 \|  \| |
| --- | --- | --- | --- | --- | --- | --- | --- | --- | --- | --- | --- | --- | --- | --- | --- | --- | --- | --- | --- | --- |
| \| *Assembly only seeds 30271 transcripts* \| \| --- \| \| sum = 36376974, n = 30218, ave = 1203.82, largest = 10091 \| \| N50 = 1606, n = 7682 \| \| N60 = 1374, n = 10134 \| \| N70 = 1149, n = 13021 \| \| N80 = 912, n = 16560 \| \| N90 = 636, n = 21281 \| \| N100 = 201, n = 30218 \| \| N_count = 0 \| \| Gaps = 0 \| |
| \| *Assembly only leaves 39271 transcripts* \| \| --- \| \| sum = 47985888, n = 39271, ave = 1221.92, largest = 13779 \| \| N50 = 1652, n = 9777 \| \| N60 = 1410, n = 12920 \| \| N70 = 1177, n = 16634 \| \| N80 = 930, n = 21203 \| \| N90 = 635, n = 27375 \| \| N100 = 201, n = 39271 \| \| N_count = 0 \| Gaps = 0 \| |

**Supplementary Table 5.** Major metabolic pathways identified by the Kyoto Encyclopedia of Genes and Genomes (KEGG)

| **Pathway** | **Transcript number** |
| --- | --- |
| Biosynthesis of secondary metabolites | 381 |
| Microbial metabolism in diverse environments | 138 |
| Biosynthesis of cofactors | 122 |
| Ribosome | 108 |
| Biosynthesis of amino acids | 94 |
| RNA transport | 93 |
| Carbon metabolism | 85 |
| Spliceosome | 77 |
| Protein processing in endoplasmic reticulum | 71 |
| Oxidative phosphorylation | 63 |
| Endocytosis | 55 |
| Ubiquitin mediated proteolysis | 53 |
| Purine metabolism | 44 |
| Cell cycle | 42 |
| Cysteine and methionine metabolism | 42 |
| Amino sugar and nucleotide sugar metabolism | 37 |
| Plant hormone signal transduction | 36 |
| MAPK signaling pathway - plant | 34 |

**Supplementary Table 6.** Analysis of GO enrichment of differentially expressed and up-regulated transcripts

| **GO.ID** | **p-value** | **Term** |
| --- | --- | --- |
| GO:0000309 | 0.00029 | nicotinamide-nucleotide adenylyltransferase activity |
| GO:0000902 | 0.0078 | cell morphogenesis |
| GO:0000904 | 0.00478 | cell morphogenesis involved in differentiation |
| GO:0000932 | 0.0093 | cytoplasmic mRNA processing body |
| GO:0000956 | 0.00868 | nuclear-transcribed mRNA catabolic process |
| GO:0001101 | 2.4e-08 | response to acid chemical |
| GO:0002229 | 0.00604 | defense response to oomycetes |
| GO:0002239 | 0.00685 | response to oomycetes |
| GO:0003006 | 0.00054 | developmental process involved in reproduction |
| GO:0003676 | 0.00055 | nucleic acid binding |
| GO:0003723 | 0.00865 | RNA binding |
| GO:0004515 | 0.00029 | nicotinate-nucleotide adenylyltransferase activity |
| GO:0004527 | 0.00997 | exonuclease activity |
| GO:0004557 | 0.0023 | alpha-galactosidase activity |
| GO:0004730 | 0.00277 | pseudouridylate synthase activity |
| GO:0005342 | 0.00491 | organic acid transmembrane transporter activity |
| GO:0005488 | 0.00043 | binding |
| GO:0005622 | 4.4e-05 | intracellular |
| GO:0005623 | 0.00007 | cell |
| GO:0005634 | 0.00001 | nucleus |
| GO:0005667 | 0.0045 | transcription factor complex |
| GO:0005996 | 0.00192 | monosaccharide metabolic process |
| GO:0006012 | 0.00336 | galactose metabolic process |
| GO:0006139 | 0.00019 | nucleobase-containing compound metabolic process |
| GO:0006396 | 0.00909 | RNA processing |
| GO:0006401 | 0.00477 | RNA catabolic process |
| GO:0006402 | 0.00164 | mRNA catabolic process |
| GO:0006473 | 0.0098 | protein acetylation |
| GO:0006475 | 0.00912 | internal protein amino acid acetylation |
| GO:0006725 | 0.00062 | cellular aromatic compound metabolic process |
| GO:0006807 | 0.00376 | nitrogen compound metabolic process |
| GO:0006820 | 0.00812 | anion transport |
| GO:0006865 | 0.00912 | amino acid transport |
| GO:0006950 | 0.00018 | response to stress |
| GO:0006970 | 0.0017 | response to osmotic stress |
| GO:0007275 | 1.8e-05 | multicellular organismal development |
| GO:0008353 | 0.00153 | RNA polymerase II carboxy-terminal domain kinase activity |
| GO:0009065 | 0.00581 | glutamine family amino acid catabolic process |
| GO:0009266 | 0.00073 | response to temperature stimulus |
| GO:0009314 | 0.00619 | response to radiation |
| GO:0009408 | 0.00022 | response to heat |
| GO:0009411 | 0.00252 | response to UV |
| GO:0009414 | 6.6e-05 | response to water deprivation |
| GO:0009415 | 5.6e-06 | response to water |
| GO:0009416 | 0.00464 | response to light stimulus |
| GO:0009435 | 0.00158 | NAD biosynthetic process |
| GO:0009555 | 0.00782 | pollen development |
| GO:0009628 | 1.5e-09 | response to abiotic stimulus |
| GO:0009650 | 0.0042 | UV protection |
| GO:0009651 | 0.00413 | response to salt stress |
| GO:0009719 | 0.00057 | response to endogenous stimulus |
| GO:0009725 | 0.00169 | response to hormone |
| GO:0009737 | 0.00037 | response to abscisic acid |
| GO:0009739 | 0.0048 | response to gibberellin |
| GO:0009791 | 0.00015 | post-embryonic development |
| GO:0009826 | 0.00919 | unidimensional cell growth |
| GO:0009845 | 0.00789 | seed germination |
| GO:0009860 | 0.00744 | pollen tube growth |
| GO:0010029 | 0.00148 | regulation of seed germination |
| GO:0010033 | 0.0002 | response to organic substance |
| GO:0010035 | 4.6e-05 | response to inorganic substance |
| GO:0010152 | 0.00237 | pollen maturation |
| GO:0010154 | 0.0021 | fruit development |
| GO:0010158 | 0.00766 | abaxial cell fate specification |
| GO:0010187 | 0.00158 | negative regulation of seed germination |
| GO:0010468 | 0.00975 | regulation of gene expression |
| GO:0010629 | 0.00849 | negative regulation of gene expression |
| GO:0015171 | 0.00648 | amino acid transmembrane transporter activity |
| GO:0015711 | 0.00284 | organic anion transport |
| GO:0015849 | 0.00388 | organic acid transport |
| GO:0016070 | 0.00068 | RNA metabolic process |
| GO:0016442 | 0.0023 | RISC complex |
| GO:0016573 | 0.00912 | histone acetylation |
| GO:0016574 | 0.0042 | histone ubiquitination |
| GO:0018393 | 0.00912 | internal peptidyl-lysine acetylation |
| GO:0018394 | 0.00912 | peptidyl-lysine acetylation |
| GO:0019219 | 0.00727 | regulation of nucleobase-containing compound metabolic process |
| GO:0019318 | 0.00478 | hexose metabolic process |
| GO:0019359 | 0.00604 | nicotinamide nucleotide biosynthetic process |
| GO:0019363 | 0.00685 | pyridine nucleotide biosynthetic process |
| GO:0019438 | 0.0069 | aromatic compound biosynthetic process |
| GO:0019674 | 0.00868 | NAD metabolic process |
| GO:0022414 | 0.00758 | reproductive process |
| GO:0031047 | 0.0056 | gene silencing by RNA |
| GO:0031323 | 0.00736 | regulation of cellular metabolic process |
| GO:0031332 | 0.0023 | RNAi effector complex |
| GO:0032501 | 0.00013 | multicellular organismal process |
| GO:0032502 | 9.6e-06 | developmental process |
| GO:0033523 | 0.0042 | histone H2B ubiquitination |
| GO:0033993 | 0.0002 | response to lipid |
| GO:0034389 | 0.00017 | lipid particle organization |
| GO:0034613 | 0.00368 | cellular protein localization |
| GO:0034641 | 0.00773 | cellular nitrogen compound metabolic process |
| GO:0034654 | 0.00554 | nucleobase-containing compound biosynthetic process |
| GO:0034655 | 0.00448 | nucleobase-containing compound catabolic process |
| GO:0042221 | 8.9e-07 | response to chemical |
| GO:0043226 | 1 | organelle |
| GO:0043227 | 2.9e-05 | membrane-bounded organelle |
| GO:0043229 | 0.0014 | intracellular organelle |
| GO:0043231 | 2.8e-05 | intracellular membrane-bounded organelle |
| GO:0043754 | 2 | dihydrolipoyllysine-residue |
| GO:0044265 | 0.00464 | cellular macromolecule catabolic process |
| GO:0044424 | 0.0003 | intracellular part |
| GO:0044464 | 6.3e-05 | cell part |
| GO:0044699 | 0.00043 | single-organism process |
| GO:0044702 | 0.00491 | single organism reproductive process |
| GO:0044707 | 3.2e-05 | single-multicellular organism process |
| GO:0044767 | 2.1e-05 | single-organism developmental process |
| GO:0045184 | 0.00641 | establishment of protein localization |
| GO:0046483 | 0.00138 | heterocycle metabolic process |
| GO:0046700 | 0.00274 | heterocycle catabolic process |
| GO:0046942 | 0.00388 | carboxylic acid transport |
| GO:0046943 | 0.00491 | carboxylic acid transmembrane transporter activity |
| GO:0048316 | 0.00335 | seed development |
| GO:0048580 | 0.00142 | regulation of post-embryonic development |
| GO:0048589 | 0.00287 | developmental growth |
| GO:0048608 | 0.00133 | reproductive structure development |
| GO:0048731 | 0.00134 | system development |
| GO:0048856 | 0.00009 | anatomical structure development |
| GO:0048868 | 0.00753 | pollen tube development |
| GO:0050789 | 0.00031 | regulation of biological process |
| GO:0050794 | 0.00286 | regulation of cellular process |
| GO:0050896 | 4.9e-06 | response to stimulus |
| GO:0051171 | 0.00626 | regulation of nitrogen compound metabolic process |
| GO:0051239 | 0.00757 | regulation of multicellular organismal process |
| GO:0051536 | 0.00443 | iron-sulfur cluster binding |
| GO:0051539 | 0.00548 | 4 iron, 4 sulfur cluster binding |
| GO:0051540 | 0.00443 | metal cluster binding |
| GO:0052692 | 0.0023 | raffinose alpha-galactosidase activity |
| GO:0061458 | 0.00133 | reproductive system development |
| GO:0065007 | 0.00023 | biological regulation |
| GO:0070566 | 0.00117 | adenylyltransferase activity |
| GO:0070727 | 0.00516 | cellular macromolecule localization |
| GO:0080090 | 0.00693 | regulation of primary metabolic process |
| GO:0090304 | 0.0003 | nucleic acid metabolic process |
| GO:0090351 | 0.00336 | seedling development |
| GO:0097159 | 0.00932 | organic cyclic compound binding |
| GO:0097305 | 0.00145 | response to alcohol |
| GO:1900140 | 0.00177 | regulation of seedling development |
| GO:1901360 | 0.00019 | organic cyclic compound metabolic process |
| GO:1901361 | 0.00888 | organic cyclic compound catabolic process |
| GO:1901362 | 0.00713 | organic cyclic compound biosynthetic process |
| GO:1901363 | 0.00924 | heterocyclic compound binding |
| GO:1901700 | 1.2e-08 | response to oxygen-containing compound |

**Supplementary Table 7.** Analysis of GO enrichment of differentially expressed and down-regulated transcripts

| **GO.ID** | **p-value** | **Term** |
| --- | --- | --- |
| GO:0009507 | 1.00E-30 | chloroplast |
| GO:0009536 | 1.00E-30 | plastid |
| GO:0044434 | 1.00E-30 | chloroplast part |
| GO:0044435 | 1.00E-30 | plastid part |
| GO:0044444 | 1.00E-30 | cytoplasmic part |
| GO:0005737 | 1.00E-30 | cytoplasm |
| GO:0044424 | 1.00E-30 | intracellular part |
| GO:0043231 | 1.00E-30 | intracellular membrane-bounded organelle |
| GO:0043227 | 1.00E-30 | membrane-bounded organelle |
| GO:0005622 | 1.00E-30 | intracellular |
| GO:0009570 | 1.00E-30 | chloroplast stroma |
| GO:0009532 | 1.00E-30 | plastid stroma |
| GO:0043229 | 1.00E-30 | intracellular organelle |
| GO:0043226 | 1.00E-30 | organelle |
| GO:0044464 | 1.00E-30 | cell part |
| GO:0009579 | 1.00E-30 | thylakoid |
| GO:0005623 | 1.00E-30 | cell |
| GO:0009526 | 1.00E-30 | plastid envelope |
| GO:0009941 | 1.00E-30 | chloroplast envelope |
| GO:0009534 | 1.00E-30 | chloroplast thylakoid |
| GO:0031976 | 1.00E-30 | plastid thylakoid |
| GO:0044446 | 1.00E-30 | intracellular organelle part |
| GO:0044422 | 1.00E-30 | organelle part |
| GO:0031984 | 1.00E-30 | organelle subcompartment |
| GO:0044436 | 1.00E-30 | thylakoid part |
| GO:0055035 | 1.00E-30 | plastid thylakoid membrane |
| GO:0042651 | 1.00E-30 | thylakoid membrane |
| GO:0009535 | 1.00E-30 | chloroplast thylakoid membrane |
| GO:0034357 | 1.00E-30 | photosynthetic membrane |
| GO:0031967 | 1.00E-30 | organelle envelope |
| GO:0031975 | 1.00E-30 | envelope |
| GO:0009657 | 3.80E-23 | plastid organization |
| GO:0015979 | 6.20E-17 | photosynthesis |
| GO:1901564 | 1.40E-15 | organonitrogen compound metabolic process |
| GO:0009658 | 4.80E-14 | chloroplast organization |
| GO:0044281 | 3.70E-13 | small molecule metabolic process |
| GO:0019684 | 1.30E-12 | photosynthesis, light reaction |
| GO:0009735 | 1.50E-12 | response to cytokinin |
| GO:0009987 | 4.90E-12 | cellular process |
| GO:0044763 | 8.30E-12 | single-organism cellular process |
| GO:0009628 | 1.10E-11 | response to abiotic stimulus |
| GO:0006807 | 1.70E-11 | nitrogen compound metabolic process |
| GO:0044711 | 3.40E-11 | single-organism biosynthetic process |
| GO:1901566 | 5.40E-11 | organonitrogen compound biosynthetic process |
| GO:0031977 | 1.00E-10 | thylakoid lumen |
| GO:0006996 | 1.50E-10 | organelle organization |
| GO:0006520 | 5.30E-10 | cellular amino acid metabolic process |
| GO:0044237 | 6.10E-10 | cellular metabolic process |
| GO:0006091 | 6.90E-10 | generation of precursor metabolites and energy |
| GO:0071840 | 1.90E-09 | cellular component organization or biogenesis |
| GO:0050896 | 2.70E-09 | response to stimulus |
| GO:0016853 | 2.80E-09 | isomerase activity |
| GO:0016043 | 5.90E-09 | cellular component organization |
| GO:0051186 | 6.40E-09 | cofactor metabolic process |
| GO:0006950 | 6.80E-09 | response to stress |
| GO:0019752 | 1.10E-08 | carboxylic acid metabolic process |
| GO:0009543 | 1.10E-08 | chloroplast thylakoid lumen |
| GO:0031978 | 1.10E-08 | plastid thylakoid lumen |
| GO:0043436 | 1.80E-08 | oxoacid metabolic process |
| GO:0006082 | 2.00E-08 | organic acid metabolic process |
| GO:0009409 | 2.20E-08 | response to cold |
| GO:0009266 | 3.50E-08 | response to temperature stimulus |
| GO:0044249 | 3.70E-08 | cellular biosynthetic process |
| GO:0009058 | 4.00E-08 | biosynthetic process |
| GO:0034641 | 6.50E-08 | cellular nitrogen compound metabolic process |
| GO:1901576 | 8.20E-08 | organic substance biosynthetic process |
| GO:0009605 | 1.00E-07 | response to external stimulus |
| GO:0006457 | 2.10E-07 | protein folding |
| GO:0071822 | 3.40E-07 | protein complex subunit organization |
| GO:0051188 | 5.20E-07 | cofactor biosynthetic process |
| GO:0042221 | 7.10E-07 | response to chemical |
| GO:1901605 | 9.80E-07 | alpha-amino acid metabolic process |
| GO:0032502 | 9.90E-07 | developmental process |
| GO:0044283 | 1.80E-06 | small molecule biosynthetic process |
| GO:0065008 | 2.00E-06 | regulation of biological quality |
| GO:0009706 | 2.00E-06 | chloroplast inner membrane |
| GO:0008652 | 2.30E-06 | cellular amino acid biosynthetic process |
| GO:0043933 | 2.40E-06 | macromolecular complex subunit organization |
| GO:0042440 | 2.80E-06 | pigment metabolic process |
| GO:0009725 | 3.40E-06 | response to hormone |
| GO:0033013 | 3.50E-06 | tetrapyrrole metabolic process |
| GO:0070271 | 3.60E-06 | protein complex biogenesis |
| GO:0043623 | 4.00E-06 | cellular protein complex assembly |
| GO:0044767 | 4.40E-06 | single-organism developmental process |
| GO:0009528 | 4.80E-06 | plastid inner membrane |
| GO:0005739 | 4.90E-06 | mitochondrion |
| GO:0046483 | 5.60E-06 | heterocycle metabolic process |
| GO:0042170 | 5.90E-06 | plastid membrane |
| GO:0043603 | 7.40E-06 | cellular amide metabolic process |
| GO:0003735 | 8.30E-06 | structural constituent of ribosome |
| GO:0016859 | 9.40E-06 | cis-trans isomerase activity |
| GO:0031969 | 9.60E-06 | chloroplast membrane |
| GO:0043207 | 1.10E-05 | response to external biotic stimulus |
| GO:0051707 | 1.10E-05 | response to other organism |
| GO:0071704 | 1.10E-05 | organic substance metabolic process |
| GO:0010190 | 1.10E-05 | cytochrome b6f complex assembly |
| GO:1901360 | 1.10E-05 | organic cyclic compound metabolic process |
| GO:0009719 | 1.20E-05 | response to endogenous stimulus |
| GO:0071214 | 1.20E-05 | cellular response to abiotic stimulus |
| GO:0032544 | 1.20E-05 | plastid translation |
| GO:0006461 | 1.50E-05 | protein complex assembly |
| GO:0009617 | 1.50E-05 | response to bacterium |
| GO:0044085 | 1.50E-05 | cellular component biogenesis |
| GO:0098542 | 1.50E-05 | defense response to other organism |
| GO:0072598 | 1.60E-05 | protein localization to chloroplast |
| GO:0045454 | 1.60E-05 | cell redox homeostasis |
| GO:0003755 | 1.60E-05 | peptidyl-prolyl cis-trans isomerase activity |
| GO:0005198 | 1.90E-05 | structural molecule activity |
| GO:0006732 | 2.00E-05 | coenzyme metabolic process |
| GO:0010287 | 2.20E-05 | plastoglobule |
| GO:0016667 | 2.20E-05 | oxidoreductase activity, acting on a sulfur group of donors |
| GO:0007275 | 2.30E-05 | multicellular organismal development |
| GO:0044707 | 2.60E-05 | single-multicellular organism process |
| GO:0010033 | 2.70E-05 | response to organic substance |
| GO:0009607 | 3.10E-05 | response to biotic stimulus |
| GO:0048856 | 3.20E-05 | anatomical structure development |
| GO:0009521 | 3.20E-05 | photosystem |
| GO:0010207 | 3.50E-05 | photosystem II assembly |
| GO:0044710 | 3.50E-05 | single-organism metabolic process |
| GO:0009416 | 3.60E-05 | response to light stimulus |
| GO:0006725 | 3.70E-05 | cellular aromatic compound metabolic process |
| GO:0009314 | 3.80E-05 | response to radiation |
| GO:0008237 | 3.80E-05 | metallopeptidase activity |
| GO:0016810 | 4.10E-05 | hydrolase activity, acting on carbon-nitrogen (but not peptide) bonds |
| GO:1901607 | 4.50E-05 | alpha-amino acid biosynthetic process |
| GO:0019725 | 5.20E-05 | cellular homeostasis |
| GO:0000413 | 5.30E-05 | protein peptidyl-prolyl isomerization |
| GO:0034660 | 5.60E-05 | ncRNA metabolic process |
| GO:0030154 | 6.00E-05 | cell differentiation |
| GO:0016053 | 6.20E-05 | organic acid biosynthetic process |
| GO:0046394 | 6.20E-05 | carboxylic acid biosynthetic process |
| GO:0005773 | 6.80E-05 | vacuole |
| GO:0033014 | 7.00E-05 | tetrapyrrole biosynthetic process |
| GO:0065003 | 7.10E-05 | macromolecular complex assembly |
| GO:0033554 | 7.30E-05 | cellular response to stress |
| GO:0015994 | 7.40E-05 | chlorophyll metabolic process |
| GO:0042742 | 7.40E-05 | defense response to bacterium |
| GO:0000741 | 7.60E-05 | karyogamy |
| GO:0009559 | 7.60E-05 | embryo sac central cell differentiation |
| GO:0010197 | 7.60E-05 | polar nucleus fusion |
| GO:0046148 | 8.20E-05 | pigment biosynthetic process |
| GO:0009295 | 8.30E-05 | nucleoid |
| GO:0010109 | 9.00E-05 | regulation of photosynthesis |
| GO:0045036 | 9.00E-05 | protein targeting to chloroplast |
| GO:0072596 | 9.00E-05 | establishment of protein localization to chloroplast |
| GO:0043604 | 9.10E-05 | amide biosynthetic process |
| GO:0044271 | 1.00E-04 | cellular nitrogen compound biosynthetic process |
| GO:0004222 | 1.00E-04 | metalloendopeptidase activity |
| GO:0009108 | 0.00011 | coenzyme biosynthetic process |
| GO:0042592 | 0.00012 | homeostatic process |
| GO:0006778 | 0.00012 | porphyrin-containing compound metabolic process |
| GO:0009523 | 0.00014 | photosystem II |
| GO:0018208 | 0.00019 | peptidyl-proline modification |
| GO:0009553 | 2.00E-04 | embryo sac development |
| GO:0048869 | 2.00E-04 | cellular developmental process |
| GO:0034622 | 0.00021 | cellular macromolecular complex assembly |
| GO:0009888 | 0.00022 | tissue development |
| GO:0006518 | 0.00023 | peptide metabolic process |
| GO:0051704 | 0.00026 | multi-organism process |
| GO:0009066 | 0.00027 | aspartate family amino acid metabolic process |
| GO:0048284 | 0.00028 | organelle fusion |
| GO:0044699 | 3.00E-04 | single-organism process |
| GO:0032501 | 0.00031 | multicellular organismal process |
| GO:0010555 | 0.00032 | response to mannitol |
| GO:0018065 | 0.00032 | protein-cofactor linkage |
| GO:0046686 | 0.00036 | response to cadmium ion |
| GO:0009561 | 0.00037 | megagametogenesis |
| GO:0043043 | 0.00039 | peptide biosynthetic process |
| GO:0051082 | 0.00039 | unfolded protein binding |
| GO:0019200 | 0.00039 | carbohydrate kinase activity |
| GO:0010038 | 0.00042 | response to metal ion |
| GO:0006412 | 0.00043 | translation |
| GO:0051716 | 0.00043 | cellular response to stimulus |
| GO:0048229 | 0.00043 | gametophyte development |
| GO:0009067 | 0.00043 | aspartate family amino acid biosynthetic process |
| GO:0004749 | 0.00043 | ribose phosphate diphosphokinase activity |
| GO:0016860 | 0.00048 | intramolecular oxidoreductase activity |
| GO:0009767 | 0.00049 | photosynthetic electron transport chain |
| GO:0006970 | 0.00049 | response to osmotic stress |
| GO:0010218 | 0.00051 | response to far red light |
| GO:0000096 | 0.00054 | sulfur amino acid metabolic process |
| GO:0006790 | 0.00055 | sulfur compound metabolic process |
| GO:0044238 | 6.00E-04 | primary metabolic process |
| GO:0010540 | 6.00E-04 | basipetal auxin transport |
| GO:0009117 | 0.00061 | nucleotide metabolic process |
| GO:1901700 | 0.00063 | response to oxygen-containing compound |
| GO:0006753 | 0.00065 | nucleoside phosphate metabolic process |
| GO:0009368 | 0.00065 | endopeptidase Clp complex |
| GO:0009840 | 0.00065 | chloroplastic endopeptidase Clp complex |
| GO:0015036 | 0.00066 | disulfide oxidoreductase activity |
| GO:0043094 | 0.00067 | cellular metabolic compound salvage |
| GO:0016597 | 0.00067 | amino acid binding |
| GO:0048731 | 7.00E-04 | system development |
| GO:0033558 | 0.00071 | protein deacetylase activity |
| GO:0034979 | 0.00071 | NAD-dependent protein deacetylase activity |
| GO:1901293 | 0.00072 | nucleoside phosphate biosynthetic process |
| GO:0055086 | 0.00081 | nucleobase-containing small molecule metabolic process |
| GO:0022607 | 0.00082 | cellular component assembly |
| GO:0008152 | 0.00082 | metabolic process |
| GO:0046653 | 0.00086 | tetrahydrofolate metabolic process |
| GO:0060429 | 0.00086 | epithelium development |
| GO:0009651 | 0.00089 | response to salt stress |
| GO:0022900 | 9.00E-04 | electron transport chain |
| GO:0071482 | 9.00E-04 | cellular response to light stimulus |
| GO:0009165 | 9.00E-04 | nucleotide biosynthetic process |
| GO:0043566 | 0.00092 | structure-specific DNA binding |
| GO:0042558 | 0.00096 | pteridine-containing compound metabolic process |
| GO:0042793 | 0.00104 | transcription from plastid promoter |
| GO:0000097 | 0.0011 | sulfur amino acid biosynthetic process |
| GO:0006997 | 0.00121 | nucleus organization |
| GO:0071470 | 0.00121 | cellular response to osmotic stress |
| GO:0090558 | 0.00123 | plant epidermis development |
| GO:0010026 | 0.00123 | trichome differentiation |
| GO:0010329 | 0.00129 | auxin efflux transmembrane transporter activity |
| GO:0005516 | 0.0013 | calmodulin binding |
| GO:0090407 | 0.00132 | organophosphate biosynthetic process |
| GO:0006760 | 0.00135 | folic acid-containing compound metabolic process |
| GO:0009642 | 0.0014 | response to light intensity |
| GO:0071478 | 0.00144 | cellular response to radiation |
| GO:0016811 | 0.00145 | hydrolase activity, acting on carbon-nitrogen (but not peptide) bonds, in linear amides |
| GO:0009668 | 0.00149 | plastid membrane organization |
| GO:0010027 | 0.00149 | thylakoid membrane organization |
| GO:0017004 | 0.00152 | cytochrome complex assembly |
| GO:0034470 | 0.00155 | ncRNA processing |
| GO:0016671 | 0.00157 | oxidoreductase activity, acting on a sulfur group of donors, disulfide as acceptor |
| GO:0001101 | 0.00164 | response to acid chemical |
| GO:0051181 | 0.00165 | cofactor transport |
| GO:0010021 | 0.00168 | amylopectin biosynthetic process |
| GO:0090377 | 0.00168 | seed trichome initiation |
| GO:2000896 | 0.00168 | amylopectin metabolic process |
| GO:0015037 | 0.00178 | peptide disulfide oxidoreductase activity |
| GO:0015038 | 0.00178 | glutathione disulfide oxidoreductase activity |
| GO:0007623 | 0.0018 | circadian rhythm |
| GO:0048511 | 0.0018 | rhythmic process |
| GO:0006555 | 0.00181 | methionine metabolic process |
| GO:0006779 | 0.00189 | porphyrin-containing compound biosynthetic process |
| GO:0008544 | 0.00205 | epidermis development |
| GO:0009765 | 0.00205 | photosynthesis, light harvesting |
| GO:0009913 | 0.00205 | epidermal cell differentiation |
| GO:0010020 | 0.00205 | chloroplast fission |
| GO:0030855 | 0.00205 | epithelial cell differentiation |
| GO:0045037 | 0.00205 | protein import into chloroplast stroma |
| GO:0010035 | 0.00215 | response to inorganic substance |
| GO:1901661 | 0.00219 | quinone metabolic process |
| GO:1901663 | 0.00219 | quinone biosynthetic process |
| GO:0071310 | 0.00222 | cellular response to organic substance |
| GO:0015995 | 0.00223 | chlorophyll biosynthetic process |
| GO:0043022 | 0.00224 | ribosome binding |
| GO:0048513 | 0.00225 | organ development |
| GO:0009814 | 0.00226 | defense response, incompatible interaction |
| GO:0044438 | 0.00227 | microbody part |
| GO:0044439 | 0.00227 | peroxisomal part |
| GO:0010319 | 0.0023 | stromule |
| GO:0004812 | 0.0024 | aminoacyl-tRNA ligase activity |
| GO:0016875 | 0.0024 | ligase activity, forming carbon-oxygen bonds |
| GO:0016876 | 0.0024 | ligase activity, forming aminoacyl-tRNA and related compounds |
| GO:0071265 | 0.00248 | L-methionine biosynthetic process |
| GO:0030267 | 0.00249 | glyoxylate reductase (NADP) activity |
| GO:0016730 | 0.00257 | oxidoreductase activity, acting on iron-sulfur proteins as donors |
| GO:0004175 | 0.00257 | endopeptidase activity |
| GO:0015035 | 0.00261 | protein disulfide oxidoreductase activity |
| GO:0016108 | 0.00262 | tetraterpenoid metabolic process |
| GO:0016116 | 0.00262 | carotenoid metabolic process |
| GO:0005840 | 0.0027 | ribosome |
| GO:0003975 | 0.0027 | UDP-N-acetylglucosamine-dolichyl-phosphate N-acetylglucosaminephosphotransferase activity |
| GO:0080025 | 0.0027 | phosphatidylinositol-3,5-bisphosphate binding |
| GO:0043572 | 0.00271 | plastid fission |
| GO:0019213 | 0.00273 | deacetylase activity |
| GO:0016109 | 0.00287 | tetraterpenoid biosynthetic process |
| GO:0016117 | 0.00287 | carotenoid biosynthetic process |
| GO:0070887 | 0.00289 | cellular response to chemical stimulus |
| GO:0006399 | 0.0029 | tRNA metabolic process |
| GO:0004407 | 0.00303 | histone deacetylase activity |
| GO:0017136 | 0.00303 | NAD-dependent histone deacetylase activity |
| GO:0031078 | 0.00303 | histone deacetylase activity (H3-K14 specific) |
| GO:0032041 | 0.00303 | NAD-dependent histone deacetylase activity (H3-K14 specific) |
| GO:0042181 | 0.00312 | ketone biosynthetic process |
| GO:0040007 | 0.00321 | growth |
| GO:0009662 | 0.00323 | etioplast organization |
| GO:0010361 | 0.00323 | regulation of anion channel activity by blue light |
| GO:0010362 | 0.00323 | negative regulation of anion channel activity by blue light |
| GO:0032365 | 0.00323 | intracellular lipid transport |
| GO:0042550 | 0.00323 | photosystem I stabilization |
| GO:0071258 | 0.00323 | cellular response to gravity |
| GO:0046835 | 0.0033 | carbohydrate phosphorylation |
| GO:0005778 | 0.00346 | peroxisomal membrane |
| GO:0031903 | 0.00346 | microbody membrane |
| GO:0015996 | 0.0035 | chlorophyll catabolic process |
| GO:0042548 | 0.0035 | regulation of photosynthesis, light reaction |
| GO:0046149 | 0.0035 | pigment catabolic process |
| GO:0042646 | 0.00351 | plastid nucleoid |
| GO:0070011 | 0.00355 | peptidase activity, acting on L-amino acid peptides |
| GO:0000373 | 0.00356 | Group II intron splicing |
| GO:0006014 | 0.00356 | D-ribose metabolic process |
| GO:0015937 | 0.00356 | coenzyme A biosynthetic process |
| GO:0009654 | 0.00366 | photosystem II oxygen evolving complex |
| GO:0008865 | 0.0038 | fructokinase activity |
| GO:0016783 | 0.0038 | sulfurtransferase activity |
| GO:0006575 | 0.00381 | cellular modified amino acid metabolic process |
| GO:0051536 | 0.00382 | iron-sulfur cluster binding |
| GO:0051540 | 0.00382 | metal cluster binding |
| GO:0009522 | 0.00386 | photosystem I |
| GO:0003697 | 0.00394 | single-stranded DNA binding |
| GO:0000287 | 0.00395 | magnesium ion binding |
| GO:0042430 | 0.00403 | indole-containing compound metabolic process |
| GO:0031406 | 0.00412 | carboxylic acid binding |
| GO:0043177 | 0.00412 | organic acid binding |
| GO:0016168 | 0.00416 | chlorophyll binding |
| GO:0030785 | 0.00418 | [ribulose-bisphosphate carboxylase]-lysine N-methyltransferase activity |
| GO:0005794 | 0.00419 | Golgi apparatus |
| GO:0005525 | 0.00421 | GTP binding |
| GO:0032561 | 0.00421 | guanyl ribonucleotide binding |
| GO:0006066 | 0.00422 | alcohol metabolic process |
| GO:0006415 | 0.00426 | translational termination |
| GO:0006476 | 0.00426 | protein deacetylation |
| GO:0009086 | 0.00426 | methionine biosynthetic process |
| GO:0035601 | 0.00426 | protein deacylation |
| GO:0016866 | 0.00444 | intramolecular transferase activity |
| GO:0043467 | 0.00445 | regulation of generation of precursor metabolites and energy |
| GO:0070932 | 0.00445 | histone H3 deacetylation |
| GO:0044270 | 0.00449 | cellular nitrogen compound catabolic process |
| GO:0019001 | 0.00453 | guanyl nucleotide binding |
| GO:0045087 | 0.00456 | innate immune response |
| GO:0005982 | 0.0046 | starch metabolic process |
| GO:0015095 | 0.00468 | magnesium ion transmembrane transporter activity |
| GO:1901615 | 0.00473 | organic hydroxy compound metabolic process |
| GO:0004747 | 0.00477 | ribokinase activity |
| GO:0009069 | 0.00488 | serine family amino acid metabolic process |
| GO:0006641 | 0.00491 | triglyceride metabolic process |
| GO:0033866 | 0.00491 | nucleoside bisphosphate biosynthetic process |
| GO:0034030 | 0.00491 | ribonucleoside bisphosphate biosynthetic process |
| GO:0034033 | 0.00491 | purine nucleoside bisphosphate biosynthetic process |
| GO:0031328 | 0.00502 | positive regulation of cellular biosynthetic process |
| GO:0042802 | 0.00509 | identical protein binding |
| GO:0006568 | 0.00512 | tryptophan metabolic process |
| GO:0006586 | 0.00512 | indolalkylamine metabolic process |
| GO:0033865 | 0.00512 | nucleoside bisphosphate metabolic process |
| GO:0033875 | 0.00512 | ribonucleoside bisphosphate metabolic process |
| GO:0034032 | 0.00512 | purine nucleoside bisphosphate metabolic process |
| GO:0009637 | 0.00517 | response to blue light |
| GO:0009072 | 0.00534 | aromatic amino acid family metabolic process |
| GO:0043648 | 0.00534 | dicarboxylic acid metabolic process |
| GO:0008236 | 0.00538 | serine-type peptidase activity |
| GO:0017171 | 0.00538 | serine hydrolase activity |
| GO:0006071 | 0.00539 | glycerol metabolic process |
| GO:0034508 | 0.00539 | centromere complex assembly |
| GO:0050821 | 0.00539 | protein stabilization |
| GO:0032870 | 0.00547 | cellular response to hormone stimulus |
| GO:0015936 | 0.00558 | coenzyme A metabolic process |
| GO:0034976 | 0.00558 | response to endoplasmic reticulum stress |
| GO:0071472 | 0.00558 | cellular response to salt stress |
| GO:0010467 | 0.00569 | gene expression |
| GO:0003723 | 0.00574 | RNA binding |
| GO:0000049 | 0.00577 | tRNA binding |
| GO:0019216 | 0.0061 | regulation of lipid metabolic process |
| GO:0044272 | 0.00612 | sulfur compound biosynthetic process |
| GO:0008299 | 0.00613 | isoprenoid biosynthetic process |
| GO:0071495 | 0.00614 | cellular response to endogenous stimulus |
| GO:0003690 | 0.00615 | double-stranded DNA binding |
| GO:0006139 | 0.0062 | nucleobase-containing compound metabolic process |
| GO:0000990 | 0.00624 | transcription factor activity, core RNA polymerase binding |
| GO:0000996 | 0.00624 | core DNA-dependent RNA polymerase binding promoter specificity activity |
| GO:0008878 | 0.00624 | glucose-1-phosphate adenylyltransferase activity |
| GO:0016987 | 0.00624 | sigma factor activity |
| GO:0005777 | 0.00625 | peroxisome |
| GO:0042579 | 0.00625 | microbody |
| GO:0009755 | 0.00639 | hormone-mediated signaling pathway |
| GO:0019637 | 0.0064 | organophosphate metabolic process |
| GO:0004316 | 0.00643 | 3-oxoacyl-[acyl-carrier-protein] reductase (NADPH) activity |
| GO:0015562 | 0.00643 | efflux transmembrane transporter activity |
| GO:0006955 | 0.00657 | immune response |
| GO:0009085 | 0.00657 | lysine biosynthetic process |
| GO:0009089 | 0.00657 | lysine biosynthetic process via diaminopimelate |
| GO:0009226 | 0.00657 | nucleotide-sugar biosynthetic process |
| GO:0046451 | 0.00657 | diaminopimelate metabolic process |
| GO:0006787 | 0.00689 | porphyrin-containing compound catabolic process |
| GO:0033015 | 0.00689 | tetrapyrrole catabolic process |
| GO:0044212 | 0.0069 | transcription regulatory region DNA binding |
| GO:0043038 | 0.00692 | amino acid activation |
| GO:0043039 | 0.00692 | tRNA aminoacylation |
| GO:0008233 | 0.00693 | peptidase activity |
| GO:0005829 | 0.00695 | cytosol |
| GO:0080134 | 0.00696 | regulation of response to stress |
| GO:0051537 | 0.00714 | 2 iron, 2 sulfur cluster binding |
| GO:0098732 | 0.00721 | macromolecule deacylation |
| GO:0009891 | 0.00729 | positive regulation of biosynthetic process |
| GO:1901565 | 0.00756 | organonitrogen compound catabolic process |
| GO:0045165 | 0.00775 | cell fate commitment |
| GO:0004333 | 0.00781 | fumarate hydratase activity |
| GO:0004412 | 0.00781 | homoserine dehydrogenase activity |
| GO:0004708 | 0.00781 | MAP kinase kinase activity |
| GO:0015417 | 0.00781 | polyamine-transporting ATPase activity |
| GO:0019902 | 0.00781 | phosphatase binding |
| GO:0019903 | 0.00781 | protein phosphatase binding |
| GO:0047661 | 0.00781 | amino-acid racemase activity |
| GO:0048487 | 0.00781 | beta-tubulin binding |
| GO:0080146 | 0.00781 | L-cysteine desulfhydrase activity |
| GO:0006418 | 0.00787 | tRNA aminoacylation for protein translation |
| GO:0006576 | 0.00787 | cellular biogenic amine metabolic process |
| GO:0048569 | 0.00811 | post-embryonic organ development |
| GO:0006364 | 0.00817 | rRNA processing |
| GO:0010196 | 0.00827 | nonphotochemical quenching |
| GO:0019400 | 0.00827 | alditol metabolic process |
| GO:0042372 | 0.00827 | phylloquinone biosynthetic process |
| GO:0042374 | 0.00827 | phylloquinone metabolic process |
| GO:1901259 | 0.00827 | chloroplast rRNA processing |
| GO:1990066 | 0.00827 | energy quenching |
| GO:0009791 | 0.00836 | post-embryonic development |
| GO:0051187 | 0.00839 | cofactor catabolic process |
| GO:0000162 | 0.00856 | tryptophan biosynthetic process |
| GO:0006553 | 0.00856 | lysine metabolic process |
| GO:0046219 | 0.00856 | indolalkylamine biosynthetic process |
| GO:0006302 | 0.00862 | double-strand break repair |
| GO:1901362 | 0.00863 | organic cyclic compound biosynthetic process |
| GO:0000975 | 0.00879 | regulatory region DNA binding |
| GO:0001067 | 0.00879 | regulatory region nucleic acid binding |
| GO:0016072 | 0.00885 | rRNA metabolic process |
| GO:0046700 | 0.00892 | heterocycle catabolic process |
| GO:0004448 | 0.00928 | isocitrate dehydrogenase activity |
| GO:0033817 | 0.00928 | beta-ketoacyl-acyl-carrier-protein synthase II activity |
| GO:0080161 | 0.00928 | auxin transmembrane transporter activity |
| GO:0006106 | 0.00932 | fumarate metabolic process |
| GO:0009249 | 0.00932 | protein lipoylation |
| GO:0009268 | 0.00932 | response to pH |
| GO:0010360 | 0.00932 | negative regulation of anion channel activity |
| GO:0019860 | 0.00932 | uracil metabolic process |
| GO:0032410 | 0.00932 | negative regulation of transporter activity |
| GO:0032413 | 0.00932 | negative regulation of ion transmembrane transporter activity |
| GO:0034763 | 0.00932 | negative regulation of transmembrane transport |
| GO:0034766 | 0.00932 | negative regulation of ion transmembrane transport |
| GO:0071457 | 0.00932 | cellular response to ozone |
| GO:0080005 | 0.00932 | photosystem stoichiometry adjustment |
| GO:1902047 | 0.00932 | polyamine transmembrane transport |
| GO:1903792 | 0.00932 | negative regulation of anion transport |
| GO:1903960 | 0.00932 | negative regulation of anion transmembrane transport |
| GO:1904143 | 0.00932 | positive regulation of carotenoid biosynthetic process |
| GO:0006396 | 0.00964 | RNA processing |
| GO:0009734 | 0.00969 | auxin-activated signaling pathway |

**Supplementary Table 8.** Transcripts identified in the functional annotations as part of flavonoid pathways. The gene name, KO number, definition, identified database, functional domain, gene ontology, e-value and coverage are shown

| **Transcripts identified in the flavonoid pathway** | | | | | | | |
| --- | --- | --- | --- | --- | --- | --- | --- |
| *Name* | *Entry KO* | *Definition* | *Database* | *Domain* | *GO* | *E-value* | *Coverage (%)* |
| 4CL | K01904 | 4-coumarate--CoA ligase | KEGG  (EC:6.2.1.12)  NCBI  (XP_028180032.1,  XP_006591122.1,  XP_003523137.1) | PFAM (AMP-binding), SMART (TRANS) | GO:0050896  GO:0008152  GO:0005488 | 9.3E-222 | 100 |
| CYP73A | K00487 | Trans-cinnamate 4-monooxygenase | KEGG (EC:[1.14.14.91](https://www.kegg.jp/entry/1.14.14.91))  NCBI (XP_020234620.1)  Swiss-Prot  (Q42797) | PFAM (p450), SMART (TRANS, SIGNAL) | GO:0003824  GO:0005488  GO:0009055 | 5.40e-280 | 99 |
| CHS | K00660 | Chalcone synthase | KEGG  (EC:[2.3.1.74](https://www.kegg.jp/entry/2.3.1.74))  NCBI  (XP_027360815.1)  Swiss-Prot  (P23569) | PFAM (Chal_sti_synt_C, ACP_syn_III_C, FAE1_CUT1_RppA) | GO:0003824  GO:0050896 GO:0065007 | 7.2E-216 | 100 |
| CHR | - | Chalcone reductase | NCBI  (NP_001353934.1) |  |  |  |  |
| FNSI | - | Flavone synthase I | Swiss-prot  (Q7XZQ8) | PFAM (2OG-FeII_Oxy, DUF569) | GO:0008152  GO:0003824 GO:0005488 | 8e-62 | 58 |
| HIDH | K13258 | 2-Hydroxyisoflavanone dehydratase | KEGG  (EC:[4.2.1.105](https://www.kegg.jp/entry/4.2.1.105))  NCBI  (XP_028180950.1)  NCBI  (XP_028180950.1)  Swiss-prot  (Q5NUF3) | PFAM (hydrolase_3, COesterase) | GO:0008152  GO:0003824 | 4.1E-161 | 100 |
| IF7GT | - | Isoflavone 7-O-glucosyltransferase | NCBI  (NP_001304440.2,  XP_028205885.1)  Swiss-Prot  (A6BM07) | PFAM (UDPGT) | GO:0008152  GO:0003824 | 8.4E-217 | 94 |
| IFR2 | - | Isoflavone reductase | NCBI  (XP_028227310.1) | PFAM (Epimerase, 3Beta_HSD) | GO:0050896  GO:0003824 | 1.4E-158 | 100 |
| CHI | K01859 | Chalcone isomerase | KEGG  (EC: 5.5.1.6)  NCBI  (XP_028229799.1,  XP_014513272.2,  XP_003592763.1,  XP_027937539.1)  Swiss-Prot  (Q8VZW3.  A4F1Q8,  Q53B75,  Q93XE6) | PFAM (Chalcone), SMART (COIL) | GO:0003824  GO:0008152 GO:0050896 | 2.3E-99 | 100 |
| F3H-2 | - | Flavanone 3-dioxygenase 2 | Swiss-Prot  (Q8W2X5) | PFAM (2OG-FeII_Oxy, DUF569) | GO:0003824 GO:0005488 | 4e-69 | 57 |
| FNSII  (CYP93B2) | K24188 | Flavone synthase II | KEGG  (EC:1.14.-.- )  Swiss-prot  (Q9XGT9) | PFAM (p450) | GO:0008152  GO:0044699- GO:0050896 | 6e-144 | 67 |
| CYP75B1 | - | Flavonoid 3'-monooxygenase | NCBI  (XP_028237345.1)  Swiss-Prot  (Q9SD85) | PFAM (p450) | GO:0003824 GO:0005488  GO:0009055 | 2e-176 | 99 |
| FLS | - | Flavonol synthase  (flavanone 3-hydroxylase) | Swiss-Prot  (Q9M547,  Q9ZWQ9) | PFAM (2OG-FeII_Oxy) | GO:0003824 GO:0005488  GO:0008152 | 1.00E-56 | 53 |
| GT6 | - | UDP-glucose flavonoid 3-O-glucosyltransferase 6 | Swiss-Prot  (Q2V6K0) | PFAM (UDPGT), SMART (COIL) | GO:0003824  GO:0008152 | 1.4E-113 | 95 |
| DFR | K00091 | Dihydroflavonol 4-reductase/flavanone 4-reductase | KEGG  (EC:[1.1.1.219](https://www.kegg.jp/entry/1.1.1.219))  NCBI (XP_028234880.1) Swiss-Prot  (P14721) | PFAM (Epimerase, NAD_binding_4) | GO:0008152 GO:0009987 GO:0065007 | 3.5E-171 | 100 |
| GT5 | x | Anthocyanidin 3-O-glucosyltransferase 5 | Swiss-Prot  (Q40287) | PFAM (UDPGT) | GO:0003824 | 3e-174 | 72 |
| RhGT1 | x | Anthocyanidin 5,3-O-glucosyltransferase | Swiss-Prot  (Q4R1I9) | PFAM (UDPGT, Glyco_tran_28_) | GO:0008152  GO:0003824 | 9e-102 | 58 |
| OMT1 | - | Flavone 3'-O-methyltransferase | Swiss-Prot  (Q9FK25) | PFAM (Methyltransf_2) | GO:0003824 GO:0005215 | 2e-105 | 67 |
| CYP75A6  (F3´5´H) | - | Flavonoid 3',5'-hydroxylase 6 | Swiss-Prot  (O04773)  NCBI  XP_028206768.1 | PFAM (p450), SMART (TRANS) | GO:0003824  GO:0005488 GO:0009055 | 4E-133 | 65 |

**Supplementary Table 9.** Down and up-regulated transcription factors (seeds versus leaves)

| **Down-regulated** | | | |
| --- | --- | --- | --- |
| ID | LogFC | Description | Subject |
| Evelu_DN2209_c0_g2_i1 | -10.26 | Auxin response factor | XP_020227198.1  AT5G37020.1 |
| Evelu_DN15088_c0_g1_i1 | -10.03 | NGA1-like | XP_027334900.1  AT2G46870.1 |
| Evelu_DN691_c0_g1_i1 | -8.74 | AP2/ERF family transcription factor | XP_007145470  AT5G07580.1 |
| Evelu_DN11319_c0_g1_i1 | -8.50 | Ethylene-responsive transcription factor | XP_020203062.1  AT1G19210.1 |
| Evelu_DN8465_c0_g1_i1 | -3.03 | Transcription factor UNE12 isoform X4 (bHLH) | XP_020237082.1  AT4G02590.2 |
| Evelu_DN9031_c0_g1_i1 | -3.20 | Phytochrome interacting factor 4 (PIF4) | AT2G43010.2 |
| Evelu_DN17421_c0_g1_i1 | -6.69 | Dof zinc finger protein DOF1.4-like isoform X1 | XP_027920737.1 AT1G28310.1 |
| Evelu_DN10982_c0_g1_i1 | -4.11 | Probable WRKY transcription factor 50 | XP_028233204.1  AT5G26170.1 |
|  | | | |
| **Up-regulated** | | | |
| ID | LogFC | Description | Subject |
| Evelu_DN8311_c0_g1_i1 | 2.37 | Basic helix-loop-helix (bHLH) family | AT2G42280.1  XP_014495363.1 |
| Evelu_DN8133_c0_g1_i1 | 12.08 | Trihelix transcription factor ASR3-like | AT2G35640.1  XP_014511899.1 |
| Evelu_DN14075_c0_g1_i1 | 10.40 | Uncharacterized protein LOC100500303 | NP_001267497.1  AT2G40350.1 |
| Evelu_DN3122_c0_g1_i1 | 6.96 | Transcription factor MYBC1 | XP_029127946.1  AT3G10760.1 |
| Evelu_DN3339_c0_g1_i1 | 5.27 | Heat stress transcription factor B-2b | XP_014521482.1 |

**Supplementary Table 10.** Transcription factors identified in *Erythrina velutina* leaves and seeds.

| **TFs** | **Number of genes (Leaves and Seeds)** | **Number of genes**  **(Only leaves)** | **Number of genes**  **(Only seeds)** |
| --- | --- | --- | --- |
| bHLH | 27 | 8 | 1 |
| ERF | 24 | 4 | 1 |
| C2H2 | 24 | 1 | 0 |
| MYB | 19 | 4 | 0 |
| bZIP | 19 | 1 | 0 |
| MYB_related | 18 | 2 | 0 |
| C3H | 16 | 0 | 1 |
| WRKY | 15 | 1 | 1 |
| Trihelix | 14 | 1 | 0 |
| NAC | 14 | 1 | 0 |
| GRAS | 13 | 0 | 3 |
| G2-like | 12 | 2 | 0 |
| FAR1 | 12 | 0 | 0 |
| HD-ZIP | 11 | 2 | 0 |
| GATA | 9 | 2 | 0 |
| Dof | 9 | 3 | 0 |
| TALE | 8 | 1 | 0 |
| HSF | 6 | 0 | 0 |
| B3 | 6 | 0 | 0 |
| NF-YC | 5 | 0 | 0 |
| BES1 | 5 | 0 | 0 |
| ZF-HD | 4 | 0 | 0 |
| TCP | 4 | 0 | 0 |
| NF-YB | 4 | 0 | 0 |
| LBD | 4 | 0 | 0 |
| Nin-like | 3 | 0 | 0 |
| HB-other | 3 | 0 | 0 |
| E2F/DP | 3 | 1 | 0 |
| ARR-B | 3 | 1 | 0 |
| ARF | 3 | 1 | 0 |
| AP2 | 3 | 0 | 0 |
| YABBY | 2 | 0 | 0 |
| WOX | 2 | 1 | 0 |
| SBP | 2 | 1 | 0 |
|  |  |  |  |
| NF-YA | 2 | 0 | 0 |
| M-type_MADS | 2 | 0 | 0 |
| MIKC_MADS | 2 | 3 | 0 |
| EIL | 2 | 0 | 0 |
| DBB | 2 | 0 | 0 |
| CPP | 2 | 0 | 0 |
| BBR-BPC | 2 | 0 | 0 |
| HB-PHD | 1 | 0 | 0 |
| GeBP | 1 | 0 | 0 |
| CO-like | 1 | 0 | 0 |
| CAMTA | 1 | 0 | 0 |
| SRS | 0 | 1 | 0 |
| LSD | 0 | 1 | 0 |
| **Total** | **344** | **43** | **7** |

**Supplementary Table 11.** Number of peptides and proteins identified after qualitative analysis of proteome vs. transcriptome

| **Proteome vs. transcriptome** | | | | | | | | |
| --- | --- | --- | --- | --- | --- | --- | --- | --- |
| **Identification of peptides (.sepro file)** | | | | | | | | |
| Organ  Variable | **LEAVES** | | | | **SEEDS** | | | |
|  | Acari I | Acari II | Jardim I | Jardim II | Acari I | Acari II | Jardim I | Jardim II |
| Proteins | 1211 | 1158 | 899 | 806 | 739 | 835 | 781 | 691 |
| Peptides | 3818 | 3489 | 2794 | 2472 | 2282 | 2555 | 2392 | 2123 |
| Spectra | 8109 | 6475 | 5402 | 6005 | 5715 | 6345 | 5824 | 5392 |
| Max parsimony | 909 | 867 | 674 | 618 | 514 | 578 | 569 | 492 |
| Proteins fusion I | 1388 | | 1060 | | 938 | | 870 | |
| Proteins fusion II | 1491 | | | | 1030 | | | |
| Max parsimony | 1152 | | | | 749 | | | |

**Supplementary Table 12.** Differentially abundant proteins identified after analysis in PatternLab for Proteomics (seeds x leaves)

| **ID transcriptome** | **FoldChange** | **p-value** | **Protein name** |
| --- | --- | --- | --- |
| Evelu_DN12878_c0_g1_i1 | -743.1245302 | 1.00E-05 | trypsin inhibitor 1A |
| Evelu_DN13297_c0_g1_i1 | -103.0014024 | 1.00E-05 | trypsin inhibitor 1A |
| Evelu_DN6832_c0_g1_i1 | -744.1706523 | 1.00E-05 | trypsin inhibitor 1A |
| Evelu_DN11493_c0_g1_i1 | -643.3124243 | 1.14E-05 | beta-conglycinin beta subunit 1 |
| Evelu_DN9962_c0_g1_i1 | -295.488128 | 4.30E-05 | Lectin |
| Evelu_DN10367_c0_g1_i1 | -5.349513788 | 0.0001346 | calreticulin |
| Evelu_DN14690_c0_g1_i1 | -10.82030307 | 0.0001436 | DNA damage-repair/toleration protein DRT100 |
| Evelu_DN8028_c0_g1_i1 | -13.18791648 | 0.0003569 | Chymotrypsin inhibitor 3 |
| Evelu_DN10646_c0_g1_i1 | -6.275900852 | 0.0003587 | serine hydroxymethyltransferase 4-like |
| Evelu_DN14312_c0_g1_i1 | -5.009438198 | 0.0011741 | x |
| Evelu_DN6465_c0_g1_i1 | 8.59813715 | 0.002206 | peroxiredoxin-2E, chloroplastic |
| Evelu_DN1953_c0_g1_i1 | -3.204478542 | 0.0022783 | probable UDP-arabinopyranose mutase 1 |
| Evelu_DN2135_c0_g1_i1 | -11.0978205 | 0.0024131 | x |
| Evelu_DN13732_c0_g2_i1 | 2.515249642 | 0.0026451 | aconitate hydratase, cytoplasmic |
| Evelu_DN13358_c0_g1_i1 | -3.977544559 | 0.0031772 | fructose-bisphosphate aldolase, cytoplasmic isozyme |
| Evelu_DN9323_c0_g1_i1 | 3.743919864 | 0.0043293 | Protein usf |
| Evelu_DN1067_c0_g1_i1 | -3.566942219 | 0.0051201 | tubulin beta-1 chain |
| Evelu_DN11399_c0_g1_i1 | -2.489501259 | 0.0058476 | Cysteine proteinase inhibitor 6 |
| Evelu_DN2534_c0_g1_i1 | -2.784650057 | 0.006232 | probable serine protease EDA2 |
| Evelu_DN14420_c0_g1_i1 | 4.623148371 | 0.0069693 | 14-3-3-like protein D isoform X1 |
| Evelu_DN2914_c0_g1_i1 | -2.58405657 | 0.0104872 | probable protein disulfide-isomerase A6 |
| Evelu_DN3098_c0_g1_i1 | 99.23186844 | 0.0135047 | phosphoglycerate kinase, cytosolic-like |
| Evelu_DN9781_c0_g1_i1 | -5.378649238 | 0.0138029 | aspartate aminotransferase 1 |
| Evelu_DN11944_c0_g1_i1 | 17.25988864 | 0.0143333 | plastid-lipid-associated protein, chloroplastic |
| Evelu_DN7731_c0_g1_i1 | 14.12837306 | 0.0153741 | 14-3-3-like protein A |
| Evelu_DN406_c0_g1_i1 | -3.17193303 | 0.0179166 | ribosomal protein L11 family protein |
| Evelu_DN4072_c0_g1_i1 | -4.596017873 | 0.0195712 | ricin B-like lectin R40G3 |
| Evelu_DN2590_c0_g1_i1 | -3.549290933 | 0.0217664 | elongation factor 2 |
| Evelu_DN14642_c0_g1_i1 | 4.655755118 | 0.0251534 | NAD(P)H dehydrogenase (quinone) FQR1-like |
| Evelu_DN16471_c0_g1_i1 | -20.64179948 | 0.0288505 | ran-binding protein 1 homolog c |
| Evelu_DN1491_c1_g1_i1 | -3.534061663 | 0.0339614 | superoxide dismutase [Fe], chloroplastic isoform X1 |


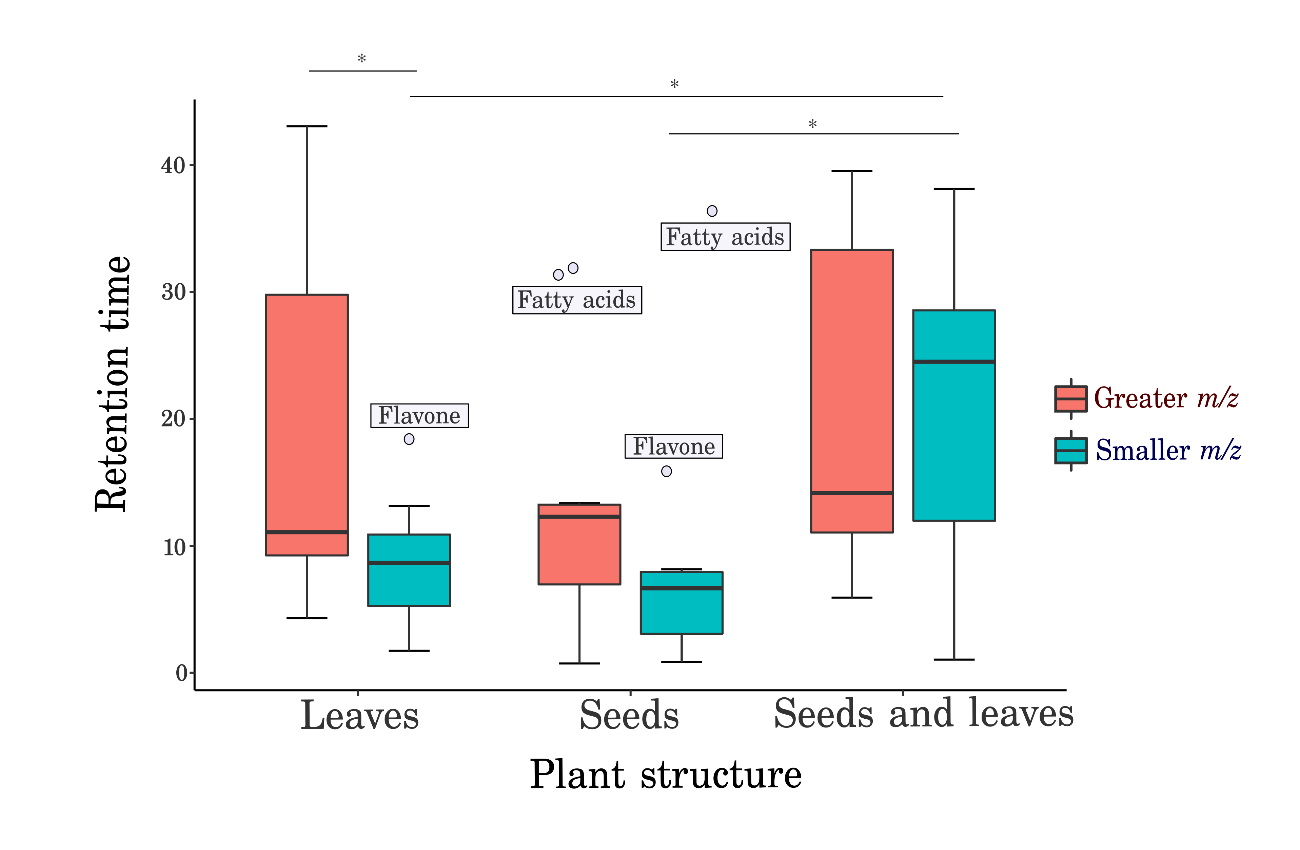


**Supplementary Figure 1.** F - Boxplot representing the distribution of common and exclusive metabolites of leaves and seeds with higher and lower values of mass/charge as a function of retention time. Asterisks represent statistical difference, according to the level of significance. Statistical analysis was performed using the GraphPad Prism 5 software (https://www.graphpad.com/scientific-software/prism/). The Shapiro-Wilk test was used as a normality test. The Dunn multiple comparison test was used followed by the non-parametric Mann-Whitney test. The significance level was defined as p ≤ 0.05 with a 95% confidence interval.


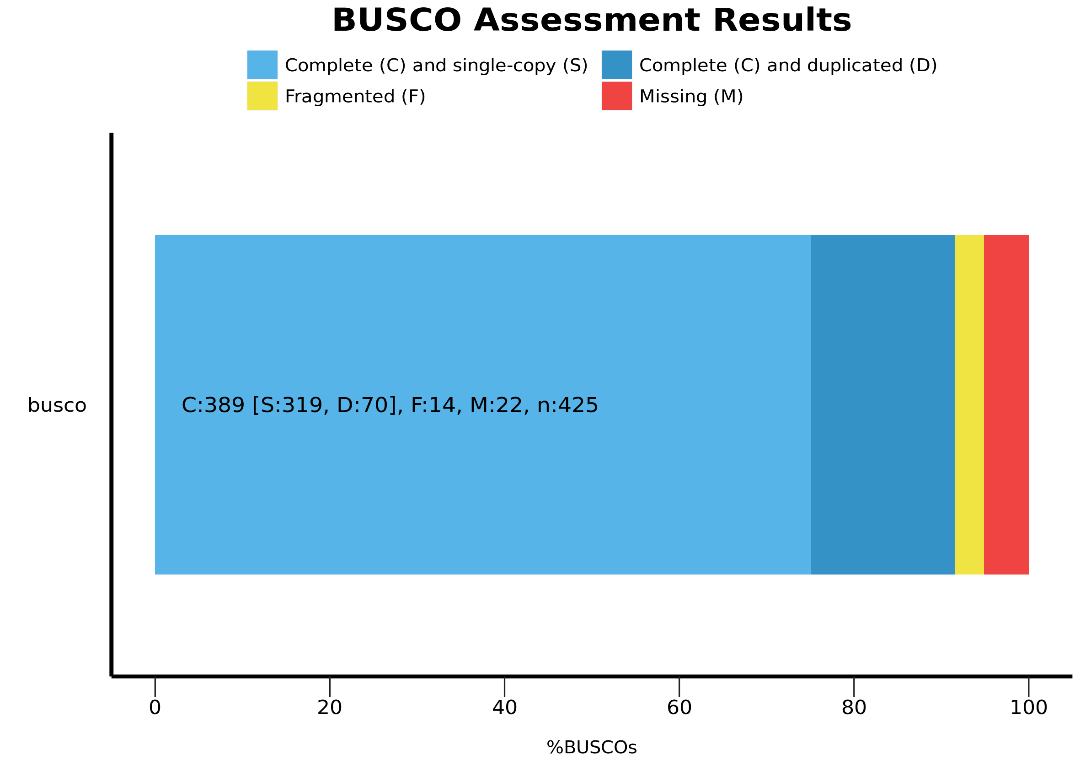


**Supplementary Figure 2.** Result of the analysis of BuscoV3 for individual leaf assembly. C:91.6% [ S:75.1%, D:16.5%], F:3.3%, M:5.1%, n:425. 389 complete BUSCOs (C); 319 Complete and single-copy BUSCOs (S); 70 Complete and duplicated BUSCOs (D); 14 Fragmented BUSCOs (F); 22 Missing BUSCOs (M).


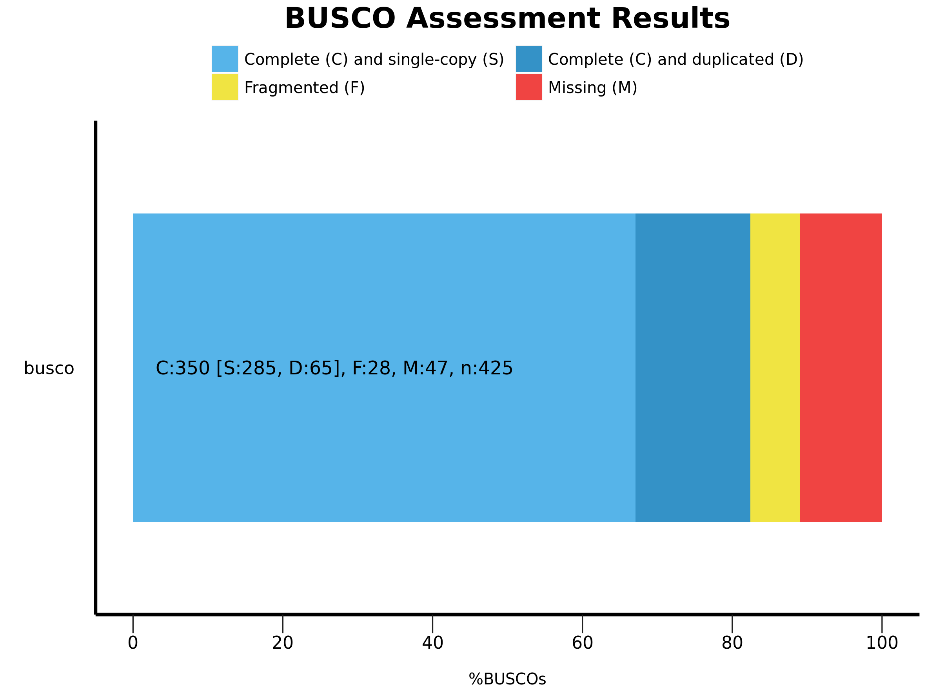


**Supplementary Figure 3.** Result of the analysis of BuscoV3 for individual seed assembly. C:82.4% [S:67.1%, D:15.3%], F:6.6%, M:11.0%, n:425. 350 Complete BUSCOs (C); 285 Complete and single-copy BUSCOs (S); 65 Complete and duplicated BUSCOs (D); 28 Fragmented BUSCOs (F); 47 Missing BUSCOs (M).


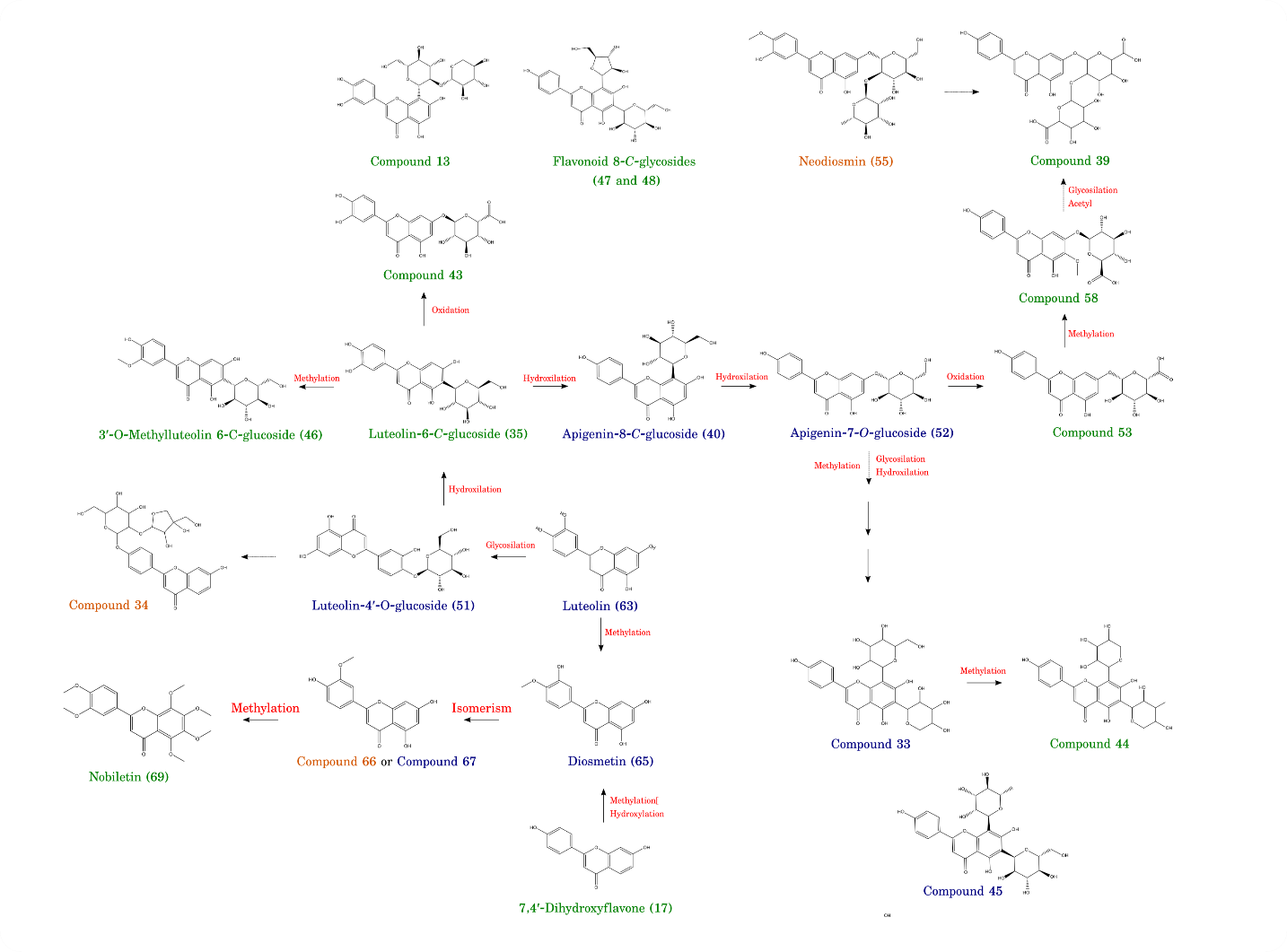


**Supplementary Figure 4.** Flavones identified in the metabolic profile and annotated in GNPS. Compounds written in blue, green, and orange represent the metabolites present in leaves and seeds, just leaves, and just seeds, respectively.


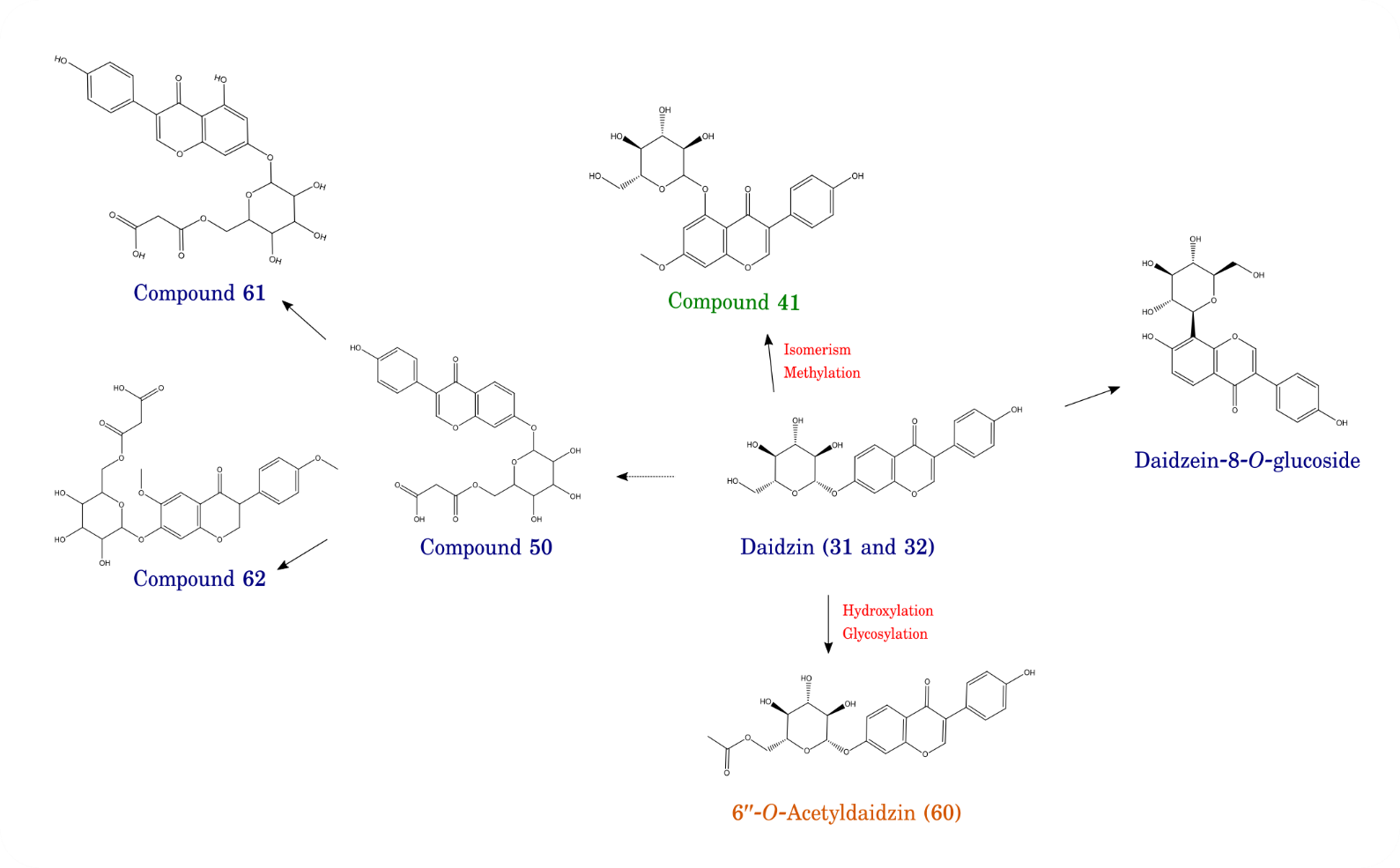


**Supplementary Figure 5.** Isoflavones identified in the metabolic profile and annotated in GNPS. Compounds written in blue, green, and orange represent the metabolites present in leaves and seeds, just leaves, and just seeds, respectively.


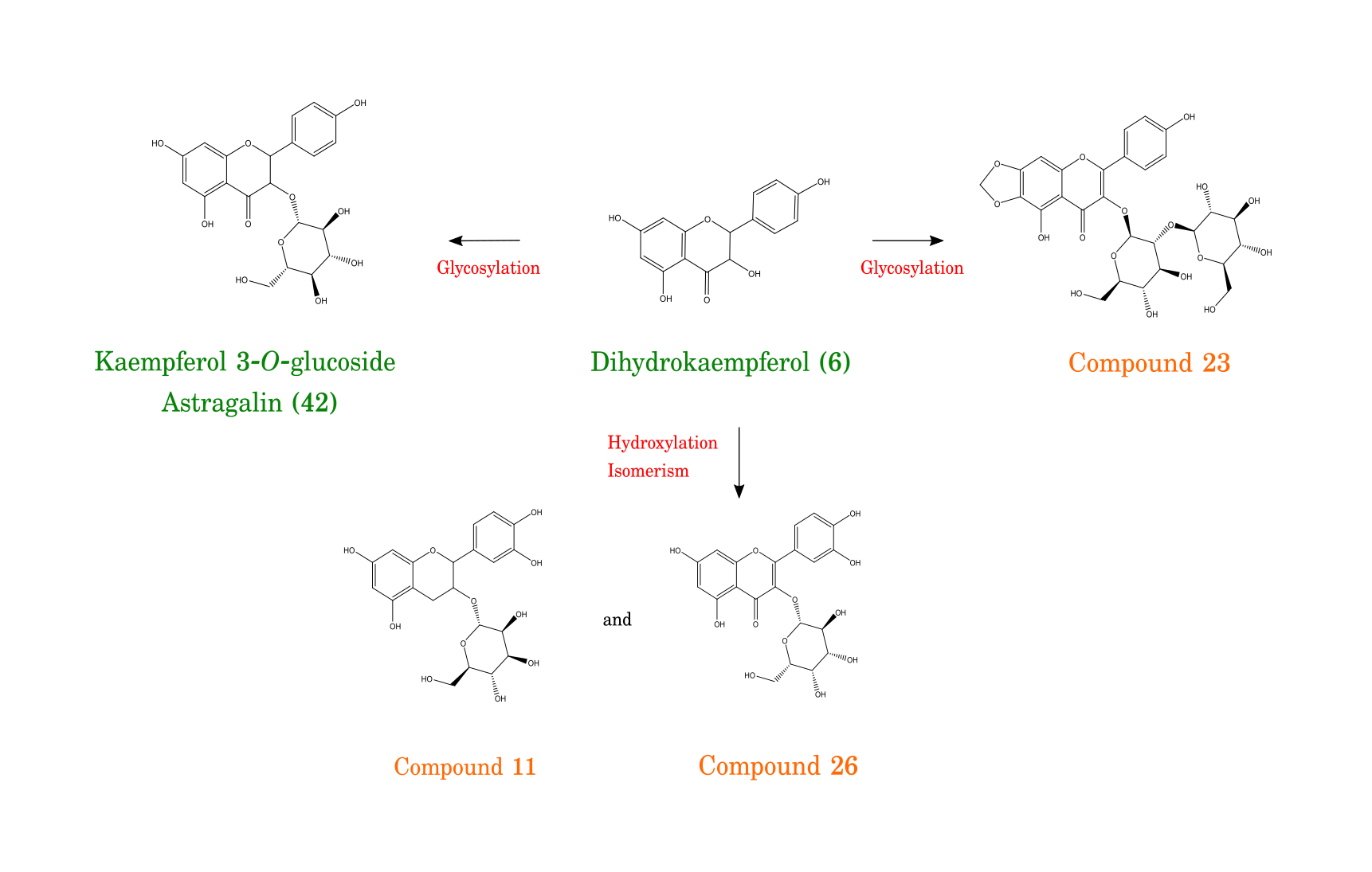


**Supplementary Figure 6.** Flavonols identified in the metabolic profile and annotated in GNPS. Compounds written in blue, green, and orange represent the metabolites present in leaves and seeds, just leaves, and just seeds, respectively.


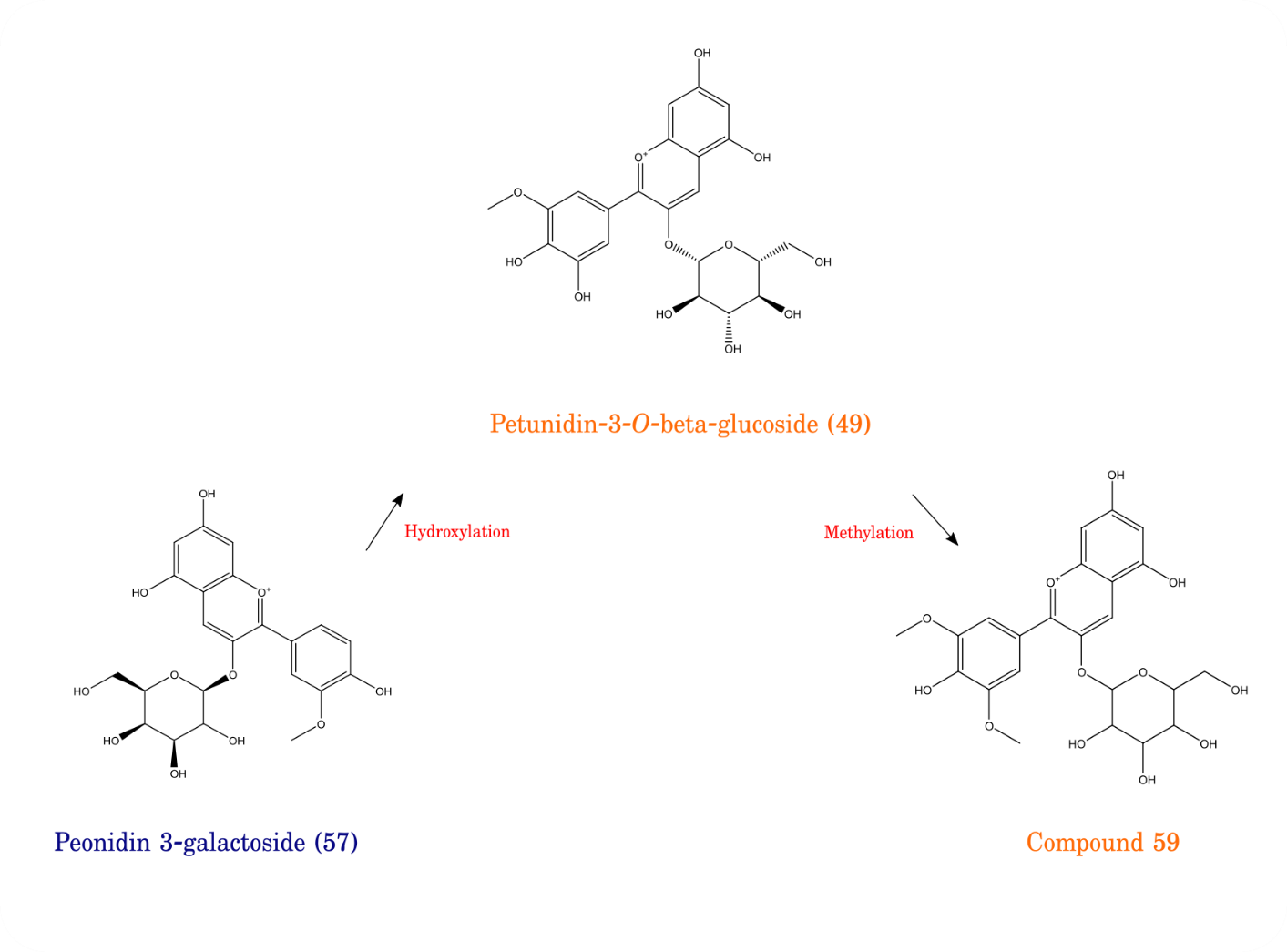


**Supplementary Figure 7.** Anthocyanidin identified in the metabolic profile and annotated in GNPS. Compounds written in blue, green, and orange represent the metabolites present in leaves and seeds, just leaves, and just seeds, respectively.


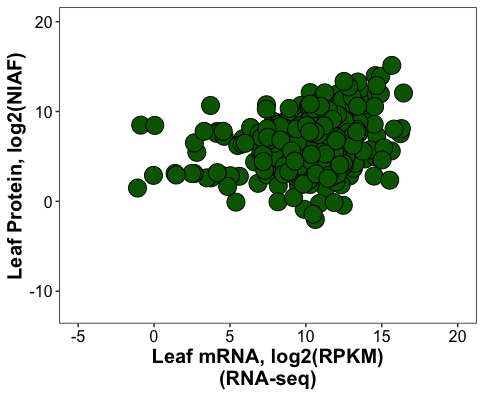


**Supplementary Figure 8.** Leaves correlation analysis. 388 proteins/DETs mRNAs. Pearson: 0.35 and p-value: 1.13e-12 | Spearman: 0.24 and p-value: 1.82e-06.


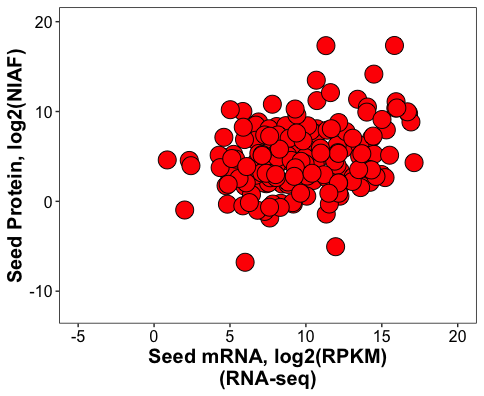


**Supplementary Figure 9.** Seeds correlation analysis. 170 proteins/DETs mRNAs. Pearson: 0.12 and p-value: 0.104 | Spearman: 0.24 p-value: 0.001


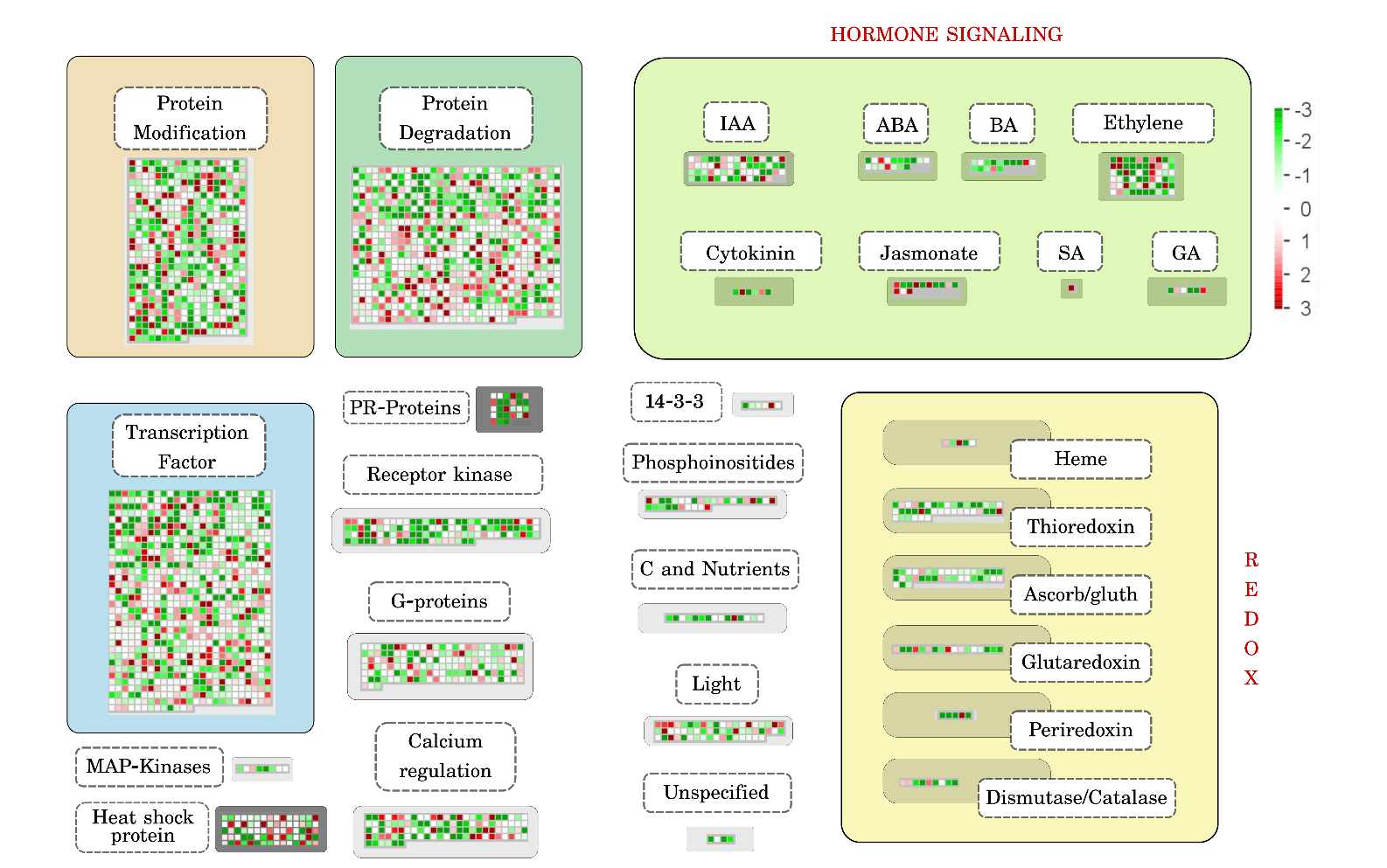


**Supplementary Figure 10.** Overview of metabolism regulation in leaves and seeds of *Erythrina velutina.* Fold Change values are represented in color in Log2. Each square represents a transcript that encodes the regulated protein. Down-regulated is represented in green, and up-regulated represented in red squares, both in seeds versus leaves.


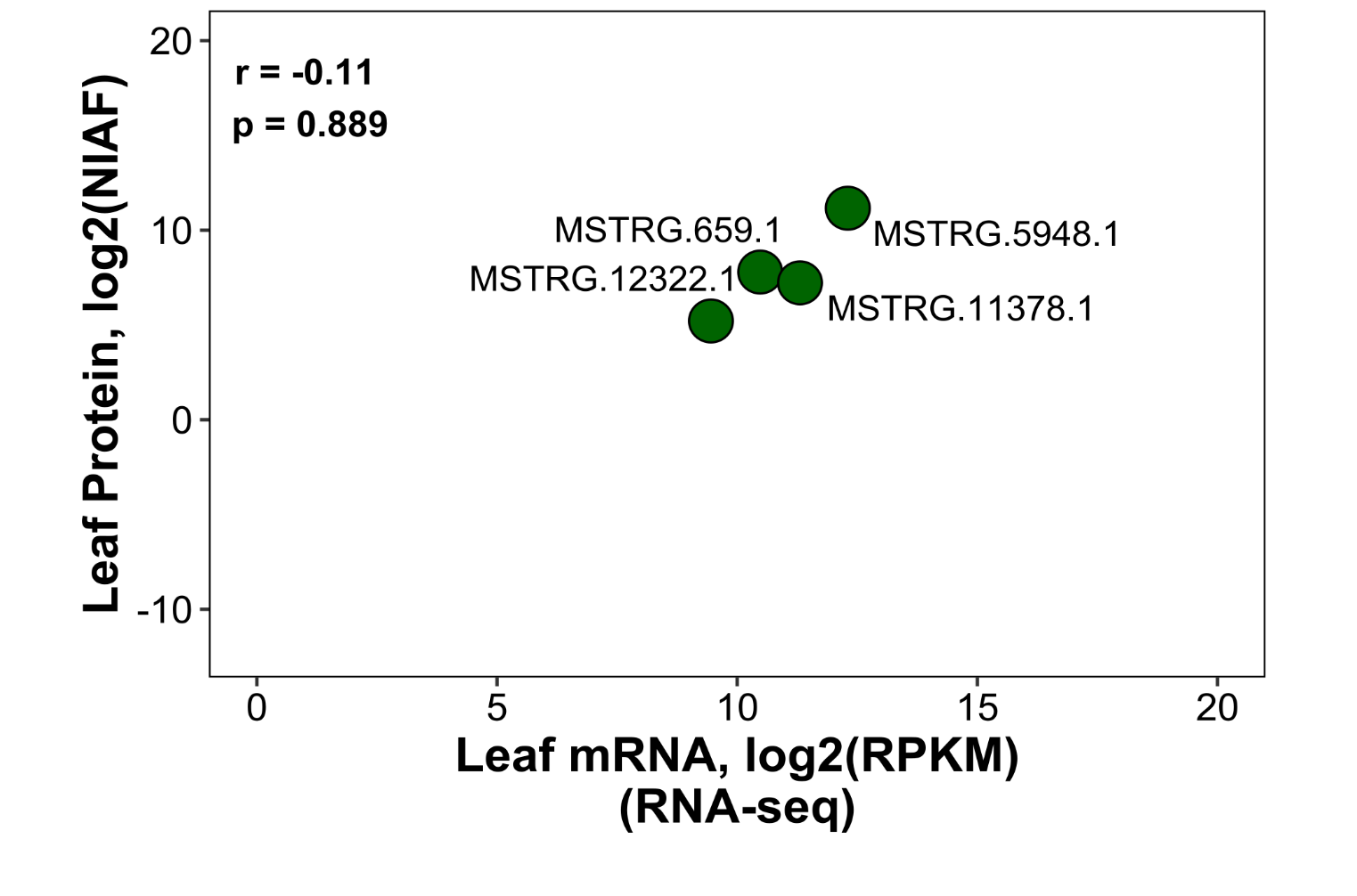


**Supplementary Figure 11.** Transcripts identified in leaves and their respective proteins with significant NIAF involved in the flavonoid biosynthesis pathway.


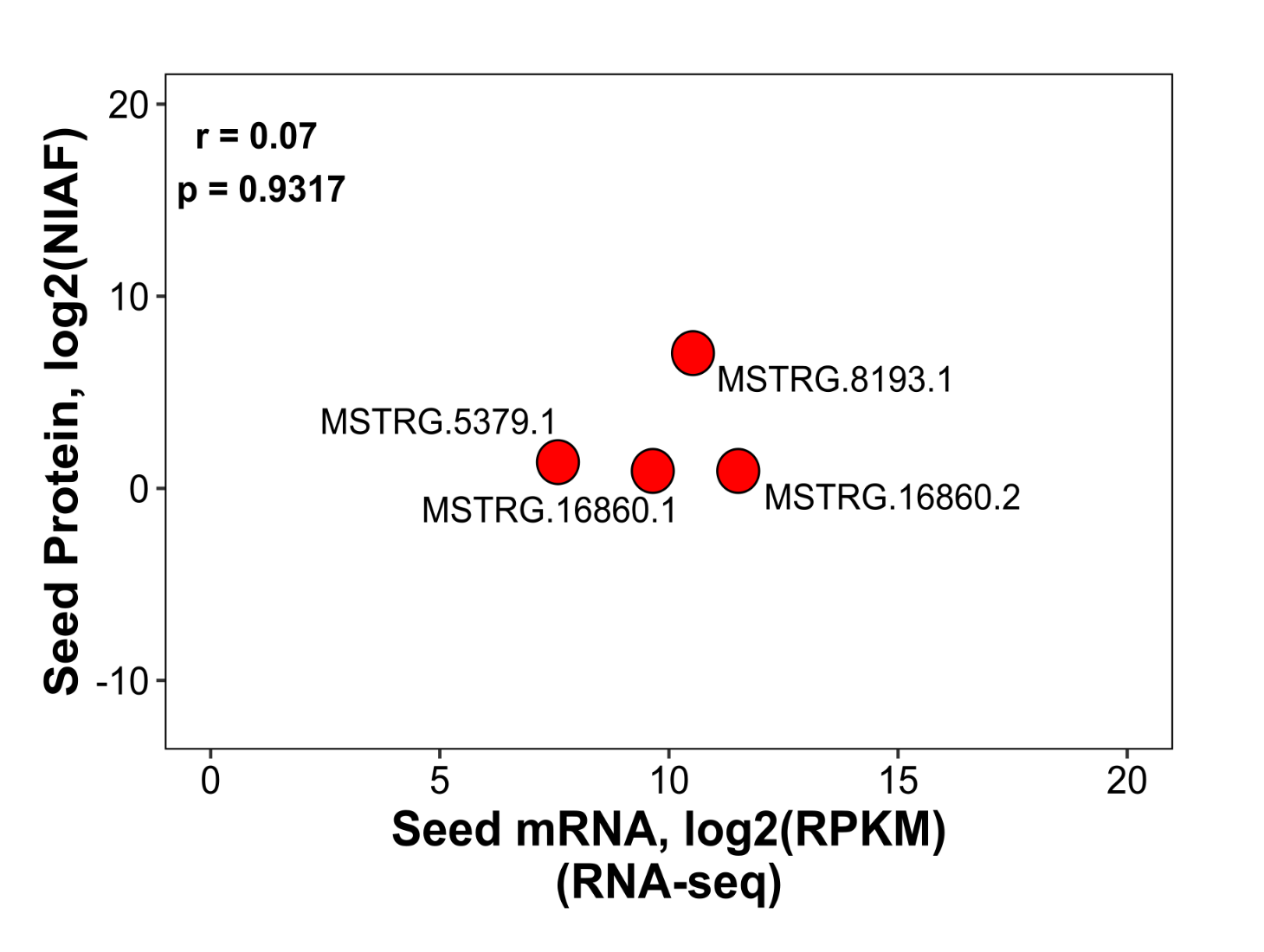


**Supplementary Figure 12.** Transcripts identified in seeds and their respective proteins with significant NIAF involved in the flavonoid biosynthesis pathway.


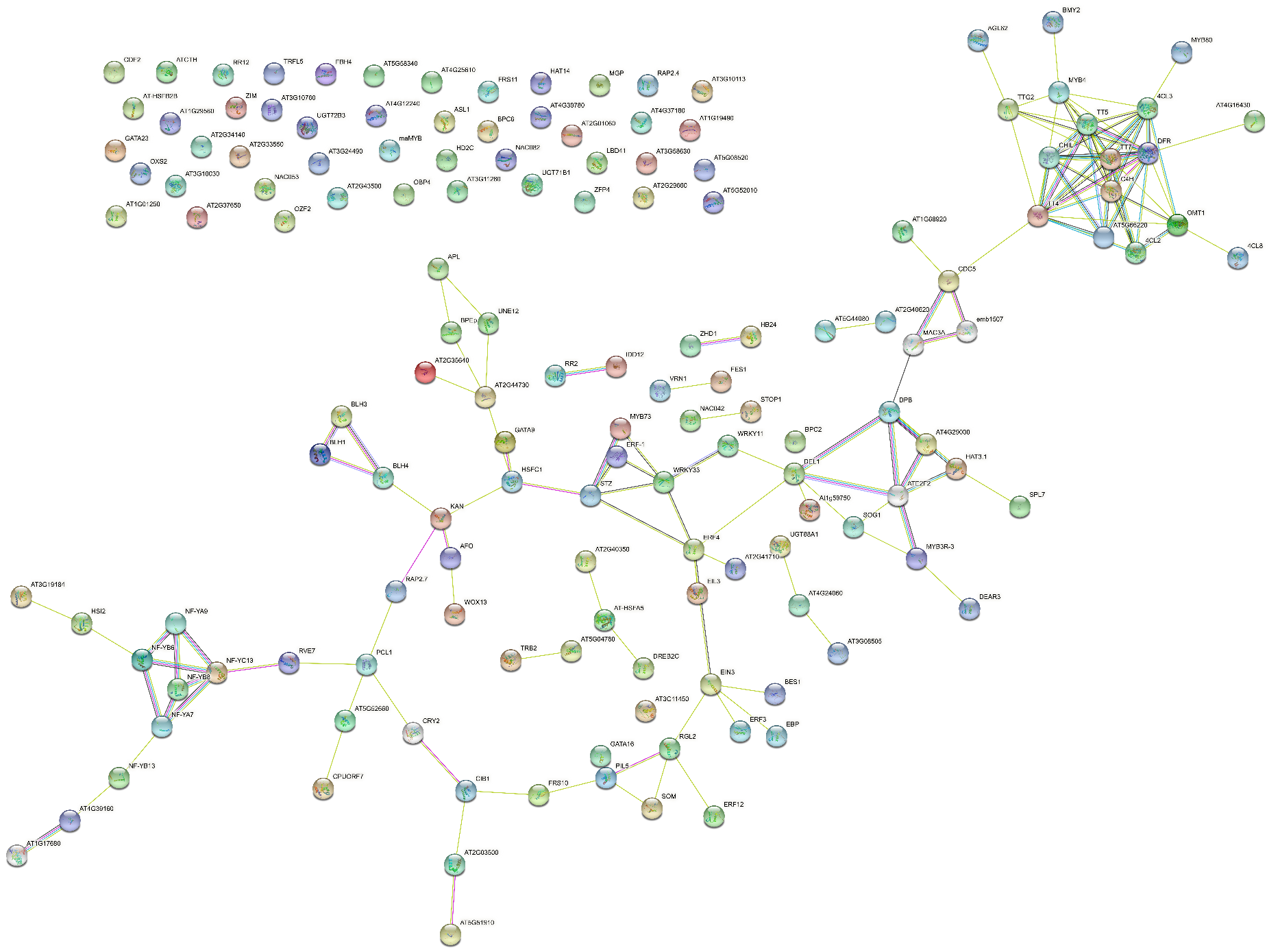


**Supplementary Figure 13:** Interaction network of putative transcripts up-regulated and transcription factors involved in flavonoid biosynthesis. Each node represents an identified protein and these are interconnected behind lines (or borders) that jointly contribute to a shared function and classified as: 1 - Known interactions (light blue and purple lines are proteins identified from databases and experimentally, respectively); 2 - Predicted interactions (green, red and medium blue lines represent neighborhood gene, gene fusions and gene co-occurrence, respectively); and 3 - Others (yellow and dark blue lines represent protein co-expression and homology, respectively).


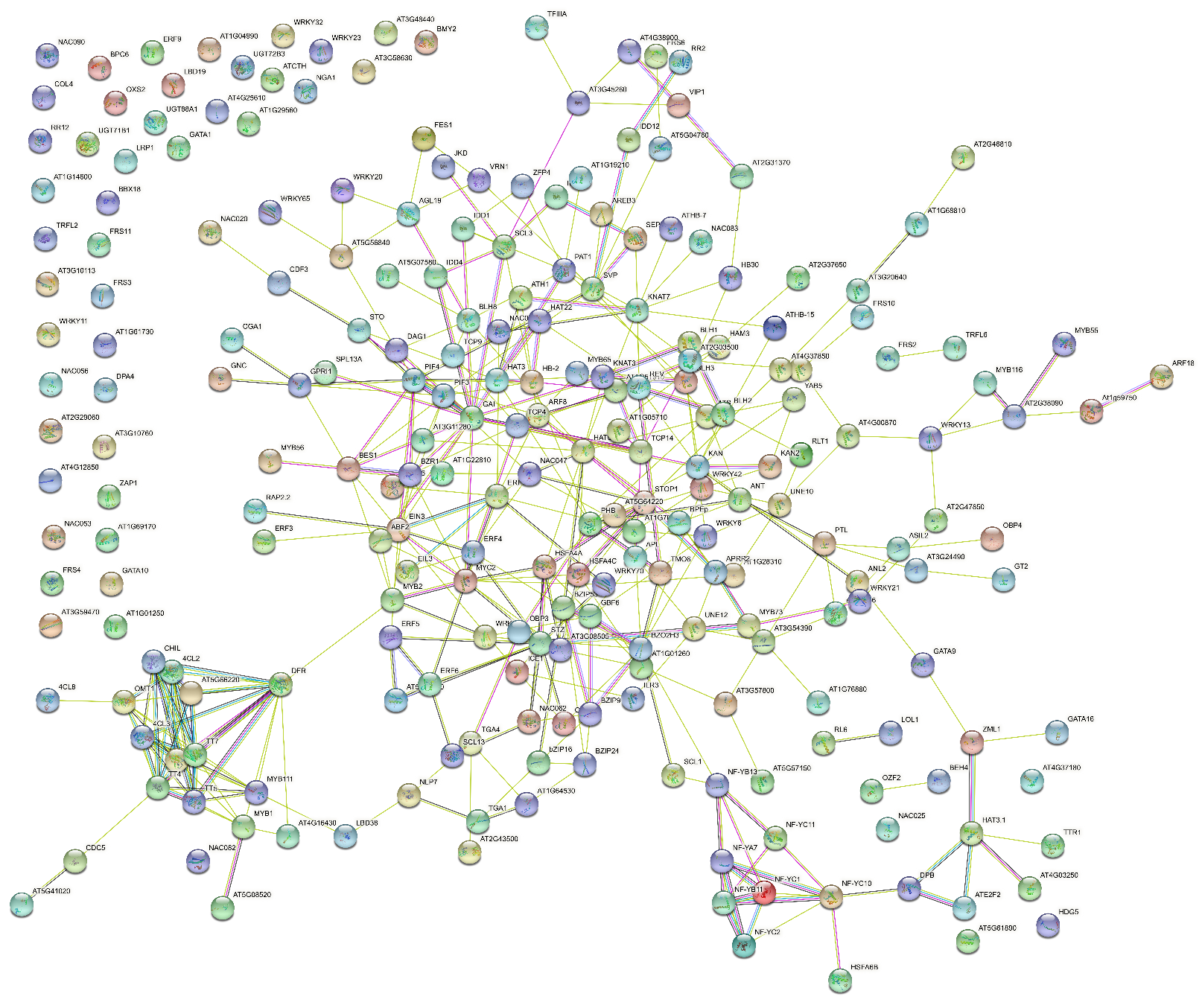


**Supplementary Figure 14:** Interaction network of putative transcripts down-regulated and transcription factors involved in flavonoid biosynthesis. Each node represents an identified protein and these are interconnected behind lines (or borders) that jointly contribute to a shared function and classified as: 1 - Known interactions (light blue and purple lines are proteins identified from databases and experimentally, respectively); 2 - Predicted interactions (green, red and medium blue lines represent neighborhood gene, gene fusions and gene co-occurrence, respectively); and 3 - Others (yellow and dark blue lines represent protein co-expression and homology, respectively).
